# Protein Molecular Surface Mapped at Different Geometrical Resolutions

**DOI:** 10.1371/journal.pone.0058896

**Published:** 2013-03-14

**Authors:** Dan V. Nicolau, Ewa Paszek, Florin Fulga, Dan V. Nicolau

**Affiliations:** 1 Department of Electrical Engineering & Electronics, University of Liverpool, Liverpool, United Kingdom; 2 Department of Bioengineering, McGill University, Montreal, Canada; 3 Department of Integrative Biology, University of California, Berkeley, California, United States of America; King's College, London, United Kingdom

## Abstract

Many areas of biochemistry and molecular biology, both fundamental and applications-orientated, require an accurate construction, representation and understanding of the protein molecular surface and its interaction with other, usually small, molecules. There are however many situations when the protein molecular surface gets in physical contact with larger objects, either biological, such as membranes, or artificial, such as nanoparticles. The contribution presents a methodology for describing and quantifying the molecular properties of proteins, by geometrical and physico-chemical mapping of the molecular surfaces, with several analytical relationships being proposed for molecular surface properties. The relevance of the molecular surface-derived properties has been demonstrated through the calculation of the statistical strength of the prediction of protein adsorption. It is expected that the extension of this methodology to other phenomena involving proteins near solid surfaces, in particular the protein interaction with nanoparticles, will result in important benefits in the understanding and design of protein-specific solid surfaces.

## Introduction

Many areas of biochemistry and molecular biology, both fundamental, e.g., protein folding [1], protein conformational stability [2], inter- and intra- protein interactions [3], molecular recognition [4] and docking [5]; as well as applications-orientated, e.g., drug design [6], [7], protein and peptide solubility [8], crystal packing [9], and enzyme catalysis [10]; require an accurate construction, representation and understanding of the protein molecular surface and its interaction with other, usually small, molecules.

The applications enumerated above, almost exclusively focused on biomolecular interactions, necessitate the construction of the molecular surface at a resolution scale similar to the size of the molecule that interacts with the protein, e.g., up to 5Å, which is approximately the dimension of a large solvent molecule. There are however many situations when the protein molecular surface is in physical contact with larger objects, either biological or artificial. For instance, many biomolecular interactions occur on cell membranes, e.g., involving lipid rafts with sizes much larger than that of the water molecule [11]. Also, the long range self-assembly of proteins, e.g., cytoskeleton formation [12], formation of amyloid plaques and tangles [13], occurs through biomolecular recognition of larger areas on the molecular surface. Biomolecules also interact with solid surfaces on which they are immobilized, either by design, or unintentionally [14], [15], for applications as diverse as biomaterials [14], [16], chromatography [17] membrane research [18], [19], biomedical micro- and nano-devices [20], [21], such as biosensors [22], microarrays [23], [24] and lab-on-a-chip devices [25], where the preservation of the bioactivity of the immobilized proteins is paramount. More recently, nanoparticle research has become interested in the study of the interaction between proteins and artificial objects similar with their size, or larger [26]. Indeed, the nanoparticle:protein interaction can either amplify the beneficial effects of nanoparticles, e.g., protein aggregation around a nanoparticle can create a ‘protein corona’ [27], [28], which could be essential for the nanoparticle uptake in the cell, where its therapeutic action can unfold [29]; or conversely, it can induce the change of conformation and consequently the bioactivity of the proteins attached to the nanoparticle [30], [31], thus cascading in nanoparticle-induced nanotoxicity [32], [33].

The probing of the protein molecular surface with probes with larger radii has also fundamental motivations. The general consensus regarding the protein structure is based on the concept of the “hydrophobic core,” which states that the hydrophobic amino acids aggregate, via hydrophobic-hydrophobic attraction, towards the core of the protein, leaving the outbound protein sheath more hydrophilic. This central concept needs constant and thorough qualification, as proteins have extremely diverse and complex geometries. Recently, several reports [34]–[36], which tested the “hydrophobic core” concept using fractal analysis, found that all the major structural classes of proteins have an amount of ‘unused’ hydrophobicity, thus showing that they are not as optimally packed as they are supposed to be. The representation of biomolecular surfaces, especially for proteins, encounters serious difficulties, even for the simplest globular proteins, due to the complexity, lack of symmetry and irregularity of the distribution of atoms. The construction of the protein molecular surfaces uses its biomolecular structure [37], revealed through X-ray crystallography or NMR studies and archived in databases, such as Protein Database, PDB [38]. The first algorithms employed for the construction of molecular surfaces [39]–[41] used virtual probes to determine the position of the points of contact with protein atoms, thus generating an ‘envelope’ of the protein, which is a representation of its molecular surface. While other algorithms [4], [42]–[62] for the construction of molecular surfaces are reported to be more computer resources-efficient, the use of virtual probes has the conceptual advantage of being more intuitive, as the *in silico* probing mimics specific or non-specific biomolecular events.

The ubiquity and importance of these interactions, for reasons that are both fundamental and industrial, e.g., pharmaceutical, biomaterials, biomedical devices, suggest that the accurate representation of protein molecular surfaces as probed by probes with large dimensions is fully warranted. To this end, the aim of this contribution is to assess the impact of the probing resolution on the construction of protein molecular surfaces; demonstrate the benefits of this approach for the understanding of the interaction between proteins and large nano-objects; and propose new research avenues that can capitalize on this methodology.

## Methods

### Proteins

The structures of 35 proteins (Table 1) have been selected from the Protein Data Bank [38]. The set spans several representative sets of proteins, namely lactalbumin (dataset 1, in Table 1), lactoglobulin (dataset 2), lysozyme (dataset 3), ribonuclease (dataset 4), hemoglobin (dataset 5), albumin (dataset 6) and antibody (dataset 7). The protein datasets also cover a large range of molecular weights (14 to 148 kDa), residues (123 to 1344), isoelectric points (4.5 to 11) and shapes (globular, ellipsoidal, Y-shaped). Five representative proteins, i.e., lysozyme, ribonuclease, hemoglobin, albumin and IgG (in bold in Table 1) have been selected for graphical presentations. Complete results, with conclusions in accord with those for the selected proteins, are presented in File S1.

**Table 1:** Proteins used for the analysis of molecular surfaces.

Dataset No. RMSD(residues)	No.	Protein name	PDB code	Atoms	Residues	Chains
1	1	*α lactalbumin*	1A4V	1092	123	1
2	2	porcine β-lactoglobulin	1EXS	1248	160	1
	3	*bovine β-lactoglobulin*	1BEB	2473	324	2
**3, Subset 3.1** RMSD(129) = 0.6481	4	**hen egg-white lysozyme**	1LYZ	1001	129	1
	5	turkey egg-white lysozyme	135L	994	129	1
	6	hen egg-white lysozyme	2LYM	1001	129	1
	7	triciclic lysozyme	2LZT	1001	129	1
**3, Subset 3.2** RMSD(164) = 0.24	8	mutant of phage T4 lysozyme	1L35	1305	164	1
	9	T4 lysozyme	1LYD	1309	164	1
4 RMSD(124) = 0.1655	10	ribonuclease-A	8RAT	951	124	1
	11	ribonuclease-A	1RBX	956	124	1
	12	bovine ribonuclease-A	3RN3	957	124	1
	13	**ribonuclease-A**	1AFU	1894	248	2
**5, Subset 5.1** RMSD(287) = 0.8877	14	human oxyhemoglobin	1HHO	2192	287	2
	15	human carbonmonoxy hemoglobin	2HCO	2192	287	2
	16	horse deoxyhemoglobin	2DHB	2201	287	2
**5, Subset 5.2** RMSD(574) = 1.501	17	human hemoglobin A	1BUW	4342	574	4
	18	**human hemoglobin (W37A)**	**1Y4F**	4368	574	4
	19	hemoglobin mutant (W37A)	**1A01**	4368	574	4
	20	human hemoglobin (W37E)	**1Y4P**	4376	574	4
	21	hemoglobin mutant (W37Y)	**1A00**	4382	574	4
	22	human hemoglobin (W37Y)	**1Y46**	4382	574	4
	23	human deoxyhemoglobin	2HHB	4384	574	4
	24	human hemoglobin (W37G)	**1Y4G**	4366	574	4
	25	hemoglobin mutant (V1M)	**1A0U**	4386	574	4
	26	hemoglobin mutant (V1M)	**1A0Z**	4386	574	4
	27	recombinant hemoglobin	1C7D	4396	576	3
6 RMSD(585) = 2.3740	28	human serum albumin complexed with octadecanoic acid	1E7I	4496	585	1
	29	recombinant human serum albumin	1UOR	4617	585	1
	30	human serum albumin	1E78	4302	585	1
	31	**human serum albumin**	1AO6	4600	585	1
	32	human serum albumin	1BM0	4600	585	1
7	33	immunoglobulin	1IGY	10002	1294	4
	34	immunoglobulin	1IGT	10196	1316	4
	35	**intact human IgG B12**	1HZH	10355	1344	4

To quantify the similarity between the members of a class, for all sets, or subsets, i.e., for lysozyme and hemoglobins, the Root-Mean-Square Deviation (RMSD) has been calculated using the protein structure comparison service Fold at the European Bioinformatics Institute (http://www.ebi.ac.uk/msd-srv/ssm).[63]

A subset of the hemoglobin class,[64] comprising eight mutant structures of the deoxy forms of the protein, with the same number of residues (574), but with (i) the Trp37 residue, i.e., 1A0U and 1A0Z, for the crystal form 1 and 2, respectively; and with residues replacing the Trp37 residue by (ii) Tyr37, i.e., structures 1Y46 and 1A00, for crystal 1 and 2, respectively; (iii) Ala37, i.e., 1Y4F and 1A01, for crystal form 1 and 3, respectively; (iv) Glu37, i.e., 1Y4P, for crystal form 1; and (v) Gly37, i.e., 1Y4G for crystal form 1; was used to test the fine definition of the molecular surface for very similar proteins. This sub-set is indicated in bold in Table 1. The full results regarding the quantification of properties on the molecular surfaces are presented in File S2 and the results regarding the quantitative measure of the sequence (sequence identity) and structural (RMSD after structural alignment where possible) are also presented in File S3.

### Treatment of charges

The charges of the atoms in amino acids have been calculated applying a semi empirical method (PM3, as implemented in HyperChem, from HyperCube Inc.) on model tripeptides Gly-X-Gly (where X is the respective amino acid), following a geometry optimization step that used a molecular mechanics force field (Amber, as implemented in HyperChem). The charges have been calculated for the whole range of pH in 0.1 pH increments, assuming the ionized, or non-ionized structures at the respective pKa (calculated as implemented in Discovery Studio software, from Accelerys Inc.) of the side chains and interpolating the charges along the pH scale according to acid-base equilibrium relationships. The charge-related molecular surface properties have been calculated for each tested protein at its isoelectric point. The values of the calculated charges for each amino acid versus pH, as well as a detailed explanation of their calculation, are presented in File S4 and File S5.

### Hydrophobicity

Among the many hydrophobicity scales proposed in the literature, the present analysis used the hydrophobicity as defined by Wimley and White [65]. Briefly, the hydrophobic character, or lack of, of an amino acid, is estimated by the enthalpy of the transfer of a peptide through a lipid membrane (ΔG_wm_), calculated from the thermodynamic measurements of the actual transfer of model penta-peptides that have embedded the amino acid of interest. Both hydrophobicity and hydrophilicity have been calculated, and the protein amphiphilicity is the algebraic sum of hydrophobicity, expressed in negative numbers, and hydrophilicity, expressed in positive numbers.

### Molecular surfaces

Because of its conceptual benefits, i.e., the virtual probing of the molecular surfaces mimics the actual contact between the protein and a real object, the original Connolly’s algorithm [39]-[41] has been used to construct protein molecular surfaces. The algorithm has been upgraded to record the geometry of the molecular surface, protein amphiphilicity, hydrophobicity, hydrophilicity and charges, both positive and negative. Briefly, the algorithm records the position of the points of contact between a virtual rolling probing ball with a set radius and the atoms on the molecular surface of the protein, or alternatively the points placed at a distance equivalent to the van der Waals radius of the respective atoms.

The spatial distribution of the amphilicity, hydrophobicity and hydrophilicity on the protein molecular surface was determined through the allocation, at the point of contact, of the value for the respective amino acid, weighted with the ratio of the probed surface per the total area of the amino acid. A similar procedure was used for mapping the spatial distribution of the charges. The procedure involves the allocation of the specific charge weighted with the ratio between the probed atomic area and the total atomic area. The procedure is similar to the one used by Scarsi et al. [60], with the difference that only the actual property is recorded, instead of the interaction with the probe and that the charges are also accounted for.

The probing of the protein molecular surface was performed with probe with increasing radii, from 1.4 Å to 20 Å, because beyond a certain value of the radii the variation of the properties is negligible; and because, for flat solid surfaces, the actual real solid radius of an engineering grade-flat solid surface is close to this value [66]. The increase of the probe radii results in a large ratio of the area created due to the re-entry points of the probe and the overall molecular surface. Because our analysis uses the quantification of physico-chemical properties on the molecular surface at the points of contact, we used only the contact area in our calculations and graphical representation.

The calculations were run using Connolly’s original software code [39] upgraded for the quantification of physico-chemical properties and with a Windows Graphical User Interface. The 4D points (the x,y,z coordinates and the molecular property) were visualized using DS Viewer Pro (from Accelerys Inc.).

### Protein properties on the molecular surface

The characterization of the protein molecular surface requires the quantification of several properties on the molecular surface: (i) *global properties*, namely, total surface, total charges and total amphiphilicity, hydrophobicity and hydrophilicity, as well as the area-per-volume ratio; (ii) *property surface densities,* namely, charge, amphiphilic, hydrophobic and hydrophilic density, calculated by dividing the respective total property to the total biomolecular area; and (iii) *property specific surface densities,* calculated as in (ii), but dividing the respective property, e.g. positive charge, to the area that property turns up, e.g., positive charged area. A synthetic view of all parameters is presented in Table 2.

**Table 2:** Definition of the properties measured on the protein molecular surface.

No.		Property	Symbol	Method of calculation	Units
**1. Global properties**					
1.1	Molecular weight		Mw	PDB	Da
1.2	Total number of atoms		Na	PDB	-
1.3	Total number of residues		Nr	PDB	-
1.4	Total probed area		A	Connolly	Å^2^
1.5	Volume		V	Eq. 2	Å^3^
1.6	Shape factor		V/A	Eq. 2	Å
1.7	Total positive charge		PC_t	Connolly upgrade	e
1.8	Total negative charge		NC_t	Connolly upgrade	e
1.9	Total hydrophilicity		Phi_t	Connolly upgrade	kcal mol^-1^
1.10	Total hydrophobicity		Pho_t	Connolly upgrade	kcal mol^-1^
1.11	Total positively charged area		A_pc	Connolly upgrade	Å^2^
1.12	Total negatively charged area		A_nc	Connolly upgrade	Å^2^
1.13	Total hydrophilic area		A_phi	Connolly upgrade	Å^2^
1.14	Total hydrophobic area		A_pho	Connolly upgrade	Å^2^
**2. Property surface densities**					
2.1	Positive charge density		PC_d	PC_t/A	e Å-^2^
2.2	Negative charge density		NC_d	NC_t/A	e Å-^2^
2.3	Hydrophilic density		Phi_d	Phi_t/A	kcal mol^-1^Å-^2^
2.4	Hydrophobic density		Pho_d	Pho_t/A	kcal mol^-1^Å-^2^
**3. Property specific densities**					
3.1	Positive charge specific density		PC_sd	PC_t/A_pc	e Å-^2^
3.2	Negative charge specific density		NC_sd	NC_t/A_nc	e Å-^2^
3.3	Hydrophilic specific density		Phi_sd	Phi_t/A_phi	kcal mol^-1^Å-^2^
3.4	Hydrophobic specific density		Pho_sd	Pho_t/A_pho	kcal mol^-1^Å-^2^

### Statistical correlation between molecular surface properties and protein interfacial processes

The statistical strength of the correlation between the protein surface concentration on various solid surfaces and the respective protein physico-chemical parameters was firstly estimated by the Pearson Product-Moment Correlation Coefficient (PPMCC), as implemented in the Statistica software, from StatSoft Inc. The protein parameters taken into consideration, calculated on the protein molecular surface, as well as comprising the totality of the residues, were amphiphilicity, hydrophobicity and hydrophilicity, and their derived surface densities.

The PPMCC calculation was applied to a reduced set of proteins out of the initial set of 35, i.e., the five model proteins mentioned above plus α lactalbumin and β lactoglobulin (in italics in Table 1) for which comprehensive data regarding protein adsorption could be found in the Biomolecular Adsorption Database (BAD)[25], totaling 279 valid data points. PPMCC was calculated for all data points, and separately for hydrophilic and hydrophobic solid surfaces. The amphiphilicity of solid surfaces is usually quantified by the contact angle of a small (1µl) water droplet, which is the angle made by the intersection of the contour of the gas/liquid interface with the solid surface. While in general solid surfaces are considered hydrophobic if exhibit contact angles above 90°, in the particular case of protein adsorption the adsorbing solid surfaces are hydrophobic for contact angles above 45°,[25] with those below considered hydrophilic. With this threshold, the protein adsorption data for hydrophilic solid surfaces comprises 172 data points and for hydrophobic solid surfaces comprise 107 data points.

A piecewise, multilinear regression procedure with breakpoint, reported before [25], was applied to all data points for both hydrophobic and hydrophilic solid surfaces, as well as separately for the two subsets, i.e., hydrophobic and hydrophilic solid surfaces, respectively. The regression provided a measure of the correlation between the output variable, i.e., protein concentration on adsorbing solid surfaces; and sets of input variables comprising (i) protein concentration in solution; (ii) solid surface amphiphilicity measured by the respective contact angle; (iii) buffer parameters, i.e., pH, ionic strength; (iv) global bulk parameters of the protein, i.e., isoelectric point, molecular weight; (v) global molecular surface of the protein, i.e., molecular area, surface-to-volume ratio; (vi) hydrophobicity parameters derived from the probing of the molecular surface, i.e., hydrophobicity density, hydrophobicity specific density, and ratio between hydrophobicity and hydrophilicity, all derived for different probing radii; and (vii) charge parameters derived from the probing the molecular surface, i.e., positive charge density, positive charge specific density, and ratio between positive and negative charge, all derived for different probing radii.

## Results and Discussion

### Areas and surface-to-volume ratio of the protein

**Figure 1:**
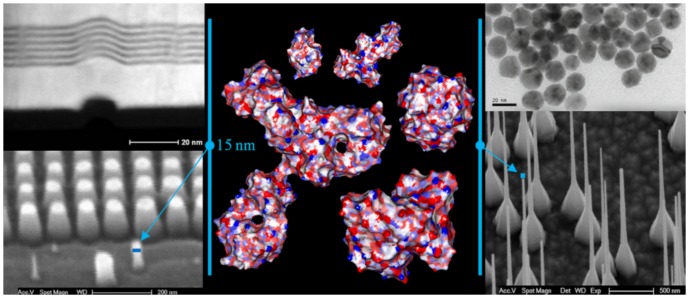
Molecular surface of several proteins (middle panel) and several artificial nano-surfaces and objects. The amphiphilic (blue – hydrophilic; red - hydrophobic) molecular surface is mapped at 1.4 Å (water molecule dimensions) geometrical resolution, for five proteins, from left to right and top to bottom: lysozyme, ribonuclease; hemoglobin; IgG and albumin. The artificial surfaces are as follows: Top left: TEM image (side view) of a defect propagating from layer to layer in otherwise perfectly flat Pt/Rh multi-layered surface; feature: 50×100 Å. Top right: TEM image of gold nanoparticles; features: 10–25 Å. Bottom left: SEM image of a set of SiO_2_ pillars with gold caps; features: 150 – 300 Å. Bottom right: SEM image of SiO_2_ nano-wires grown from vapor phase; minimum feature: sub-500 Å.

Many biomolecules, in particular proteins, are similar in size with the nanostructures present on artificial or natural surfaces, or with nano-objects, e.g., nanoparticles. Figure 1 represents a brief comparison between the molecular surface of five proteins with different sizes, with several examples of artificial nano-objects, either ‘flat’ solid surfaces or particles. The following discussion will focus on five representative proteins, i.e., lysozyme, ribonuclease, hemoglobin, albumin and IgG, which have very different molecular weights, i.e., from 129 to 1344 residues (Table 1, in bold); and shapes, i.e., globular, ellipsoidal and Y-shaped.

**Figure 2:**
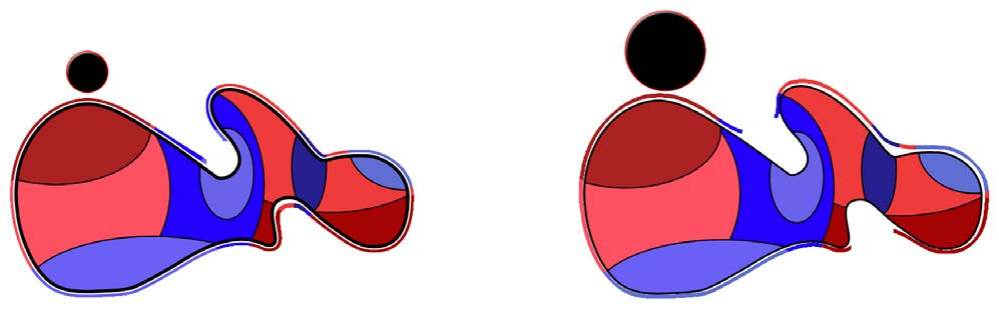
Schematic of different molecular surfaces obtained when probing a protein with different probe radii. Larger probes (right) cannot visit some inner areas of the protein, as well as some parts of the residues.

As it can be easily inferred from Figure 1, and as in a classical fractality problem, the shape and extent of the molecular surface area depend, on one hand, on the characteristics of the molecule, e.g., structure, number of atoms; and, on the other, on the radius of the probing ball, be that virtual or real. Figure 2 presents a schematic view of the evolution of the constructed molecular surface as a function of the probe radius.

The probing of the molecular surface creates non-contiguous surfaces, especially at large radii, as explained in Figure 2. Alternatively, holes can be the result of the actual structure of the protein, independently of the size of the probe radius, as presented in Figure 1.

**Figure 3:**
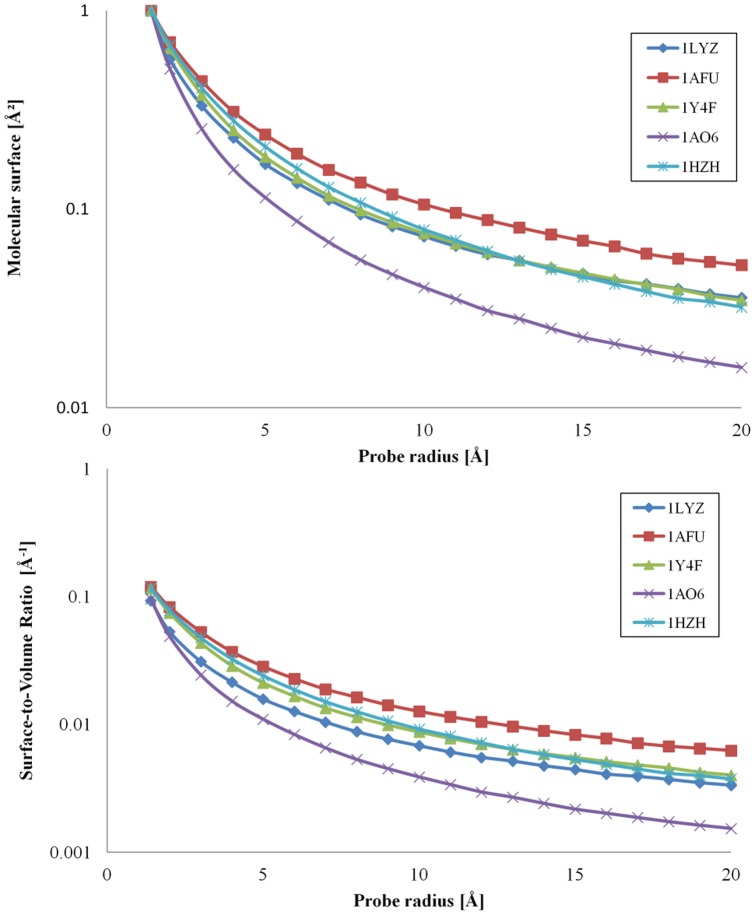
Variation of the molecular surface area and surface-to-volume ratio with the radius of the probe. Molecular surface area (top) and surface-to-volume (bottom) for five model proteins: 1LZY  =  lysozyme; 1AFU  =  ribonuclease-A; 1Y4F  =  human hemoglobin; 1AO6  =  human serum albumin; and 1HZH  =  intact human IgG.

Figure 3 presents the quantification of the area of the molecular surface and the surface-to-volume ratio for different probe radii for the five chosen representative proteins, i.e., lysozyme (1LYZ), ribonuclease (1AFU), hemoglobin (1Y4F), albumin (1AO6) and IgG (1HZH). These proteins have vastly different molecular weights, i.e., from 129 to 1344 residues (Table 1, in bold).

The probed area decreases monotonically with the increase of the probe radius, but after a certain radius value, which depends on the size of the protein, it reaches a plateau. The relative decrease of the probed area is more pronounced for large proteins. For instance, at the plateau, the total area decreases to about 40% and 25%, for IgG (1HZH) and for albumin (1AO6), respectively, compared to their area obtained through the contact with a probe with a 1.4Å radius. In the first approximation, the probe radius after which the protein probed area does not vary substantially is slightly larger than the largest distance between two atoms of the respective protein, i.e., approximately 60 Å and 150 Å, for 40 Å-large lysozyme (1LYZ) and 100-120 Å-large IgG (1HZH) or albumin (1AO6), respectively. Most proteins larger than 50kDa usually form two or more domains independently folded [67]. The evolution from single to several globular domains with the increase of the molecular weight of the protein leads to an increase in the roughness of the molecular surface, rather than the change of its overall shape.[68] There are however proteins (not considered here) which exhibit highly elongated shapes, e.g., fibrinogen, but other very large proteins present specialized structures such as coiled coils, e.g., myosin with its very convoluted (and dynamic) shape[69] or the collagen triple helix.[67] Therefore, the difference in the evolution of the decrease of the probed area versus probe radius for proteins with different sizes appears to be the result of either the increased roughness of the molecular surface, or the departure from the globular shape (e.g., for IgG).

For the set of proteins studied here, the *molecular surface area* can be estimated with very good statistical quality (R^2^ = 0.98) as a function of its molecular weight (or number of atoms) and the probe radius, as follows:
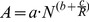
(1)


where A is the probed area on the protein molecular surface (Å^2^); N is the number of atoms in the protein; R is the probe radius (Å); and a, b and c are fitting constants, which have values, for the set of 35 proteins considered, of a  =  4.36; b  =  0.95; c  =  0.165. This relationship (Eq. 1) predicts that for a very large probe radius, i.e., R→∞, which is equivalent to a flat surface, the protein probed area is nearly proportional (i.e., c/R→0; 0.95<b<1) with the number of atoms in the protein, or by extension, to its molecular weight. In reality, engineering-grade-flat solid surfaces exhibit nanometer-range roughness, e.g., with features of around 20 Å[66].

#### Surface-to-volume ratio

Because the probing of the molecular surface, when performed with probes with large radii, generates non-contiguous molecular surfaces, the actual volume of the protein has to be calculated as a sum of the volumes of the constituent atoms, rather than the volume inside the molecular surface. Consequently, the molecular surface-area-to-volume ratio, or simply the *surface-to-volume ratio* is given by
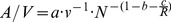
(2)


where V is the volume of the protein, proportional with the number of atoms, N; v is the average atomic volume of the protein atoms[67]; and a, b and c are the constants in Eq. 1. A consequence of Eq. 2 is that the surface-to-volume ratio of larger proteins will decrease more with the increase of the probe radius than that of smaller proteins. Figure 3 (bottom) presents the evolution of the surface-to-volume versus the probe radius for five representative model proteins.

**Figure 4:**
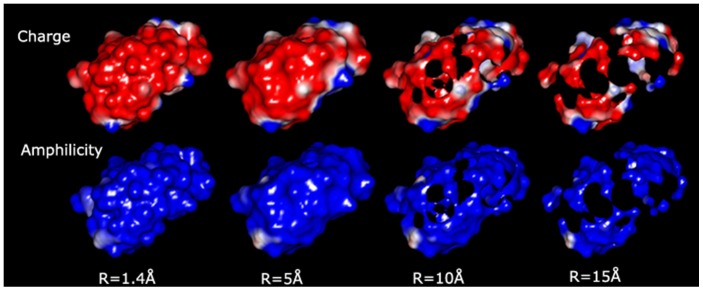
Physico-chemical properties of ribonuclease represented on its molecular surface. The probing is performed with increasingly large probe radius (from left to right), as charges (top row, red  =  negative, blue  =  positive), and amphiphilicity (bottom row; blue  =  hydrophilic; red  =  hydrophilic).

The graphical representation of the molecular surface (Figure 1 and an example for ribonuclease in Figure 4) allows for some qualitative considerations. For simple, globular, small-to-medium proteins, e.g., lysozyme (1LYZ), ribonuclease (1AFU), the use of small radii, e.g., 1.4 Å, results in contiguous molecular surfaces with distinct negative and positive patches (red and blue, respectively, in Figure 1, middle top). Conversely, for large proteins with more complex shapes, e.g., IgG (1HZH), (Figure 1, middle left) the probing with small probes will result in non-contiguous molecular surfaces. Eventually, the use of probes with larger radii results in non-contiguous molecular surfaces even for smaller proteins, e.g., ribonuclease (Figure 4), and the subsequent decrease of the exposed area.

#### Significance

The above results and discussion lead to the following partial conclusions.

- Above a certain roughness of the solid surface that interacts with a protein, i.e., in the range of 10–20 Å, the contact area of the protein with the solid surface, which is proportional with the molecular weight of the protein, remains constant versus the probe radius. Consequently, for applications seeking the amplification of the protein-solid surface interactions, assumed to be proportional with the contact area between the protein and the solid surface, e.g., liquid chromatography, and notwithstanding the flexibility of the protein (to be briefly discussed later), negligible effects are expected for a solid surface roughness above 10 Å for small globular proteins and 20 Å for large proteins.
- The contact area represents only a fraction of the total protein molecular surface and this fraction is much smaller for large probe radii. As hydrophobic amino acids have the propensity to aggregate towards the center of the protein[70] , it follows that the contact area of the probe, be that virtual or real, with the “hydrophobic core” will decrease with the increase of the probe radii. Consequently, very flat *hydrophobic* solid surfaces are expected to be inefficient for hydrophobicity-controlled protein immobilization; or, alternatively, they will induce important conformational changes of the proteins if the hydrophobic solid surface reaches the contact with the hydrophobic amino acids localized inside the protein core.

### Amphiphilicity and charge on the molecular surface

#### Amphiphilicity and charges on the molecular surfaces

A full portrayal of the protein molecular surface entails both the geometrical position of the points of contact and the description of the physico-chemical parameters at those positions. Qualitative, yet insightful observations can be gathered from the inspection of the representation of the charges and amphiphilicity on the protein molecular surfaces, as a function of the probe radius, such as presented for ribonuclease in Figure 4. The charged molecular surface comprises largely, but not exclusively, negative charges, due to the more exposed oxygen atoms in carboxy groups. This propensity decreases, relatively, with the increase of the radius of the probe (Figure 4, top row), as the contiguous negative charged molecular surface is ruptured due to the impossibility of larger probes to reach the negatively charged regions towards the core, e.g., negative oxygen atoms in amide groups; while the positively charged areas of the few amino groups placed away from the protein core remain largely unchanged. Conversely, the molecular surfaces (Figure 4, bottom row) remain largely, and evenly, hydrophilic with the increase of the probe radius, as the probe will touch atoms with amphilicities assigned according to their parent amino acid, which those that are hydrophilic predominantly placed away from the protein core.

#### Hydrophobic and negatively charged areas

**Figure 5:**
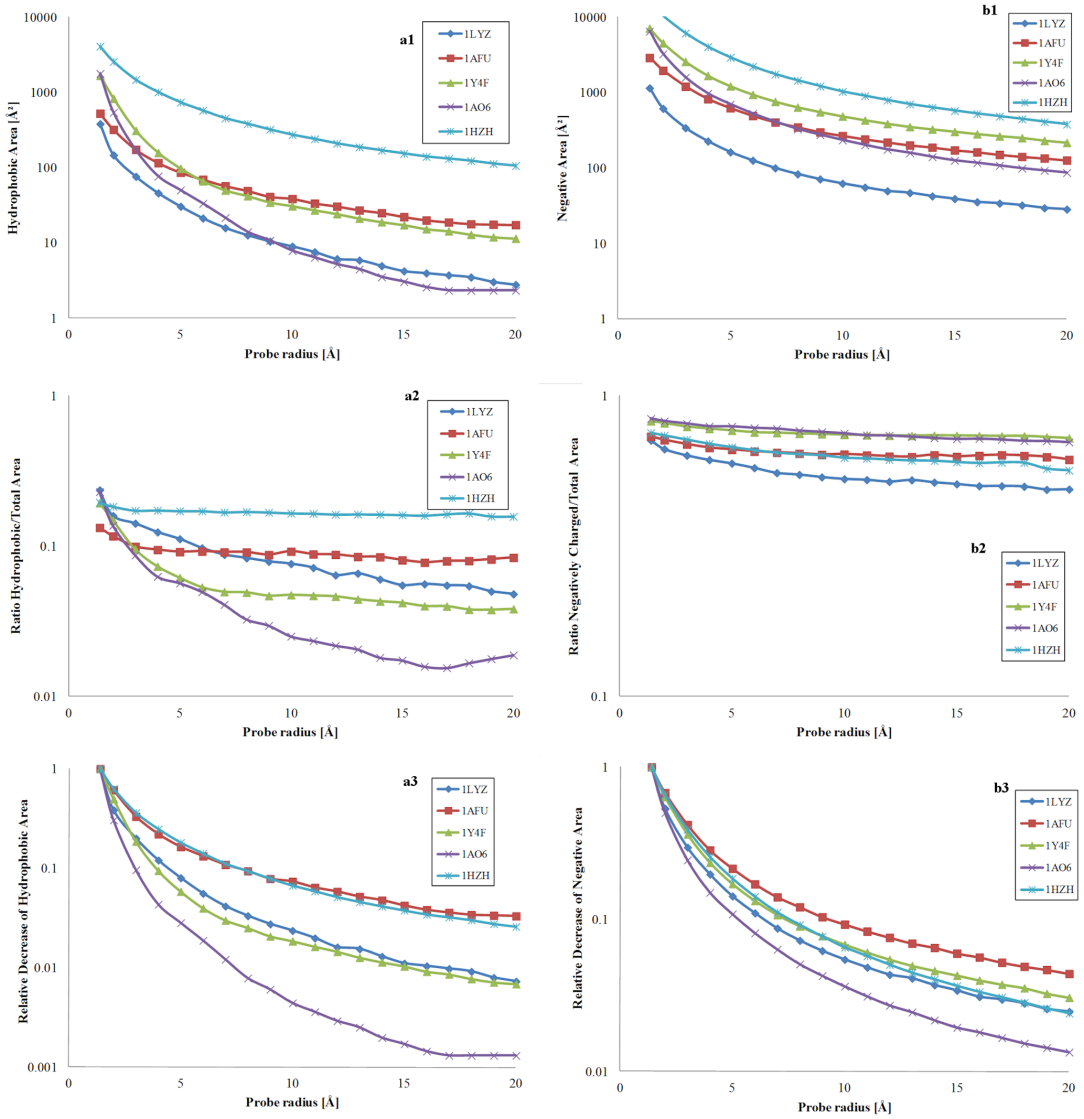
Hydrophobicity-related areas; and negatively charged-related areas modulated by the probe radius. Left: hydrophobic area (a1, top); ratio of hydrophobic per total area (a2, middle); and relative decrease of the hydrophobic area, reported to its maximum extent at minimum probe radius, (a3, bottom) for five model proteins: 1LZY  =  lysozyme; 1AFU  =  ribonuclease-A; 1Y4F  =  human hemoglobin; 1AO6  =  human serum albumin; and 1HZH  =  intact human IgG. Right: negatively charged area (b1, top); ratio of negatively charged per total area (b2, middle); and relative decrease of the negatively charged area, reported to its maximum extent at minimum probe radius (b3, bottom) for the same model proteins.

This qualitative description is supported by quantitative data, presented in Figure 5 for the five model proteins discussed before. The full account of the calculations is presented in File S1.

The overall hydrophobic and the negatively charged areas (Figure 5a1 and 5b1, respectively; logarithmic scales) decrease, as expected, with the increase of the probe radius, similarly with the decrease of the overall area (Figure 3 top). Interestingly, the ratio of hydrophobic-to-total area (Figure 5a2; logarithmic scale) remains nearly constant for IgG, and, to a lesser extent, for ribonuclease and hemoglobin, with albumin and lysozyme decreasing constantly. Conversely, the ratio of negatively charged-to-total area (Figure 5b2) presents a monotonic decrease, and in a much tighter range than hydrophobicity. Finally, both the ratio of the hydrophobic and negatively charged areas divided to their maximum value (at 1.4Å) with the increase of the probe radius, as presented in Figures 5a3 and 5b3, respectively, present a monotonic decrease, with the evolution of charges more protein-specific than that of hydrophobicity.

#### Amphiphilic, hydrophobic, hydrophilic and charge densities

**Figure 6:**
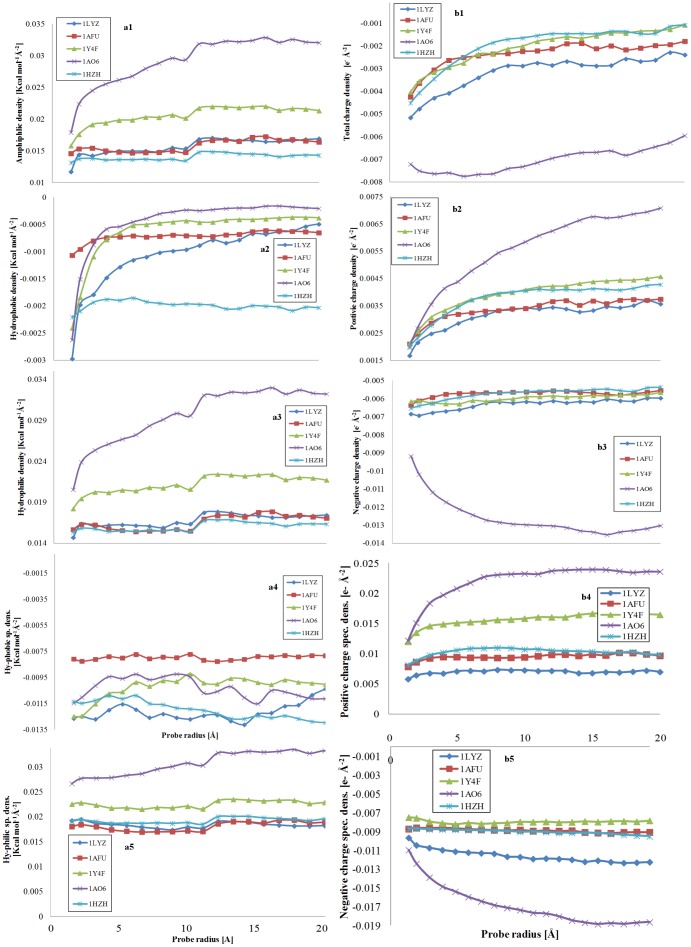
Amphiphilic, hydrophobic and hydrophilic densities; and total, positive and negative densities modulated by the probe radius. Left: Impact of the probe radius on the amphiphilic (a1, top), hydrophobic (a2, second from the top) and hydrophilic (a3, third from the top) densities, i.e., reported to the total area of the protein; and of the hydrophobic (a4, forth from the top) and hydrophilic (a5, fifth from the top) specific densities, i.e., reported to their respective areas for five model proteins: 1LZY  =  lysozyme; 1AFU  =  ribonuclease-A; 1Y4F  =  human hemoglobin; 1AO6  =  human serum albumin; and 1HZH  =  intact human IgG. Right: Impact of the probe radius on the total (b1, top), positive (b2, second from top), and negative (b3, third from top) densities, reported to the total area of the protein; and of positive (b4, forth from top), and negative (b5, fifth from top) specific density, reported to their respective areas for the same five model proteins.

The variation of the densities of the physico-chemical properties, i.e., amphiphilicity, hydrophobicity, hydrophilicity; and total, negative and positive charges, with the probe radius (Figure 6) provides a finer protein-specific analysis.

In general, the amphiphilic density (Figure 6a1) is increasing, mildly, with the increase of the probe radius, after a threshold around 3Å, which is equivalent to more hydrophilic areas being exposed by larger probes compared to smaller ones. While following this general trend, albumin (1AO6) presents however a large increase of the amphiphilic density with the probe radius, which could explain the very good blocking, protein-repelling properties of this protein. The evolution of the hydrophobic density with the probe radius (Figure 6a2) is more protein-specific. Indeed, the hydrophobic density of IgG (1HZH) and ribonuclease (1AFU) remain constant, the hydrophobic density of albumin (1AO6) and hemoglobin (1Y4F) decrease steeply until approximately 3Å after which remain constant, and finally the hydrophobic density of lysozyme presents a monotonic decrease with the probe radius. Interestingly, the hydrophobic density of IgG (1HZH) remains four times higher than that of all other proteins, thus explaining its propensity for adsorption on solid surfaces. Because it represents a large proportion of the overall amphiphilicity, the evolution of the hydrophilic density (Figure 6a3) presents similarities with that of amphiphilic densities. These findings indicate that the ‘hydrophobic core’ concept is in general valid, as inferred from the increase of the amphiphilic (and hydrophilic) density with the increase of the probe radius. The level of protection of the hydrophobic core from the exposure to probes as a function of their size varies largely from protein to protein: (i) small, globular proteins are gradually exposing less hydrophobic regions to gradually larger probes; (ii) large proteins with pseudo-globular shapes stop becoming less hydrophobic for probe diameters similar to the protein size; and (iii) large protein with non-globular shapes present low level of protection of the hydrophobic core.

In contrast with the evolution of amphiphilic density (Figure 6a1), the total charge density (Figure 6b1) is decreasing monotonically towards zero with the increase of the probe radius, , which is equivalent to less *overall* charged areas being visited by large probes than by small ones. While it would be expected that an increase of the amphiphilic density would by linked to an increase in the charging of the respective areas, this apparent contradiction can be understood observing separately the evolution of the constitutive elements, i.e., the positive and negative charge density.

Indeed, the positive charge densities (Figure 6b2), presents a similar evolution with the hydrophilic density, i.e., a gradual increase of positive charge density with the size of the probe radius. On the other hand, with one exception (albumin) the evolution of the negative charge density is rather uneventful, with only very small decreases of the density. As it was the case before, albumin presents an exceptional increase of *both* positive and negative charge density, which can explain its exceptional evolution with regard to hydrophilic density. The evolution of the property *specific* density, i.e., the hydrophobic, hydrophilic, positive and negative charge values quantified on the molecular surfaces, then divided by their specific areas, offer a different perspective into the variation of physico-chemical properties on the protein molecular surfaces. With few notable exceptions, the hydrophobic and hydrophilic specific densities (Figure 6a4 and Figure 6a5) do not vary substantially. However, mirroring the evolution of the respective densities, IgG (1HZH) presents an increase, albeit moderate, of its hydrophobic specific density; and albumin (1AO6) presents a considerable increase of its hydrophilic specific density. The positive and negative charge specific density (Figure 6b4 and 6b5) replicate the evolution of their overall counterparts, including the exceptional behavior of albumin.

#### Molecular surfaces of single-point mutants

**Figure 7:**
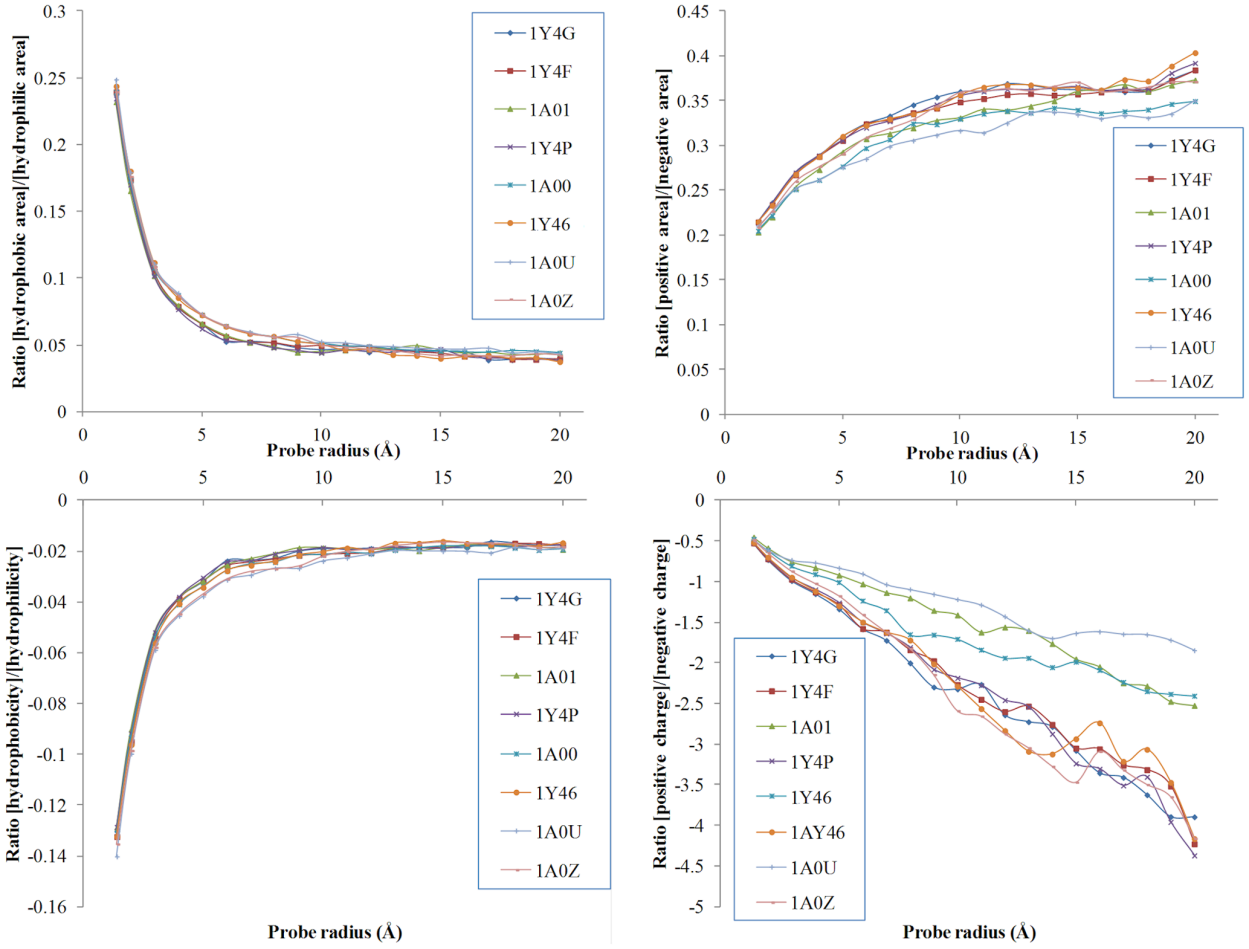
Ratio of the molecular surface properties for single-point mutants (haemoglobin structures) as a function of the probe radius. The top panels represent the ration between the hydrophobic and positive areas, respectively, reported to the hydrophilic and negative areas, respectively. The bottom panels represent the ratios of the respective properties. The members of the sub-set are as follows: 1Y4F (W37A); 1A01 (W37A); 1Y4P (W37E); 1A00 (W37Y); 1Y46 (W37Y); 1Y4G (W37G); 1A0U (V1M); and 1A0Z (V1M).

The high-resolution X-ray structures of the deoxy forms of four recombinant hemoglobins in which a Trp residue has been replaced with Tyr, Ala, Glu, or Gly, have been reported[64] and recently the structures have further refined.[71] As it was found that no significant mutation-induced changes in tertiary structure were detected, we used this restricted sub-dataset to test the fine definition of the molecular surface for very similar proteins. The evolution of the hydrophobicity and charges on the respective molecular surfaces modulated by the variation of the probing radius is represented in Figure 7 as ratios between various properties. Interestingly, the evolution of the ratios of hydrophobic/hydrophilic areas and hydrophobicity/hydrophilicity are essentially indistinguishable for the hemoglobin structures considered. In contrast, the ratios of positive/negative areas and that positive/negative charges are quite different from one hemoglobin structure to another, especially for the latter. This difference between the hydrophobicity- and charge-based ratios suggests that atom-based properties have the potential of describing more specifically the molecular surface than amino acid-based properties.

#### Significance

The partial conclusions flowing from the analysis of the impact of the probe radius on the molecular surface properties are as follows:

- While the present analysis shows that the “hydrophobic core” model stands valid for the proteins studied, in many cases this concept requires serious qualifications, as proteins appear to be specific regarding the propensity for protecting their hydrophobic core. In this context, a recent contribution[72] dealing with the quantification of the shape and distribution of the hydrophobicity of disordered proteins, which play a significant role in many biological processes, showed the lack of a well-formed hydrophobic core unlike that of the globular proteins.
- Similarly with the “hydrophobic core” concept, it appears that proteins have a “positive charge core”. This positive charge core is however less evident, and it is arguably of a lesser importance than the hydrophobic core, due to the long range of electrostatic interactions, compared with the short range hydrophobic ones.
- Although it is expected that the amphiphilicity and the charges of the protein on their molecular surface are correlated, e.g., a higher charged area will be more hydrophilic, these two sets of parameters are specific enough to deny a univocal relationship between them.
- An atom-level description of the amphiphilicity, proposed recently[73] would allow arguably a more precise treatment of molecular surface, and even open the possibility of deriving “hydrophobic potentials”, as proposed before, e.g.,[74], similarly with electrostatic potential (but with very different mathematical formalism).
- The probing of the protein with a large ball *in silico* is conceptually the closest to the interaction between a protein and a real nanoparticle. This conceptual commonality could open ways of designing nanoparticles that are tailored to elicit a desired response from the protein:nanoparticle complex, as it was proposed recently [26], thus turning the phenomenon of protein corona from a deleterious effect into a powerful nanoengineering tool. Indeed, while the concept of hydrophobic core and the existence of hydrophobic patches is well established, much less attention has been paid to the distribution of hydrophobic-complementary (or charge-complementary) “patches” on nano-surfaces. Because the proteins are actually not as flexible[67] as usually thought, the design of hydrophobic- and/or charge complementary nanosurfaces is conceivable, and possibly achievable.

### Correlation between molecular surface properties and protein adsorption

#### Protein adsorption

The quantification of the physico-chemical properties, in particular amphiphilicity, on the protein molecular surface raises the expectation that these parameters could be correlated with measures of the interaction between a protein and a solid surface, e.g., protein adsorption on solid surfaces, protein interaction with lipid membranes, protein aggregation in large fibrilar structures. Among these, protein adsorption is arguably the best documented. Recently, data regarding the protein adsorption published in the open literature in the last half a century have been archived in a Biomolecular Adsorption Database [25], which register the amount of protein adsorbed on a particular solid surface, the structural properties of that protein (most relevant for this study, the structure deposited in the PDB database and component residues), as well as the solid surface contact angles, properties of the fluid media, method of measurement, etc.

#### Statistical strength of the correlation between molecular surface parameters and protein adsorption

**Figure 8:**
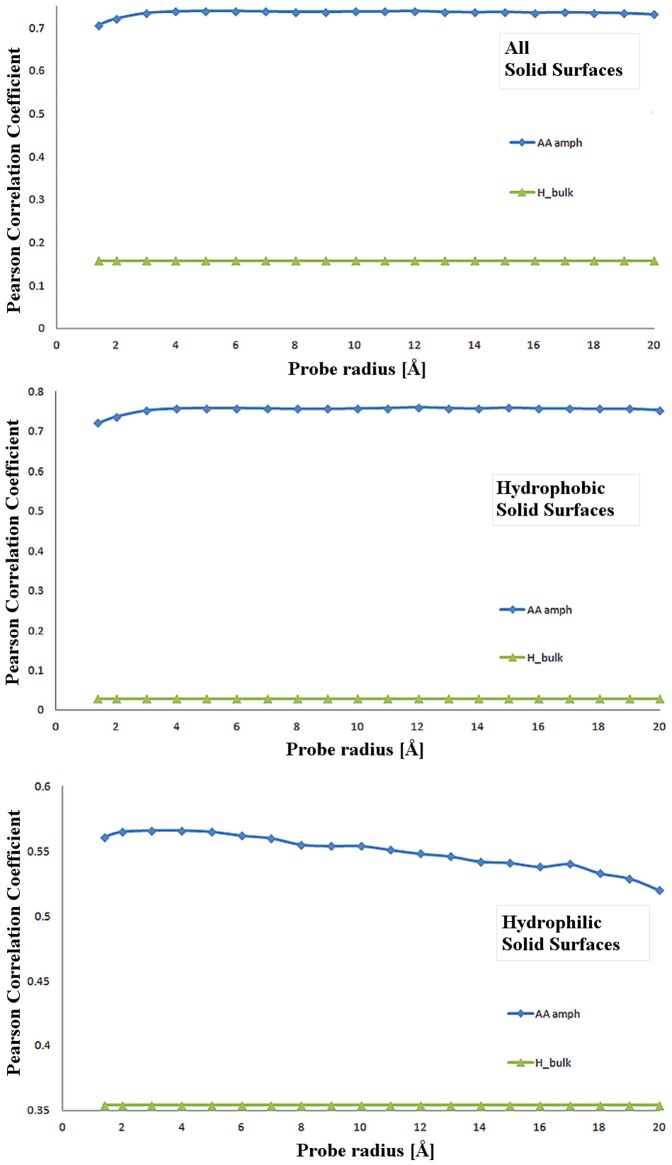
Pearson Product-Moment Correlation Coefficient (PPMCC) of the relationship between protein parameters the protein adsorbed mass. Protein properties are bulk amphiphilicity (H_bulk), and amphiphilicity (AA_amph). The adsorbed mass of the respective proteins (as reported in Biomolecular Adsorption Database [25]). PPMCC calculations are presented for all surfaces (top); hydrophobic (i.e., contact angle > 45°middle); and hydrophilic (i.e., contact angle < 45°bottom).

The Pearson Product-Moment Correlation Coefficient (PPMCC) represents the strength of a statistical *linear* correlation between two variables, with 1, or -1, the former for both variables increasing, representing perfectly linear correlations. In the context of this study, the closer the PPMCC value is to 1 (or -1), the higher the predictive power is for the protein physico-chemical parameter assumed as predictor of protein adsorbed mass on a solid surface. The evolution of PPMCC with the probe radius (Figure 8) demonstrates that the molecular surface-based properties, such as amphiphilicity, hydrophobicity and hydrophilicity, are vastly better predictors than the same properties calculated from all residues, exposed or not to the molecular surface. More specifically, the results reveal specific features of different physico-chemical parameters, as follows.

- For *all solid surfaces* (Figure 8, top) the molecular surface-based amphiphilicity presents a PPMCC around 0.7–0.8, compared with the PPMCC of the ‘bulk’ amphiphilicity, which has a small (0.16) constant value irrespective of the probe radius. This high statistical strength is remarkable, having in mind the extreme spread of the protein and solid surface properties, and experimental data (buffers, methods of measurement, etc.), as well as the fact that PPMCC assumes a linear relationship between the tested parameters, while protein adsorption is clearly a non-linear phenomenon.
- For *hydrophobic solid surfaces* (Figure 8, middle) the statistical relevance of molecular surface amphiphilicity is high, in the region of ±0.7, and decreasing only slightly with the probe radius. The ‘bulk’ counterpart, with PPMCC values close to 0, has no statistical relevance. The slight decrease of the amphiphilicity-related PPMCC can be understood in the context of the tug-of-war between (i) protein molecular surface hydrophobicity, which increases the propensity for its adsorption on hydrophobic solid surfaces; and (ii) protein molecular surface hydrophilicity which increases the protein solubility, and consequently allows for higher concentrations of protein in solution, which in turn increases protein adsorption.
- For *hydrophilic solid surfaces*, (Figure 8, bottom) the statistical relevance of molecular surface-derived amphiphilicity is lower than for hydrophobic solid surfaces, albeit still substantial (0.5–0.6). This decrease of the PPMCC is the result of the protein adsorption on hydrophilic solid surfaces being governed to a lesser extent by hydrophobic interactions.

#### Multilinear regressions of the correlation between molecular surface parameters and protein adsorption

**Figure 9:**
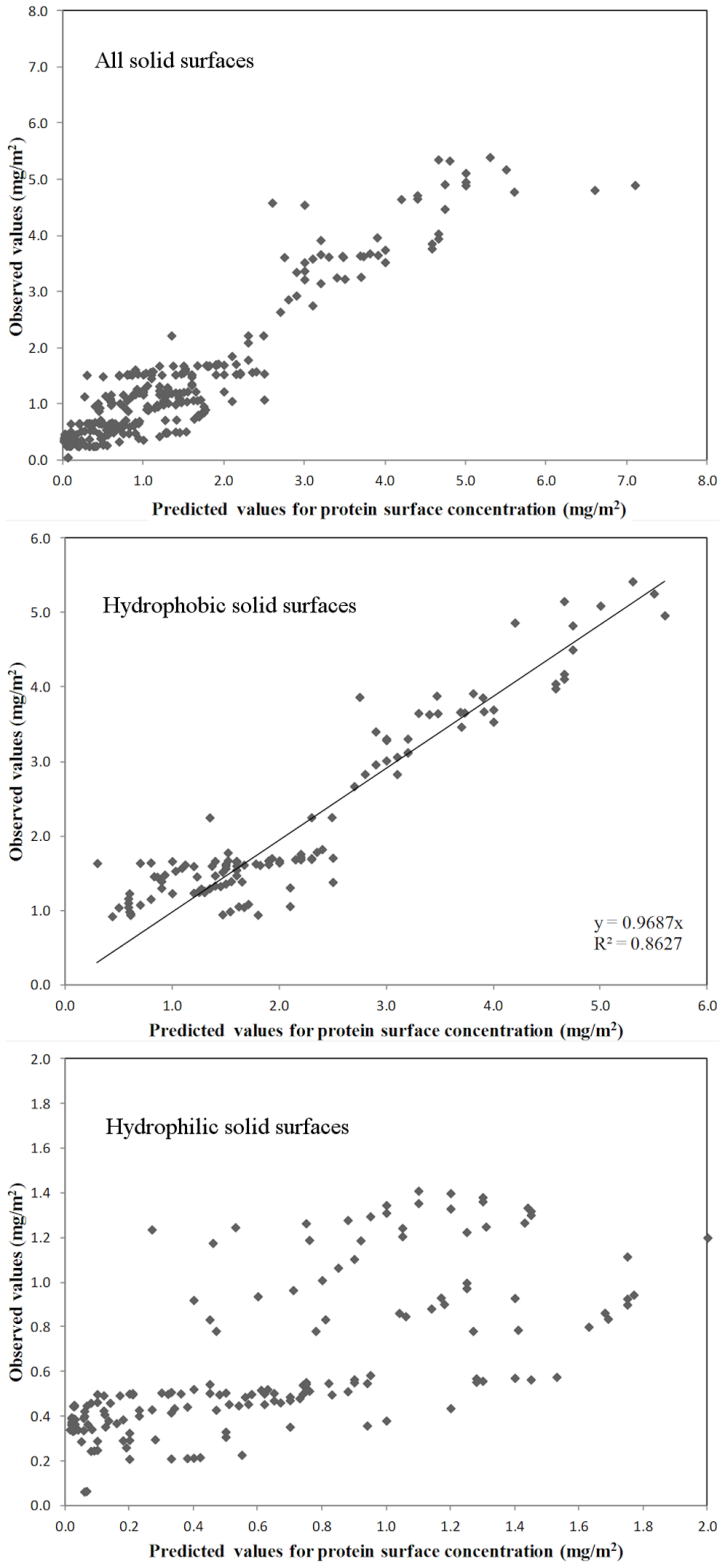
Statistical strength of the piecewise linear regression between molecular surface properties and concentration of protein on adsorbing surfaces measured as the fit between observed vs. predicted data. Comparison presented for all surfaces, i.e., hydrophobic and hydrophilic surfaces (top); hydrophobic surfaces only (middle) and hydrophilic surfaces (bottom). The fitted line in the middle panel represents the linear regression between predicted and observed data, forced to pass through origin, and not the actual multilinear regression with breakpoint best fit

The results of the piecewise linear regression that connects the molecular surface properties, calculated for the maximum probing radius (20 Å) and adsorbing solid surface properties (Figure 9) demonstrates more compellingly the statistical relevance of this relationship, in particular regarding solid surface hydrophobicity.

The quasi-nonlinear feature of the piecewise linear regression, i.e., one multilinear relationship until a set breakpoint, followed by a different linear relationship after, succeeds in fitting well all available data, (Figure 9 top) for both hydrophobic and hydrophilic solid surfaces, and for a large span of protein concentrations on the adsorbing solid surfaces, i.e., close to 8 mg/m^2^. However, while the overall regression coefficient is reasonably high, i.e., R^2^  =  0.8758, a closer inspection of the comparison of predicted vs. observed data reveals that the fit starts to lose its quality for higher concentrations on the solid surface, e.g., higher than 4 mg/m^2^. This loss of quality can be due to the fact that, at high surface concentrations, i.e., higher than those required for a complete coverage of the surface, the correlation between protein and adsorbing solid surface properties loses its physical meaning, because the protein does not interact with the solid surface, but with other proteins immobilized on the solid surface. It is also interesting to note that including in the analysis the charge-related properties does not bring any improvement in the statistical quality of the fit.

The piecewise linear regression performed for hydrophobic solid surfaces results in much better overall fit, as suggested by Figure 9 middle panel, despite a slight decrease of the correlation factor, possibly due to the smaller pool of data. As before, the addition, or deletion of charge-related variables is rather inconsequential for the quality of the statistical fit.

Finally, the linear regression performed for the sub-set of hydrophilic solid surfaces (Figure 9 bottom) has the poorest quality, due to the lower relevance of hydrophobicity-related properties for protein adsorption on hydrophilic solid surfaces.

#### Significance

Following the establishing of the statistical relevance of amphiphilicity quantified on the protein molecular surface, this could be used further for finding relationships between the protein parameters, and those of the solid surfaces the proteins interact with, on one side; and the result of the interaction, on the other side. For instance, if other relevant parameters, e.g., pH and ionic strength of the liquid; topography, zeta potential, and surface tension of the solid surfaces; are included in the statistical correlation, protein adsorption could be better predicted, and protein-specialized materials could be designed.

The flexibility of the protein could impact on the validity of the analysis based on protein structures that are assumed to be rigid in contact with probing objects that are rigid. Indeed, it was elegantly demonstrated [30], [31] that proteins with very different shapes, i.e., albumin and fibrinogen, present opposite denaturation behavior when presented to nanoparticles with different radii. Also, it has been demonstrated [28] that the size of nanoparticles play an important role in determining the nanoparticle coronas on different particles of identical materials. However, it has to be noted that the cases mentioned above are extreme ones; and that, despite the general perception, proteins are rather rigid, plexiglass-like, at their core. [67]

## Conclusion

The mapping and the quantification of the physico-chemical properties on the molecular surfaces of proteins using probes with increasing sizes offers insights into the interaction of proteins with nano-sized objects, or more generally with artificial solid surfaces. The geometrical and physico-chemical mapping of the molecular surfaces for a set of model proteins comprising various classes offered examples of this analysis, such as the protein-specific propensity for protecting the hydrophobic core. The relevance of the molecular surface-derived properties has been demonstrated via the calculation of the statistical strength of the prediction of protein adsorption. It is expected that the extension of this methodology to other protein:solid surface phenomena, in particular the interaction of nanoparticles, will result in important benefits in the understanding and design of protein-tailored solid surfaces.

## Supporting Information

File S1 (XLSX)

File S2 (XLSX)

File S3 (DOC)

File S4 (DOC)

File S5 (XLSX)
