# Supplementary material for: Protein Molecular Surface Mapped at Different Geometrical Resolutions
Source: PLoS One. 2013 Mar 14;8(3):e58896. doi: 10.1371/journal.pone.0058896 (PMC3597524; doi:10.1371/journal.pone.0058896)
Supplement: File S3 — (DOC) [file pone.0058896.s003.doc]

**Protein molecular surface mapped at different geometrical resolutions**

**Dan V Nicolau,^1,2^ Ewa Paszek,^1^ Florin Fulga,^1^ Dan V. Nicolau Jr.^3^**

*^1^Department of Electrical Engineering & Electronics, University of Liverpool, Liverpool, UK*

*^2^Department of Bioengineering, McGill University, Montreal, Canada*

*^3^Department of Integrative Biology, University of California at Berkeley, Berkeley, USA*

**Supplementary Information S3**

**Structure similarity calculation for the Hemoglobin subset comprising 572 residues**

Calculated using the Protein structure comparison service Fold at European Bioinformatics Institute (http://www.ebi.ac.uk/msd-srv/ssm), authored by E. Krissinel and K. Henrick

| **##** | **Structure** | **N_res_** | **N_SSE_** | **Consensus scores** | |
| --- | --- | --- | --- | --- | --- |
|  |  |  |  | **RMSD** | **Q-score** |
| 1 | PDB 1buw:* | 570 | 33 | 2.9164 | 0.4871 |
| 2 | PDB 1y4f:* | 574 | 33 | 0.4389 | 0.9211 |
| 3 | PDB 1a01:* | 574 | 33 | 0.3578 | 0.9276 |
| 4 | PDB 1y4p:* | 574 | 33 | 0.4968 | 0.9157 |
| 5 | PDB 1a00:* | 574 | 32 | 0.3808 | 0.9258 |
| 6 | PDB 1y46:* | 574 | 33 | 0.4334 | 0.9215 |
| 7 | PDB 2hhb:* | 574 | 32 | 0.3631 | 0.9272 |
| 8 | PDB 1y4g:* | 574 | 32 | 0.5296 | 0.9123 |
| 9 | PDB 1a0u:* | 574 | 34 | 0.4116 | 0.9234 |
| 10 | PDB 1a0z:* | 574 | 33 | 0.3803 | 0.9259 |
|  | | | | | |
| | Number of aligned residues | 540 |  | Overall RMSD | 1.501 | | --- | --- | --- | --- | --- | | Number of aligned SSEs | 15 | Overall Q-score | 0.7129 | | | | | | |

Cross-structure statistics

| **RMSD** |
| --- |
| | structure: | | **1** | **2** | **3** | **4** | **5** | **6** | **7** | **8** | **9** | **10** | | --- | --- | --- | --- | --- | --- | --- | --- | --- | --- | --- | --- | | **1** | PDB 1buw:A,B,C,D |  | 3.320 | 3.134 | 3.345 | 3.197 | 3.312 | 3.123 | 3.358 | 3.284 | 3.185 | | **2** | PDB 1y4f:A,C,B,D | 3.320 |  | 0.478 | 0.136 | 0.428 | 0.158 | 0.513 | 0.187 | 0.262 | 0.462 | | **3** | PDB 1a01:A,B,C,D | 3.134 | 0.478 |  | 0.547 | 0.383 | 0.460 | 0.191 | 0.590 | 0.437 | 0.358 | | **4** | PDB 1y4p:A,C,B,D | 3.345 | 0.136 | 0.547 |  | 0.505 | 0.241 | 0.590 | 0.104 | 0.355 | 0.547 | | **5** | PDB 1a00:A,B,C,D | 3.197 | 0.428 | 0.383 | 0.505 |  | 0.427 | 0.374 | 0.541 | 0.375 | 0.137 | | **6** | PDB 1y46:A,C,B,D | 3.312 | 0.158 | 0.460 | 0.241 | 0.427 |  | 0.479 | 0.288 | 0.231 | 0.444 | | **7** | PDB 2hhb:A,B,C,D | 3.123 | 0.513 | 0.191 | 0.590 | 0.374 | 0.479 |  | 0.630 | 0.419 | 0.327 | | **8** | PDB 1y4g:A,C,B,D | 3.358 | 0.187 | 0.590 | 0.104 | 0.541 | 0.288 | 0.630 |  | 0.391 | 0.582 | | **9** | PDB 1a0u:A,B,C,D | 3.284 | 0.262 | 0.437 | 0.355 | 0.375 | 0.231 | 0.419 | 0.391 |  | 0.363 | | **10** | PDB 1a0z:A,B,C,D | 3.185 | 0.462 | 0.358 | 0.547 | 0.137 | 0.444 | 0.327 | 0.582 | 0.363 |  | |

| **Q-score** |
| --- |
| | structure: | | **1** | **2** | **3** | **4** | **5** | **6** | **7** | **8** | **9** | **10** | | --- | --- | --- | --- | --- | --- | --- | --- | --- | --- | --- | --- | | **1** | PDB 1buw:A,B,C,D |  | 0.401 | 0.426 | 0.397 | 0.417 | 0.402 | 0.428 | 0.396 | 0.405 | 0.419 | | **2** | PDB 1y4f:A,C,B,D | 0.401 |  | 0.863 | 0.883 | 0.867 | 0.883 | 0.860 | 0.882 | 0.878 | 0.865 | | **3** | PDB 1a01:A,B,C,D | 0.426 | 0.863 |  | 0.857 | 0.871 | 0.865 | 0.881 | 0.852 | 0.867 | 0.873 | | **4** | PDB 1y4p:A,C,B,D | 0.397 | 0.883 | 0.857 |  | 0.861 | 0.879 | 0.852 | 0.884 | 0.873 | 0.857 | | **5** | PDB 1a00:A,B,C,D | 0.417 | 0.867 | 0.871 | 0.861 |  | 0.867 | 0.872 | 0.857 | 0.871 | 0.883 | | **6** | PDB 1y46:A,C,B,D | 0.402 | 0.883 | 0.865 | 0.879 | 0.867 |  | 0.863 | 0.877 | 0.880 | 0.866 | | **7** | PDB 2hhb:A,B,C,D | 0.428 | 0.860 | 0.881 | 0.852 | 0.872 | 0.863 |  | 0.848 | 0.868 | 0.875 | | **8** | PDB 1y4g:A,C,B,D | 0.396 | 0.882 | 0.852 | 0.884 | 0.857 | 0.877 | 0.848 |  | 0.870 | 0.853 | | **9** | PDB 1a0u:A,B,C,D | 0.405 | 0.878 | 0.867 | 0.873 | 0.871 | 0.880 | 0.868 | 0.870 |  | 0.872 | | **10** | PDB 1a0z:A,B,C,D | 0.419 | 0.865 | 0.873 | 0.857 | 0.883 | 0.866 | 0.875 | 0.853 | 0.872 |  | |

| **Sequence Identity** |
| --- |
| | structure: | | **1** | **2** | **3** | **4** | **5** | **6** | **7** | **8** | **9** | **10** | | --- | --- | --- | --- | --- | --- | --- | --- | --- | --- | --- | --- | | **1** | PDB 1buw:A,B,C,D |  | 0.772 | 0.772 | 0.772 | 0.772 | 0.772 | 0.778 | 0.772 | 0.776 | 0.776 | | **2** | PDB 1y4f:A,C,B,D | 0.772 |  | 1.000 | 0.996 | 0.996 | 0.996 | 0.994 | 0.996 | 0.996 | 0.996 | | **3** | PDB 1a01:A,B,C,D | 0.772 | 1.000 |  | 0.996 | 0.996 | 0.996 | 0.994 | 0.996 | 0.996 | 0.996 | | **4** | PDB 1y4p:A,C,B,D | 0.772 | 0.996 | 0.996 |  | 0.996 | 0.996 | 0.994 | 0.996 | 0.996 | 0.996 | | **5** | PDB 1a00:A,B,C,D | 0.772 | 0.996 | 0.996 | 0.996 |  | 1.000 | 0.994 | 0.996 | 0.996 | 0.996 | | **6** | PDB 1y46:A,C,B,D | 0.772 | 0.996 | 0.996 | 0.996 | 1.000 |  | 0.994 | 0.996 | 0.996 | 0.996 | | **7** | PDB 2hhb:A,B,C,D | 0.778 | 0.994 | 0.994 | 0.994 | 0.994 | 0.994 |  | 0.994 | 0.998 | 0.998 | | **8** | PDB 1y4g:A,C,B,D | 0.772 | 0.996 | 0.996 | 0.996 | 0.996 | 0.996 | 0.994 |  | 0.996 | 0.996 | | **9** | PDB 1a0u:A,B,C,D | 0.776 | 0.996 | 0.996 | 0.996 | 0.996 | 0.996 | 0.998 | 0.996 |  | 1.000 | | **10** | PDB 1a0z:A,B,C,D | 0.776 | 0.996 | 0.996 | 0.996 | 0.996 | 0.996 | 0.998 | 0.996 | 1.000 |  | |

| Rotation-translation matrices of best superposition | | | | | | |
| --- | --- | --- | --- | --- | --- | --- |
|  | | | | | | |
| PDB 1buw:A,B,C,D |  | | -0.732 | 0.526 | -0.433 | | --- | --- | --- | | -0.620 | -0.778 | 0.103 | | -0.283 | 0.344 | 0.895 | | × | | X | | --- | | Y | | Z | | + | | 41.872 | | --- | | 89.542 | | -38.059 | |
|  | | | | | | |
| PDB 1y4f:A,C,B,D |  | | 1.000 | -0.000 | -0.000 | | --- | --- | --- | | 0.000 | 1.000 | 0.003 | | 0.000 | -0.003 | 1.000 | | × | | X | | --- | | Y | | Z | | + | | 0.052 | | --- | | 0.010 | | 0.164 | |
|  | | | | | | |
| PDB 1a01:A,B,C,D |  | | -0.084 | -0.904 | 0.420 | | --- | --- | --- | | 0.493 | -0.404 | -0.771 | | 0.866 | 0.143 | 0.480 | | × | | X | | --- | | Y | | Z | | + | | 16.446 | | --- | | 57.198 | | -45.393 | |
|  | | | | | | |
| PDB 1y4p:A,C,B,D |  | | 1.000 | -0.001 | 0.000 | | --- | --- | --- | | 0.001 | 1.000 | 0.005 | | -0.000 | -0.005 | 1.000 | | × | | X | | --- | | Y | | Z | | + | | 0.013 | | --- | | 0.033 | | 0.264 | |
|  | | | | | | |
| PDB 1a00:A,B,C,D |  | | -0.928 | -0.310 | -0.207 | | --- | --- | --- | | -0.144 | 0.810 | -0.568 | | 0.343 | -0.497 | -0.797 | | × | | X | | --- | | Y | | Z | | + | | 112.487 | | --- | | 4.738 | | -20.824 | |
|  | | | | | | |
| PDB 1y46:A,C,B,D |  | | 1.000 | 0.002 | 0.000 | | --- | --- | --- | | -0.002 | 1.000 | 0.003 | | -0.000 | -0.003 | 1.000 | | × | | X | | --- | | Y | | Z | | + | | -0.093 | | --- | | -0.009 | | 0.141 | |
|  | | | | | | |
| PDB 2hhb:A,B,C,D |  | | 0.037 | -0.940 | 0.338 | | --- | --- | --- | | 0.595 | -0.251 | -0.763 | | 0.803 | 0.229 | 0.550 | | × | | X | | --- | | Y | | Z | | + | | 19.946 | | --- | | 26.082 | | -15.346 | |
|  | | | | | | |
| PDB 1y4g:A,C,B,D |  | | 1.000 | -0.001 | 0.001 | | --- | --- | --- | | 0.001 | 1.000 | 0.006 | | -0.001 | -0.006 | 1.000 | | × | | X | | --- | | Y | | Z | | + | | 0.086 | | --- | | 0.021 | | 0.313 | |
|  | | | | | | |
| PDB 1a0u:A,B,C,D |  | | 1.000 | -0.000 | -0.000 | | --- | --- | --- | | 0.000 | 1.000 | -0.000 | | 0.000 | 0.000 | 1.000 | | × | | X | | --- | | Y | | Z | | + | | 0.002 | | --- | | -0.002 | | -0.003 | |
|  | | | | | | |
| PDB 1a0z:A,B,C,D |  | | -0.928 | -0.310 | -0.208 | | --- | --- | --- | | -0.142 | 0.809 | -0.570 | | 0.345 | -0.499 | -0.795 | | × | | X | | --- | | Y | | Z | | + | | 112.259 | | --- | | 4.595 | | -20.858 | |

3D Structural alignment

| **1buw** | |  | **1y4f** | |  | **1a01** | |  | **1y4p** | |  | **1a00** | |  | **1y46** | |  | **2hhb** | |  | **1y4g** | |  | **1a0u** | |  | **1a0z** | |
| --- | --- | --- | --- | --- | --- | --- | --- | --- | --- | --- | --- | --- | --- | --- | --- | --- | --- | --- | --- | --- | --- | --- | --- | --- | --- | --- | --- | --- |
|  | A:VAL   1 |  |  | |  |  | |  |  | |  |  | |  |  | |  |  | |  |  | |  |  | |  |  | |
|  | A:LEU   2 | ||| |  | A:VAL   1 | ||| |  | A:VAL   1 | ||| |  | A:VAL   1 | ||| |  | A:VAL   1 | ||| |  | A:VAL   1 | ||| |  | A:VAL   1 | ||| |  | A:VAL   1 | ||| |  | A:VAL   1 | ||| |  | A:VAL   1 |
| H | A:SER   3 |  |  | |  |  | |  |  | |  |  | |  |  | |  |  | |  |  | |  |  | |  |  | |
| H | A:PRO   4 |  |  | |  |  | |  |  | |  |  | |  |  | |  |  | |  |  | |  |  | |  |  | |
| H | A:ALA   5 |  |  | |  |  | |  |  | |  |  | |  |  | |  |  | |  |  | |  |  | |  |  | |
| H | A:ASP   6 | ||| |  | A:LEU   2 | ||| |  | A:LEU   2 | ||| |  | A:LEU   2 | ||| |  | A:LEU   2 | ||| |  | A:LEU   2 | ||| |  | A:LEU   2 | ||| |  | A:LEU   2 | ||| |  | A:LEU   2 | ||| |  | A:LEU   2 |
| H | A:LYS   7 | ||| | H | A:SER   3 | ||| | H | A:SER   3 | ||| | H | A:SER   3 | ||| | H | A:SER   3 | ||| | H | A:SER   3 | ||| | H | A:SER   3 | ||| | H | A:SER   3 | ||| | H | A:SER   3 | ||| | H | A:SER   3 |
| H | A:THR   8 | ||| | H | A:PRO   4 | ||| | H | A:PRO   4 | ||| | H | A:PRO   4 | ||| | H | A:PRO   4 | ||| | H | A:PRO   4 | ||| | H | A:PRO   4 | ||| | H | A:PRO   4 | ||| | H | A:PRO   4 | ||| | H | A:PRO   4 |
| H | A:ASN   9 | ||| | H | A:ALA   5 | ||| | H | A:ALA   5 | ||| | H | A:ALA   5 | ||| | H | A:ALA   5 | ||| | H | A:ALA   5 | ||| | H | A:ALA   5 | ||| | H | A:ALA   5 | ||| | H | A:ALA   5 | ||| | H | A:ALA   5 |
| H | A:VAL  10 | ||| | H | A:ASP   6 | ||| | H | A:ASP   6 | ||| | H | A:ASP   6 | ||| | H | A:ASP   6 | ||| | H | A:ASP   6 | ||| | H | A:ASP   6 | ||| | H | A:ASP   6 | ||| | H | A:ASP   6 | ||| | H | A:ASP   6 |
| H | A:LYS  11 | ||| | H | A:LYS   7 | ||| | H | A:LYS   7 | ||| | H | A:LYS   7 | ||| | H | A:LYS   7 | ||| | H | A:LYS   7 | ||| | H | A:LYS   7 | ||| | H | A:LYS   7 | ||| | H | A:LYS   7 | ||| | H | A:LYS   7 |
| H | A:ALA  12 | ||| | H | A:THR   8 | ||| | H | A:THR   8 | ||| | H | A:THR   8 | ||| | H | A:THR   8 | ||| | H | A:THR   8 | ||| | H | A:THR   8 | ||| | H | A:THR   8 | ||| | H | A:THR   8 | ||| | H | A:THR   8 |
| H | A:ALA  13 | ||| | H | A:ASN   9 | ||| | H | A:ASN   9 | ||| | H | A:ASN   9 | ||| | H | A:ASN   9 | ||| | H | A:ASN   9 | ||| | H | A:ASN   9 | ||| | H | A:ASN   9 | ||| | H | A:ASN   9 | ||| | H | A:ASN   9 |
| H | A:TRP  14 | ||| | H | A:VAL  10 | ||| | H | A:VAL  10 | ||| | H | A:VAL  10 | ||| | H | A:VAL  10 | ||| | H | A:VAL  10 | ||| | H | A:VAL  10 | ||| | H | A:VAL  10 | ||| | H | A:VAL  10 | ||| | H | A:VAL  10 |
| H | A:GLY  15 | ||| | H | A:LYS  11 | ||| | H | A:LYS  11 | ||| | H | A:LYS  11 | ||| | H | A:LYS  11 | ||| | H | A:LYS  11 | ||| | H | A:LYS  11 | ||| | H | A:LYS  11 | ||| | H | A:LYS  11 | ||| | H | A:LYS  11 |
| H | A:LYS  16 | ||| | H | A:ALA  12 | ||| | H | A:ALA  12 | ||| | H | A:ALA  12 | ||| | H | A:ALA  12 | ||| | H | A:ALA  12 | ||| | H | A:ALA  12 | ||| | H | A:ALA  12 | ||| | H | A:ALA  12 | ||| | H | A:ALA  12 |
| H | A:VAL  17 | ||| | H | A:ALA  13 | ||| | H | A:ALA  13 | ||| | H | A:ALA  13 | ||| | H | A:ALA  13 | ||| | H | A:ALA  13 | ||| | H | A:ALA  13 | ||| | H | A:ALA  13 | ||| | H | A:ALA  13 | ||| | H | A:ALA  13 |
| H | A:GLY  18 | ||| | H | A:TRP  14 | ||| | H | A:TRP  14 | ||| | H | A:TRP  14 | ||| | H | A:TRP  14 | ||| | H | A:TRP  14 | ||| | H | A:TRP  14 | ||| | H | A:TRP  14 | ||| | H | A:TRP  14 | ||| | H | A:TRP  14 |
|  | |  | H | A:GLY  15 |  | H | A:GLY  15 |  | H | A:GLY  15 |  | H | A:GLY  15 |  | H | A:GLY  15 |  | H | A:GLY  15 |  | H | A:GLY  15 |  | H | A:GLY  15 |  | H | A:GLY  15 |
|  | |  | H | A:LYS  16 |  | H | A:LYS  16 |  | H | A:LYS  16 |  | H | A:LYS  16 |  | H | A:LYS  16 |  | H | A:LYS  16 |  | H | A:LYS  16 |  | H | A:LYS  16 |  | H | A:LYS  16 |
|  | |  | H | A:VAL  17 |  | H | A:VAL  17 |  | H | A:VAL  17 |  | H | A:VAL  17 |  | H | A:VAL  17 |  | H | A:VAL  17 |  | H | A:VAL  17 |  | H | A:VAL  17 |  | H | A:VAL  17 |
|  | |  | H | A:GLY  18 |  | H | A:GLY  18 |  | H | A:GLY  18 |  | H | A:GLY  18 |  | H | A:GLY  18 |  | H | A:GLY  18 |  | H | A:GLY  18 |  | H | A:GLY  18 |  | H | A:GLY  18 |
|  | A:ALA  19 | ||| |  | A:ALA  19 | ||| |  | A:ALA  19 | ||| |  | A:ALA  19 | ||| |  | A:ALA  19 | ||| |  | A:ALA  19 | ||| |  | A:ALA  19 | ||| |  | A:ALA  19 | ||| |  | A:ALA  19 | ||| |  | A:ALA  19 |
| H | A:HIS  20 | ||| | H | A:HIS  20 | ||| | H | A:HIS  20 | ||| | H | A:HIS  20 | ||| | H | A:HIS  20 | ||| | H | A:HIS  20 | ||| | H | A:HIS  20 | ||| | H | A:HIS  20 | ||| | H | A:HIS  20 | ||| | H | A:HIS  20 |
| H | A:ALA  21 | ||| | H | A:ALA  21 | ||| | H | A:ALA  21 | ||| | H | A:ALA  21 | ||| | H | A:ALA  21 | ||| | H | A:ALA  21 | ||| | H | A:ALA  21 | ||| | H | A:ALA  21 | ||| | H | A:ALA  21 | ||| | H | A:ALA  21 |
| H | A:GLY  22 | ||| | H | A:GLY  22 | ||| | H | A:GLY  22 | ||| | H | A:GLY  22 | ||| | H | A:GLY  22 | ||| | H | A:GLY  22 | ||| | H | A:GLY  22 | ||| | H | A:GLY  22 | ||| | H | A:GLY  22 | ||| | H | A:GLY  22 |
| H | A:GLU  23 | ||| | H | A:GLU  23 | ||| | H | A:GLU  23 | ||| | H | A:GLU  23 | ||| | H | A:GLU  23 | ||| | H | A:GLU  23 | ||| | H | A:GLU  23 | ||| | H | A:GLU  23 | ||| | H | A:GLU  23 | ||| | H | A:GLU  23 |
| H | A:TYR  24 | ||| | H | A:TYR  24 | ||| | H | A:TYR  24 | ||| | H | A:TYR  24 | ||| | H | A:TYR  24 | ||| | H | A:TYR  24 | ||| | H | A:TYR  24 | ||| | H | A:TYR  24 | ||| | H | A:TYR  24 | ||| | H | A:TYR  24 |
| H | A:GLY  25 | ||| | H | A:GLY  25 | ||| | H | A:GLY  25 | ||| | H | A:GLY  25 | ||| | H | A:GLY  25 | ||| | H | A:GLY  25 | ||| | H | A:GLY  25 | ||| | H | A:GLY  25 | ||| | H | A:GLY  25 | ||| | H | A:GLY  25 |
| H | A:ALA  26 | ||| | H | A:ALA  26 | ||| | H | A:ALA  26 | ||| | H | A:ALA  26 | ||| | H | A:ALA  26 | ||| | H | A:ALA  26 | ||| | H | A:ALA  26 | ||| | H | A:ALA  26 | ||| | H | A:ALA  26 | ||| | H | A:ALA  26 |
| H | A:GLU  27 | ||| | H | A:GLU  27 | ||| | H | A:GLU  27 | ||| | H | A:GLU  27 | ||| | H | A:GLU  27 | ||| | H | A:GLU  27 | ||| | H | A:GLU  27 | ||| | H | A:GLU  27 | ||| | H | A:GLU  27 | ||| | H | A:GLU  27 |
| H | A:ALA  28 | ||| | H | A:ALA  28 | ||| | H | A:ALA  28 | ||| | H | A:ALA  28 | ||| | H | A:ALA  28 | ||| | H | A:ALA  28 | ||| | H | A:ALA  28 | ||| | H | A:ALA  28 | ||| | H | A:ALA  28 | ||| | H | A:ALA  28 |
| H | A:LEU  29 | ||| | H | A:LEU  29 | ||| | H | A:LEU  29 | ||| | H | A:LEU  29 | ||| | H | A:LEU  29 | ||| | H | A:LEU  29 | ||| | H | A:LEU  29 | ||| | H | A:LEU  29 | ||| | H | A:LEU  29 | ||| | H | A:LEU  29 |
| H | A:GLU  30 | ||| | H | A:GLU  30 | ||| | H | A:GLU  30 | ||| | H | A:GLU  30 | ||| | H | A:GLU  30 | ||| | H | A:GLU  30 | ||| | H | A:GLU  30 | ||| | H | A:GLU  30 | ||| | H | A:GLU  30 | ||| | H | A:GLU  30 |
| H | A:ARG  31 | ||| | H | A:ARG  31 | ||| | H | A:ARG  31 | ||| | H | A:ARG  31 | ||| | H | A:ARG  31 | ||| | H | A:ARG  31 | ||| | H | A:ARG  31 | ||| | H | A:ARG  31 | ||| | H | A:ARG  31 | ||| | H | A:ARG  31 |
| H | A:MET  32 | ||| | H | A:MET  32 | ||| | H | A:MET  32 | ||| | H | A:MET  32 | ||| | H | A:MET  32 | ||| | H | A:MET  32 | ||| | H | A:MET  32 | ||| | H | A:MET  32 | ||| | H | A:MET  32 | ||| | H | A:MET  32 |
| H | A:PHE  33 | ||| | H | A:PHE  33 | ||| | H | A:PHE  33 | ||| | H | A:PHE  33 | ||| | H | A:PHE  33 | ||| | H | A:PHE  33 | ||| | H | A:PHE  33 | ||| | H | A:PHE  33 | ||| | H | A:PHE  33 | ||| | H | A:PHE  33 |
| H | A:LEU  34 | ||| | H | A:LEU  34 | ||| | H | A:LEU  34 | ||| | H | A:LEU  34 | ||| | H | A:LEU  34 | ||| | H | A:LEU  34 | ||| | H | A:LEU  34 | ||| | H | A:LEU  34 | ||| | H | A:LEU  34 | ||| | H | A:LEU  34 |
| H | A:SER  35 | ||| | H | A:SER  35 | ||| | H | A:SER  35 | ||| | H | A:SER  35 | ||| | H | A:SER  35 | ||| | H | A:SER  35 | ||| | H | A:SER  35 | ||| | H | A:SER  35 | ||| | H | A:SER  35 | ||| | H | A:SER  35 |
| H | A:PHE  36 | ||| | H | A:PHE  36 | ||| | H | A:PHE  36 | ||| | H | A:PHE  36 | ||| | H | A:PHE  36 | ||| | H | A:PHE  36 | ||| | H | A:PHE  36 | ||| | H | A:PHE  36 | ||| | H | A:PHE  36 | ||| | H | A:PHE  36 |
| H | A:PRO  37 | ||| | H | A:PRO  37 | ||| | H | A:PRO  37 | ||| | H | A:PRO  37 | ||| | H | A:PRO  37 | ||| | H | A:PRO  37 | ||| | H | A:PRO  37 | ||| | H | A:PRO  37 | ||| | H | A:PRO  37 | ||| | H | A:PRO  37 |
| H | A:THR  38 | ||| | H | A:THR  38 | ||| | H | A:THR  38 | ||| | H | A:THR  38 | ||| | H | A:THR  38 | ||| | H | A:THR  38 | ||| | H | A:THR  38 | ||| | H | A:THR  38 | ||| | H | A:THR  38 | ||| | H | A:THR  38 |
| H | A:THR  39 | ||| | H | A:THR  39 | ||| | H | A:THR  39 | ||| | H | A:THR  39 | ||| | H | A:THR  39 | ||| | H | A:THR  39 | ||| | H | A:THR  39 | ||| | H | A:THR  39 | ||| | H | A:THR  39 | ||| | H | A:THR  39 |
| H | A:LYS  40 | ||| | H | A:LYS  40 | ||| | H | A:LYS  40 | ||| | H | A:LYS  40 | ||| | H | A:LYS  40 | ||| | H | A:LYS  40 | ||| | H | A:LYS  40 | ||| | H | A:LYS  40 | ||| | H | A:LYS  40 | ||| | H | A:LYS  40 |
| H | A:THR  41 | ||| | H | A:THR  41 | ||| | H | A:THR  41 | ||| | H | A:THR  41 | ||| | H | A:THR  41 | ||| | H | A:THR  41 | ||| | H | A:THR  41 | ||| | H | A:THR  41 | ||| | H | A:THR  41 | ||| | H | A:THR  41 |
| H | A:TYR  42 | ||| | H | A:TYR  42 | ||| | H | A:TYR  42 | ||| | H | A:TYR  42 | ||| | H | A:TYR  42 | ||| | H | A:TYR  42 | ||| | H | A:TYR  42 | ||| | H | A:TYR  42 | ||| | H | A:TYR  42 | ||| | H | A:TYR  42 |
| H | A:PHE  43 | ||| | H | A:PHE  43 | ||| | H | A:PHE  43 | ||| | H | A:PHE  43 | ||| | H | A:PHE  43 | ||| | H | A:PHE  43 | ||| | H | A:PHE  43 | ||| | H | A:PHE  43 | ||| | H | A:PHE  43 | ||| | H | A:PHE  43 |
|  | A:PRO  44 | ||| |  | A:PRO  44 | ||| |  | A:PRO  44 | ||| |  | A:PRO  44 | ||| |  | A:PRO  44 | ||| |  | A:PRO  44 | ||| |  | A:PRO  44 | ||| |  | A:PRO  44 | ||| |  | A:PRO  44 | ||| |  | A:PRO  44 |
|  | A:HIS  45 | ||| |  | A:HIS  45 | ||| |  | A:HIS  45 | ||| |  | A:HIS  45 | ||| |  | A:HIS  45 | ||| |  | A:HIS  45 | ||| |  | A:HIS  45 | ||| |  | A:HIS  45 | ||| |  | A:HIS  45 | ||| |  | A:HIS  45 |
|  | A:PHE  46 | ||| |  | A:PHE  46 | ||| |  | A:PHE  46 | ||| |  | A:PHE  46 | ||| |  | A:PHE  46 | ||| |  | A:PHE  46 | ||| |  | A:PHE  46 | ||| |  | A:PHE  46 | ||| |  | A:PHE  46 | ||| |  | A:PHE  46 |
|  | A:ASP  47 | ||| |  | A:ASP  47 | ||| |  | A:ASP  47 | ||| |  | A:ASP  47 | ||| |  | A:ASP  47 | ||| |  | A:ASP  47 | ||| |  | A:ASP  47 | ||| |  | A:ASP  47 | ||| |  | A:ASP  47 | ||| |  | A:ASP  47 |
|  | A:LEU  48 | ||| |  | A:LEU  48 | ||| |  | A:LEU  48 | ||| |  | A:LEU  48 | ||| |  | A:LEU  48 | ||| |  | A:LEU  48 | ||| |  | A:LEU  48 | ||| |  | A:LEU  48 | ||| |  | A:LEU  48 | ||| |  | A:LEU  48 |
|  | A:SER  49 | ||| |  | A:SER  49 | ||| |  | A:SER  49 | ||| |  | A:SER  49 | ||| |  | A:SER  49 | ||| |  | A:SER  49 | ||| |  | A:SER  49 | ||| |  | A:SER  49 | ||| |  | A:SER  49 | ||| |  | A:SER  49 |
|  | A:HIS  50 | ||| |  | A:HIS  50 | ||| |  | A:HIS  50 | ||| |  | A:HIS  50 | ||| |  | A:HIS  50 | ||| |  | A:HIS  50 | ||| |  | A:HIS  50 | ||| |  | A:HIS  50 | ||| |  | A:HIS  50 | ||| |  | A:HIS  50 |
|  | A:GLY  51 | ||| |  | A:GLY  51 | ||| |  | A:GLY  51 | ||| |  | A:GLY  51 | ||| |  | A:GLY  51 | ||| |  | A:GLY  51 | ||| |  | A:GLY  51 | ||| |  | A:GLY  51 | ||| |  | A:GLY  51 | ||| |  | A:GLY  51 |
| H | A:SER  52 | ||| | H | A:SER  52 | ||| | H | A:SER  52 | ||| | H | A:SER  52 | ||| | H | A:SER  52 | ||| | H | A:SER  52 | ||| | H | A:SER  52 | ||| | H | A:SER  52 | ||| | H | A:SER  52 | ||| | H | A:SER  52 |
| H | A:ALA  53 | ||| | H | A:ALA  53 | ||| | H | A:ALA  53 | ||| | H | A:ALA  53 | ||| | H | A:ALA  53 | ||| | H | A:ALA  53 | ||| | H | A:ALA  53 | ||| | H | A:ALA  53 | ||| | H | A:ALA  53 | ||| | H | A:ALA  53 |
| H | A:GLN  54 | ||| | H | A:GLN  54 | ||| | H | A:GLN  54 | ||| | H | A:GLN  54 | ||| | H | A:GLN  54 | ||| | H | A:GLN  54 | ||| | H | A:GLN  54 | ||| | H | A:GLN  54 | ||| | H | A:GLN  54 | ||| | H | A:GLN  54 |
| H | A:VAL  55 | ||| | H | A:VAL  55 | ||| | H | A:VAL  55 | ||| | H | A:VAL  55 | ||| | H | A:VAL  55 | ||| | H | A:VAL  55 | ||| | H | A:VAL  55 | ||| | H | A:VAL  55 | ||| | H | A:VAL  55 | ||| | H | A:VAL  55 |
| H | A:LYS  56 | ||| | H | A:LYS  56 | ||| | H | A:LYS  56 | ||| | H | A:LYS  56 | ||| | H | A:LYS  56 | ||| | H | A:LYS  56 | ||| | H | A:LYS  56 | ||| | H | A:LYS  56 | ||| | H | A:LYS  56 | ||| | H | A:LYS  56 |
| H | A:GLY  57 | ||| | H | A:GLY  57 | ||| | H | A:GLY  57 | ||| | H | A:GLY  57 | ||| | H | A:GLY  57 | ||| | H | A:GLY  57 | ||| | H | A:GLY  57 | ||| | H | A:GLY  57 | ||| | H | A:GLY  57 | ||| | H | A:GLY  57 |
| H | A:HIS  58 | ||| | H | A:HIS  58 | ||| | H | A:HIS  58 | ||| | H | A:HIS  58 | ||| | H | A:HIS  58 | ||| | H | A:HIS  58 | ||| | H | A:HIS  58 | ||| | H | A:HIS  58 | ||| | H | A:HIS  58 | ||| | H | A:HIS  58 |
| H | A:GLY  59 | ||| | H | A:GLY  59 | ||| | H | A:GLY  59 | ||| | H | A:GLY  59 | ||| | H | A:GLY  59 | ||| | H | A:GLY  59 | ||| | H | A:GLY  59 | ||| | H | A:GLY  59 | ||| | H | A:GLY  59 | ||| | H | A:GLY  59 |
| H | A:LYS  60 | ||| | H | A:LYS  60 | ||| | H | A:LYS  60 | ||| | H | A:LYS  60 | ||| | H | A:LYS  60 | ||| | H | A:LYS  60 | ||| | H | A:LYS  60 | ||| | H | A:LYS  60 | ||| | H | A:LYS  60 | ||| | H | A:LYS  60 |
| H | A:LYS  61 | ||| | H | A:LYS  61 | ||| | H | A:LYS  61 | ||| | H | A:LYS  61 | ||| | H | A:LYS  61 | ||| | H | A:LYS  61 | ||| | H | A:LYS  61 | ||| | H | A:LYS  61 | ||| | H | A:LYS  61 | ||| | H | A:LYS  61 |
| H | A:VAL  62 | ||| | H | A:VAL  62 | ||| | H | A:VAL  62 | ||| | H | A:VAL  62 | ||| | H | A:VAL  62 | ||| | H | A:VAL  62 | ||| | H | A:VAL  62 | ||| | H | A:VAL  62 | ||| | H | A:VAL  62 | ||| | H | A:VAL  62 |
| H | A:ALA  63 | ||| | H | A:ALA  63 | ||| | H | A:ALA  63 | ||| | H | A:ALA  63 | ||| | H | A:ALA  63 | ||| | H | A:ALA  63 | ||| | H | A:ALA  63 | ||| | H | A:ALA  63 | ||| | H | A:ALA  63 | ||| | H | A:ALA  63 |
| H | A:ASP  64 | ||| | H | A:ASP  64 | ||| | H | A:ASP  64 | ||| | H | A:ASP  64 | ||| | H | A:ASP  64 | ||| | H | A:ASP  64 | ||| | H | A:ASP  64 | ||| | H | A:ASP  64 | ||| | H | A:ASP  64 | ||| | H | A:ASP  64 |
| H | A:ALA  65 | ||| | H | A:ALA  65 | ||| | H | A:ALA  65 | ||| | H | A:ALA  65 | ||| | H | A:ALA  65 | ||| | H | A:ALA  65 | ||| | H | A:ALA  65 | ||| | H | A:ALA  65 | ||| | H | A:ALA  65 | ||| | H | A:ALA  65 |
| H | A:LEU  66 | ||| | H | A:LEU  66 | ||| | H | A:LEU  66 | ||| | H | A:LEU  66 | ||| | H | A:LEU  66 | ||| | H | A:LEU  66 | ||| | H | A:LEU  66 | ||| | H | A:LEU  66 | ||| | H | A:LEU  66 | ||| | H | A:LEU  66 |
| H | A:THR  67 | ||| | H | A:THR  67 | ||| | H | A:THR  67 | ||| | H | A:THR  67 | ||| | H | A:THR  67 | ||| | H | A:THR  67 | ||| | H | A:THR  67 | ||| | H | A:THR  67 | ||| | H | A:THR  67 | ||| | H | A:THR  67 |
| H | A:ASN  68 | ||| | H | A:ASN  68 | ||| | H | A:ASN  68 | ||| | H | A:ASN  68 | ||| | H | A:ASN  68 | ||| | H | A:ASN  68 | ||| | H | A:ASN  68 | ||| | H | A:ASN  68 | ||| | H | A:ASN  68 | ||| | H | A:ASN  68 |
| H | A:ALA  69 | ||| | H | A:ALA  69 | ||| | H | A:ALA  69 | ||| | H | A:ALA  69 | ||| | H | A:ALA  69 | ||| | H | A:ALA  69 | ||| | H | A:ALA  69 | ||| | H | A:ALA  69 | ||| | H | A:ALA  69 | ||| | H | A:ALA  69 |
| H | A:VAL  70 | ||| | H | A:VAL  70 | ||| | H | A:VAL  70 | ||| | H | A:VAL  70 | ||| | H | A:VAL  70 | ||| | H | A:VAL  70 | ||| | H | A:VAL  70 | ||| | H | A:VAL  70 | ||| | H | A:VAL  70 | ||| | H | A:VAL  70 |
| H | A:ALA  71 | ||| | H | A:ALA  71 | ||| | H | A:ALA  71 | ||| | H | A:ALA  71 | ||| | H | A:ALA  71 | ||| | H | A:ALA  71 | ||| | H | A:ALA  71 | ||| | H | A:ALA  71 | ||| | H | A:ALA  71 | ||| | H | A:ALA  71 |
| H | A:HIS  72 | ||| | H | A:HIS  72 | ||| | H | A:HIS  72 | ||| | H | A:HIS  72 | ||| | H | A:HIS  72 | ||| | H | A:HIS  72 | ||| | H | A:HIS  72 | ||| | H | A:HIS  72 | ||| | H | A:HIS  72 | ||| | H | A:HIS  72 |
|  | A:VAL  73 | ||| |  | A:VAL  73 | ||| |  | A:VAL  73 | ||| |  | A:VAL  73 | ||| |  | A:VAL  73 | ||| |  | A:VAL  73 | ||| |  | A:VAL  73 | ||| |  | A:VAL  73 | ||| |  | A:VAL  73 | ||| |  | A:VAL  73 |
|  | A:ASP  74 | ||| |  | A:ASP  74 | ||| |  | A:ASP  74 | ||| |  | A:ASP  74 | ||| |  | A:ASP  74 | ||| |  | A:ASP  74 | ||| |  | A:ASP  74 | ||| |  | A:ASP  74 | ||| |  | A:ASP  74 | ||| |  | A:ASP  74 |
| H | A:ASP  75 | ||| | H | A:ASP  75 | ||| | H | A:ASP  75 | ||| | H | A:ASP  75 | ||| | H | A:ASP  75 | ||| | H | A:ASP  75 | ||| | H | A:ASP  75 | ||| | H | A:ASP  75 | ||| | H | A:ASP  75 | ||| | H | A:ASP  75 |
| H | A:MET  76 | ||| | H | A:MET  76 | ||| | H | A:MET  76 | ||| | H | A:MET  76 | ||| | H | A:MET  76 | ||| | H | A:MET  76 | ||| | H | A:MET  76 | ||| | H | A:MET  76 | ||| | H | A:MET  76 | ||| | H | A:MET  76 |
| H | A:PRO  77 | ||| | H | A:PRO  77 | ||| | H | A:PRO  77 | ||| | H | A:PRO  77 | ||| | H | A:PRO  77 | ||| | H | A:PRO  77 | ||| | H | A:PRO  77 | ||| | H | A:PRO  77 | ||| | H | A:PRO  77 | ||| | H | A:PRO  77 |
| H | A:ASN  78 | ||| | H | A:ASN  78 | ||| | H | A:ASN  78 | ||| | H | A:ASN  78 | ||| | H | A:ASN  78 | ||| | H | A:ASN  78 | ||| | H | A:ASN  78 | ||| | H | A:ASN  78 | ||| | H | A:ASN  78 | ||| | H | A:ASN  78 |
| H | A:ALA  79 | ||| | H | A:ALA  79 | ||| | H | A:ALA  79 | ||| | H | A:ALA  79 | ||| | H | A:ALA  79 | ||| | H | A:ALA  79 | ||| | H | A:ALA  79 | ||| | H | A:ALA  79 | ||| | H | A:ALA  79 | ||| | H | A:ALA  79 |
| H | A:LEU  80 | ||| | H | A:LEU  80 | ||| | H | A:LEU  80 | ||| | H | A:LEU  80 | ||| | H | A:LEU  80 | ||| | H | A:LEU  80 | ||| | H | A:LEU  80 | ||| | H | A:LEU  80 | ||| | H | A:LEU  80 | ||| | H | A:LEU  80 |
| H | A:SER  81 | ||| | H | A:SER  81 | ||| | H | A:SER  81 | ||| | H | A:SER  81 | ||| | H | A:SER  81 | ||| | H | A:SER  81 | ||| | H | A:SER  81 | ||| | H | A:SER  81 | ||| | H | A:SER  81 | ||| | H | A:SER  81 |
| H | A:ALA  82 | ||| | H | A:ALA  82 | ||| | H | A:ALA  82 | ||| | H | A:ALA  82 | ||| | H | A:ALA  82 | ||| | H | A:ALA  82 | ||| | H | A:ALA  82 | ||| | H | A:ALA  82 | ||| | H | A:ALA  82 | ||| | H | A:ALA  82 |
| H | A:LEU  83 | ||| | H | A:LEU  83 | ||| | H | A:LEU  83 | ||| | H | A:LEU  83 | ||| | H | A:LEU  83 | ||| | H | A:LEU  83 | ||| | H | A:LEU  83 | ||| | H | A:LEU  83 | ||| | H | A:LEU  83 | ||| | H | A:LEU  83 |
| H | A:SER  84 | ||| | H | A:SER  84 | ||| | H | A:SER  84 | ||| | H | A:SER  84 | ||| | H | A:SER  84 | ||| | H | A:SER  84 | ||| | H | A:SER  84 | ||| | H | A:SER  84 | ||| | H | A:SER  84 | ||| | H | A:SER  84 |
| H | A:ASP  85 | ||| | H | A:ASP  85 | ||| | H | A:ASP  85 | ||| | H | A:ASP  85 | ||| | H | A:ASP  85 | ||| | H | A:ASP  85 | ||| | H | A:ASP  85 | ||| | H | A:ASP  85 | ||| | H | A:ASP  85 | ||| | H | A:ASP  85 |
| H | A:LEU  86 | ||| | H | A:LEU  86 | ||| | H | A:LEU  86 | ||| | H | A:LEU  86 | ||| | H | A:LEU  86 | ||| | H | A:LEU  86 | ||| | H | A:LEU  86 | ||| | H | A:LEU  86 | ||| | H | A:LEU  86 | ||| | H | A:LEU  86 |
| H | A:HIS  87 | ||| | H | A:HIS  87 | ||| | H | A:HIS  87 | ||| | H | A:HIS  87 | ||| | H | A:HIS  87 | ||| | H | A:HIS  87 | ||| | H | A:HIS  87 | ||| | H | A:HIS  87 | ||| | H | A:HIS  87 | ||| | H | A:HIS  87 |
| H | A:ALA  88 | ||| | H | A:ALA  88 | ||| | H | A:ALA  88 | ||| | H | A:ALA  88 | ||| | H | A:ALA  88 | ||| | H | A:ALA  88 | ||| | H | A:ALA  88 | ||| | H | A:ALA  88 | ||| | H | A:ALA  88 | ||| | H | A:ALA  88 |
| H | A:HIS  89 | ||| | H | A:HIS  89 | ||| | H | A:HIS  89 | ||| | H | A:HIS  89 | ||| | H | A:HIS  89 | ||| | H | A:HIS  89 | ||| | H | A:HIS  89 | ||| | H | A:HIS  89 | ||| | H | A:HIS  89 | ||| | H | A:HIS  89 |
|  | A:LYS  90 | ||| | H | A:LYS  90 | ||| | H | A:LYS  90 | ||| | H | A:LYS  90 | ||| | H | A:LYS  90 | ||| | H | A:LYS  90 | ||| | H | A:LYS  90 | ||| | H | A:LYS  90 | ||| | H | A:LYS  90 | ||| | H | A:LYS  90 |
|  | A:LEU  91 | ||| |  | A:LEU  91 | ||| |  | A:LEU  91 | ||| |  | A:LEU  91 | ||| |  | A:LEU  91 | ||| |  | A:LEU  91 | ||| |  | A:LEU  91 | ||| |  | A:LEU  91 | ||| |  | A:LEU  91 | ||| |  | A:LEU  91 |
|  | A:ARG  92 | ||| |  | A:ARG  92 | ||| |  | A:ARG  92 | ||| |  | A:ARG  92 | ||| |  | A:ARG  92 | ||| |  | A:ARG  92 | ||| |  | A:ARG  92 | ||| |  | A:ARG  92 | ||| |  | A:ARG  92 | ||| |  | A:ARG  92 |
|  | A:VAL  93 | ||| |  | A:VAL  93 | ||| |  | A:VAL  93 | ||| |  | A:VAL  93 | ||| |  | A:VAL  93 | ||| |  | A:VAL  93 | ||| |  | A:VAL  93 | ||| |  | A:VAL  93 | ||| |  | A:VAL  93 | ||| |  | A:VAL  93 |
|  | A:ASP  94 | ||| |  | A:ASP  94 | ||| |  | A:ASP  94 | ||| |  | A:ASP  94 | ||| |  | A:ASP  94 | ||| |  | A:ASP  94 | ||| |  | A:ASP  94 | ||| |  | A:ASP  94 | ||| |  | A:ASP  94 | ||| |  | A:ASP  94 |
| H | A:PRO  95 | ||| | H | A:PRO  95 | ||| | H | A:PRO  95 | ||| | H | A:PRO  95 | ||| | H | A:PRO  95 | ||| | H | A:PRO  95 | ||| | H | A:PRO  95 | ||| | H | A:PRO  95 | ||| | H | A:PRO  95 | ||| | H | A:PRO  95 |
| H | A:VAL  96 | ||| | H | A:VAL  96 | ||| | H | A:VAL  96 | ||| | H | A:VAL  96 | ||| | H | A:VAL  96 | ||| | H | A:VAL  96 | ||| | H | A:VAL  96 | ||| | H | A:VAL  96 | ||| | H | A:VAL  96 | ||| | H | A:VAL  96 |
| H | A:ASN  97 | ||| | H | A:ASN  97 | ||| | H | A:ASN  97 | ||| | H | A:ASN  97 | ||| | H | A:ASN  97 | ||| | H | A:ASN  97 | ||| | H | A:ASN  97 | ||| | H | A:ASN  97 | ||| | H | A:ASN  97 | ||| | H | A:ASN  97 |
| H | A:PHE  98 | ||| | H | A:PHE  98 | ||| | H | A:PHE  98 | ||| | H | A:PHE  98 | ||| | H | A:PHE  98 | ||| | H | A:PHE  98 | ||| | H | A:PHE  98 | ||| | H | A:PHE  98 | ||| | H | A:PHE  98 | ||| | H | A:PHE  98 |
| H | A:LYS  99 | ||| | H | A:LYS  99 | ||| | H | A:LYS  99 | ||| | H | A:LYS  99 | ||| | H | A:LYS  99 | ||| | H | A:LYS  99 | ||| | H | A:LYS  99 | ||| | H | A:LYS  99 | ||| | H | A:LYS  99 | ||| | H | A:LYS  99 |
| H | A:LEU 100 | ||| | H | A:LEU 100 | ||| | H | A:LEU 100 | ||| | H | A:LEU 100 | ||| | H | A:LEU 100 | ||| | H | A:LEU 100 | ||| | H | A:LEU 100 | ||| | H | A:LEU 100 | ||| | H | A:LEU 100 | ||| | H | A:LEU 100 |
| H | A:LEU 101 | ||| | H | A:LEU 101 | ||| | H | A:LEU 101 | ||| | H | A:LEU 101 | ||| | H | A:LEU 101 | ||| | H | A:LEU 101 | ||| | H | A:LEU 101 | ||| | H | A:LEU 101 | ||| | H | A:LEU 101 | ||| | H | A:LEU 101 |
| H | A:SER 102 | ||| | H | A:SER 102 | ||| | H | A:SER 102 | ||| | H | A:SER 102 | ||| | H | A:SER 102 | ||| | H | A:SER 102 | ||| | H | A:SER 102 | ||| | H | A:SER 102 | ||| | H | A:SER 102 | ||| | H | A:SER 102 |
| H | A:HIS 103 | ||| | H | A:HIS 103 | ||| | H | A:HIS 103 | ||| | H | A:HIS 103 | ||| | H | A:HIS 103 | ||| | H | A:HIS 103 | ||| | H | A:HIS 103 | ||| | H | A:HIS 103 | ||| | H | A:HIS 103 | ||| | H | A:HIS 103 |
| H | A:CYS 104 | ||| | H | A:CYS 104 | ||| | H | A:CYS 104 | ||| | H | A:CYS 104 | ||| | H | A:CYS 104 | ||| | H | A:CYS 104 | ||| | H | A:CYS 104 | ||| | H | A:CYS 104 | ||| | H | A:CYS 104 | ||| | H | A:CYS 104 |
| H | A:LEU 105 | ||| | H | A:LEU 105 | ||| | H | A:LEU 105 | ||| | H | A:LEU 105 | ||| | H | A:LEU 105 | ||| | H | A:LEU 105 | ||| | H | A:LEU 105 | ||| | H | A:LEU 105 | ||| | H | A:LEU 105 | ||| | H | A:LEU 105 |
| H | A:LEU 106 | ||| | H | A:LEU 106 | ||| | H | A:LEU 106 | ||| | H | A:LEU 106 | ||| | H | A:LEU 106 | ||| | H | A:LEU 106 | ||| | H | A:LEU 106 | ||| | H | A:LEU 106 | ||| | H | A:LEU 106 | ||| | H | A:LEU 106 |
| H | A:VAL 107 | ||| | H | A:VAL 107 | ||| | H | A:VAL 107 | ||| | H | A:VAL 107 | ||| | H | A:VAL 107 | ||| | H | A:VAL 107 | ||| | H | A:VAL 107 | ||| | H | A:VAL 107 | ||| | H | A:VAL 107 | ||| | H | A:VAL 107 |
| H | A:THR 108 | ||| | H | A:THR 108 | ||| | H | A:THR 108 | ||| | H | A:THR 108 | ||| | H | A:THR 108 | ||| | H | A:THR 108 | ||| | H | A:THR 108 | ||| | H | A:THR 108 | ||| | H | A:THR 108 | ||| | H | A:THR 108 |
| H | A:LEU 109 | ||| | H | A:LEU 109 | ||| | H | A:LEU 109 | ||| | H | A:LEU 109 | ||| | H | A:LEU 109 | ||| | H | A:LEU 109 | ||| | H | A:LEU 109 | ||| | H | A:LEU 109 | ||| | H | A:LEU 109 | ||| | H | A:LEU 109 |
| H | A:ALA 110 | ||| | H | A:ALA 110 | ||| | H | A:ALA 110 | ||| | H | A:ALA 110 | ||| | H | A:ALA 110 | ||| | H | A:ALA 110 | ||| | H | A:ALA 110 | ||| | H | A:ALA 110 | ||| | H | A:ALA 110 | ||| | H | A:ALA 110 |
| H | A:ALA 111 | ||| | H | A:ALA 111 | ||| | H | A:ALA 111 | ||| | H | A:ALA 111 | ||| | H | A:ALA 111 | ||| | H | A:ALA 111 | ||| | H | A:ALA 111 | ||| | H | A:ALA 111 | ||| | H | A:ALA 111 | ||| | H | A:ALA 111 |
| H | A:HIS 112 | ||| | H | A:HIS 112 | ||| | H | A:HIS 112 | ||| | H | A:HIS 112 | ||| | H | A:HIS 112 | ||| | H | A:HIS 112 | ||| | H | A:HIS 112 | ||| | H | A:HIS 112 | ||| | H | A:HIS 112 | ||| | H | A:HIS 112 |
| H | A:LEU 113 | ||| | H | A:LEU 113 | ||| | H | A:LEU 113 | ||| | H | A:LEU 113 | ||| | H | A:LEU 113 | ||| | H | A:LEU 113 | ||| | H | A:LEU 113 | ||| | H | A:LEU 113 | ||| | H | A:LEU 113 | ||| | H | A:LEU 113 |
|  | A:PRO 114 | ||| |  | A:PRO 114 | ||| |  | A:PRO 114 | ||| |  | A:PRO 114 | ||| |  | A:PRO 114 | ||| |  | A:PRO 114 | ||| |  | A:PRO 114 | ||| |  | A:PRO 114 | ||| |  | A:PRO 114 | ||| |  | A:PRO 114 |
|  | A:ALA 115 | ||| |  | A:ALA 115 | ||| |  | A:ALA 115 | ||| |  | A:ALA 115 | ||| |  | A:ALA 115 | ||| |  | A:ALA 115 | ||| |  | A:ALA 115 | ||| |  | A:ALA 115 | ||| |  | A:ALA 115 | ||| |  | A:ALA 115 |
|  | A:GLU 116 | ||| |  | A:GLU 116 | ||| |  | A:GLU 116 | ||| |  | A:GLU 116 | ||| |  | A:GLU 116 | ||| |  | A:GLU 116 | ||| |  | A:GLU 116 | ||| |  | A:GLU 116 | ||| |  | A:GLU 116 | ||| |  | A:GLU 116 |
|  | A:PHE 117 | ||| |  | A:PHE 117 | ||| |  | A:PHE 117 | ||| |  | A:PHE 117 | ||| |  | A:PHE 117 | ||| |  | A:PHE 117 | ||| |  | A:PHE 117 | ||| |  | A:PHE 117 | ||| |  | A:PHE 117 | ||| |  | A:PHE 117 |
| H | A:THR 118 | ||| | H | A:THR 118 | ||| | H | A:THR 118 | ||| | H | A:THR 118 | ||| | H | A:THR 118 | ||| | H | A:THR 118 | ||| | H | A:THR 118 | ||| | H | A:THR 118 | ||| | H | A:THR 118 | ||| | H | A:THR 118 |
| H | A:PRO 119 | ||| | H | A:PRO 119 | ||| | H | A:PRO 119 | ||| | H | A:PRO 119 | ||| | H | A:PRO 119 | ||| | H | A:PRO 119 | ||| | H | A:PRO 119 | ||| | H | A:PRO 119 | ||| | H | A:PRO 119 | ||| | H | A:PRO 119 |
| H | A:ALA 120 | ||| | H | A:ALA 120 | ||| | H | A:ALA 120 | ||| | H | A:ALA 120 | ||| | H | A:ALA 120 | ||| | H | A:ALA 120 | ||| | H | A:ALA 120 | ||| | H | A:ALA 120 | ||| | H | A:ALA 120 | ||| | H | A:ALA 120 |
| H | A:VAL 121 | ||| | H | A:VAL 121 | ||| | H | A:VAL 121 | ||| | H | A:VAL 121 | ||| | H | A:VAL 121 | ||| | H | A:VAL 121 | ||| | H | A:VAL 121 | ||| | H | A:VAL 121 | ||| | H | A:VAL 121 | ||| | H | A:VAL 121 |
| H | A:HIS 122 | ||| | H | A:HIS 122 | ||| | H | A:HIS 122 | ||| | H | A:HIS 122 | ||| | H | A:HIS 122 | ||| | H | A:HIS 122 | ||| | H | A:HIS 122 | ||| | H | A:HIS 122 | ||| | H | A:HIS 122 | ||| | H | A:HIS 122 |
| H | A:ALA 123 | ||| | H | A:ALA 123 | ||| | H | A:ALA 123 | ||| | H | A:ALA 123 | ||| | H | A:ALA 123 | ||| | H | A:ALA 123 | ||| | H | A:ALA 123 | ||| | H | A:ALA 123 | ||| | H | A:ALA 123 | ||| | H | A:ALA 123 |
| H | A:SER 124 | ||| | H | A:SER 124 | ||| | H | A:SER 124 | ||| | H | A:SER 124 | ||| | H | A:SER 124 | ||| | H | A:SER 124 | ||| | H | A:SER 124 | ||| | H | A:SER 124 | ||| | H | A:SER 124 | ||| | H | A:SER 124 |
| H | A:LEU 125 | ||| | H | A:LEU 125 | ||| | H | A:LEU 125 | ||| | H | A:LEU 125 | ||| | H | A:LEU 125 | ||| | H | A:LEU 125 | ||| | H | A:LEU 125 | ||| | H | A:LEU 125 | ||| | H | A:LEU 125 | ||| | H | A:LEU 125 |
| H | A:ASP 126 | ||| | H | A:ASP 126 | ||| | H | A:ASP 126 | ||| | H | A:ASP 126 | ||| | H | A:ASP 126 | ||| | H | A:ASP 126 | ||| | H | A:ASP 126 | ||| | H | A:ASP 126 | ||| | H | A:ASP 126 | ||| | H | A:ASP 126 |
| H | A:LYS 127 | ||| | H | A:LYS 127 | ||| | H | A:LYS 127 | ||| | H | A:LYS 127 | ||| | H | A:LYS 127 | ||| | H | A:LYS 127 | ||| | H | A:LYS 127 | ||| | H | A:LYS 127 | ||| | H | A:LYS 127 | ||| | H | A:LYS 127 |
| H | A:PHE 128 | ||| | H | A:PHE 128 | ||| | H | A:PHE 128 | ||| | H | A:PHE 128 | ||| | H | A:PHE 128 | ||| | H | A:PHE 128 | ||| | H | A:PHE 128 | ||| | H | A:PHE 128 | ||| | H | A:PHE 128 | ||| | H | A:PHE 128 |
| H | A:LEU 129 | ||| | H | A:LEU 129 | ||| | H | A:LEU 129 | ||| | H | A:LEU 129 | ||| | H | A:LEU 129 | ||| | H | A:LEU 129 | ||| | H | A:LEU 129 | ||| | H | A:LEU 129 | ||| | H | A:LEU 129 | ||| | H | A:LEU 129 |
| H | A:ALA 130 | ||| | H | A:ALA 130 | ||| | H | A:ALA 130 | ||| | H | A:ALA 130 | ||| | H | A:ALA 130 | ||| | H | A:ALA 130 | ||| | H | A:ALA 130 | ||| | H | A:ALA 130 | ||| | H | A:ALA 130 | ||| | H | A:ALA 130 |
| H | A:SER 131 | ||| | H | A:SER 131 | ||| | H | A:SER 131 | ||| | H | A:SER 131 | ||| | H | A:SER 131 | ||| | H | A:SER 131 | ||| | H | A:SER 131 | ||| | H | A:SER 131 | ||| | H | A:SER 131 | ||| | H | A:SER 131 |
| H | A:VAL 132 | ||| | H | A:VAL 132 | ||| | H | A:VAL 132 | ||| | H | A:VAL 132 | ||| | H | A:VAL 132 | ||| | H | A:VAL 132 | ||| | H | A:VAL 132 | ||| | H | A:VAL 132 | ||| | H | A:VAL 132 | ||| | H | A:VAL 132 |
| H | A:SER 133 | ||| | H | A:SER 133 | ||| | H | A:SER 133 | ||| | H | A:SER 133 | ||| | H | A:SER 133 | ||| | H | A:SER 133 | ||| | H | A:SER 133 | ||| | H | A:SER 133 | ||| | H | A:SER 133 | ||| | H | A:SER 133 |
| H | A:THR 134 | ||| | H | A:THR 134 | ||| | H | A:THR 134 | ||| | H | A:THR 134 | ||| | H | A:THR 134 | ||| | H | A:THR 134 | ||| | H | A:THR 134 | ||| | H | A:THR 134 | ||| | H | A:THR 134 | ||| | H | A:THR 134 |
| H | A:VAL 135 | ||| | H | A:VAL 135 | ||| | H | A:VAL 135 | ||| | H | A:VAL 135 | ||| | H | A:VAL 135 | ||| | H | A:VAL 135 | ||| | H | A:VAL 135 | ||| | H | A:VAL 135 | ||| | H | A:VAL 135 | ||| | H | A:VAL 135 |
| H | A:LEU 136 | ||| | H | A:LEU 136 | ||| | H | A:LEU 136 | ||| | H | A:LEU 136 | ||| | H | A:LEU 136 | ||| | H | A:LEU 136 | ||| | H | A:LEU 136 | ||| | H | A:LEU 136 | ||| | H | A:LEU 136 | ||| | H | A:LEU 136 |
| H | A:THR 137 | ||| | H | A:THR 137 | ||| | H | A:THR 137 | ||| | H | A:THR 137 | ||| | H | A:THR 137 | ||| | H | A:THR 137 | ||| | H | A:THR 137 | ||| | H | A:THR 137 | ||| | H | A:THR 137 | ||| | H | A:THR 137 |
| H | A:SER 138 | ||| | H | A:SER 138 | ||| | H | A:SER 138 | ||| | H | A:SER 138 | ||| |  | A:SER 138 | ||| | H | A:SER 138 | ||| |  | A:SER 138 | ||| | H | A:SER 138 | ||| |  | A:SER 138 | ||| |  | A:SER 138 |
|  | A:LYS 139 | ||| |  | A:LYS 139 | ||| |  | A:LYS 139 | ||| |  | A:LYS 139 | ||| |  | A:LYS 139 | ||| |  | A:LYS 139 | ||| |  | A:LYS 139 | ||| |  | A:LYS 139 | ||| |  | A:LYS 139 | ||| |  | A:LYS 139 |
|  | A:TYR 140 | ||| |  | A:TYR 140 | ||| |  | A:TYR 140 | ||| |  | A:TYR 140 | ||| |  | A:TYR 140 | ||| |  | A:TYR 140 | ||| |  | A:TYR 140 | ||| |  | A:TYR 140 | ||| |  | A:TYR 140 | ||| |  | A:TYR 140 |
|  | A:ARG 141 |  |  | |  |  | |  |  | |  |  | |  |  | |  |  | |  |  | |  |  | |  |  | |
|  | B:VAL   1 | ||| |  | A:ARG 141 | ||| |  | A:ARG 141 | ||| |  | A:ARG 141 | ||| |  | A:ARG 141 | ||| |  | A:ARG 141 | ||| |  | A:ARG 141 | ||| |  | A:ARG 141 | ||| |  | A:ARG 141 | ||| |  | A:ARG 141 |
|  | B:HIS   2 | ||| |  | C:VAL   1 | ||| |  | B:MET   1 | ||| |  | C:VAL   1 | ||| |  | B:MET   1 | ||| |  | C:VAL   1 | ||| |  | B:VAL   1 | ||| |  | C:VAL   1 | ||| |  | B:MET   1 | ||| |  | B:MET   1 |
|  | B:LEU   3 | ||| |  | C:LEU   2 | ||| |  | B:HIS   2 | ||| |  | C:LEU   2 | ||| |  | B:HIS   2 | ||| |  | C:LEU   2 | ||| |  | B:HIS   2 | ||| |  | C:LEU   2 | ||| |  | B:HIS   2 | ||| |  | B:HIS   2 |
| H | B:THR   4 | ||| | H | C:SER   3 | ||| |  | B:LEU   3 | ||| | H | C:SER   3 | ||| |  | B:LEU   3 | ||| | H | C:SER   3 | ||| |  | B:LEU   3 | ||| | H | C:SER   3 | ||| |  | B:LEU   3 | ||| |  | B:LEU   3 |
| H | B:PRO   5 | ||| | H | C:PRO   4 | ||| | H | B:THR   4 | ||| | H | C:PRO   4 | ||| | H | B:THR   4 | ||| | H | C:PRO   4 | ||| | H | B:THR   4 | ||| | H | C:PRO   4 | ||| | H | B:THR   4 | ||| | H | B:THR   4 |
| H | B:GLU   6 | ||| | H | C:ALA   5 | ||| | H | B:PRO   5 | ||| | H | C:ALA   5 | ||| | H | B:PRO   5 | ||| | H | C:ALA   5 | ||| | H | B:PRO   5 | ||| | H | C:ALA   5 | ||| | H | B:PRO   5 | ||| | H | B:PRO   5 |
| H | B:GLU   7 | ||| | H | C:ASP   6 | ||| | H | B:GLU   6 | ||| | H | C:ASP   6 | ||| | H | B:GLU   6 | ||| | H | C:ASP   6 | ||| | H | B:GLU   6 | ||| | H | C:ASP   6 | ||| | H | B:GLU   6 | ||| | H | B:GLU   6 |
| H | B:LYS   8 | ||| | H | C:LYS   7 | ||| | H | B:GLU   7 | ||| | H | C:LYS   7 | ||| | H | B:GLU   7 | ||| | H | C:LYS   7 | ||| | H | B:GLU   7 | ||| | H | C:LYS   7 | ||| | H | B:GLU   7 | ||| | H | B:GLU   7 |
| H | B:SER   9 | ||| | H | C:THR   8 | ||| | H | B:LYS   8 | ||| | H | C:THR   8 | ||| | H | B:LYS   8 | ||| | H | C:THR   8 | ||| | H | B:LYS   8 | ||| | H | C:THR   8 | ||| | H | B:LYS   8 | ||| | H | B:LYS   8 |
| H | B:ALA  10 | ||| | H | C:ASN   9 | ||| | H | B:SER   9 | ||| | H | C:ASN   9 | ||| | H | B:SER   9 | ||| | H | C:ASN   9 | ||| | H | B:SER   9 | ||| | H | C:ASN   9 | ||| | H | B:SER   9 | ||| | H | B:SER   9 |
| H | B:VAL  11 | ||| | H | C:VAL  10 | ||| | H | B:ALA  10 | ||| | H | C:VAL  10 | ||| | H | B:ALA  10 | ||| | H | C:VAL  10 | ||| | H | B:ALA  10 | ||| | H | C:VAL  10 | ||| | H | B:ALA  10 | ||| | H | B:ALA  10 |
| H | B:THR  12 | ||| | H | C:LYS  11 | ||| | H | B:VAL  11 | ||| | H | C:LYS  11 | ||| | H | B:VAL  11 | ||| | H | C:LYS  11 | ||| | H | B:VAL  11 | ||| | H | C:LYS  11 | ||| | H | B:VAL  11 | ||| | H | B:VAL  11 |
| H | B:ALA  13 | ||| | H | C:ALA  12 | ||| | H | B:THR  12 | ||| | H | C:ALA  12 | ||| | H | B:THR  12 | ||| | H | C:ALA  12 | ||| | H | B:THR  12 | ||| | H | C:ALA  12 | ||| | H | B:THR  12 | ||| | H | B:THR  12 |
| H | B:LEU  14 | ||| | H | C:ALA  13 | ||| | H | B:ALA  13 | ||| | H | C:ALA  13 | ||| | H | B:ALA  13 | ||| | H | C:ALA  13 | ||| | H | B:ALA  13 | ||| | H | C:ALA  13 | ||| | H | B:ALA  13 | ||| | H | B:ALA  13 |
| H | B:TRP  15 | ||| | H | C:TRP  14 | ||| | H | B:LEU  14 | ||| | H | C:TRP  14 | ||| | H | B:LEU  14 | ||| | H | C:TRP  14 | ||| | H | B:LEU  14 | ||| | H | C:TRP  14 | ||| | H | B:LEU  14 | ||| | H | B:LEU  14 |
|  | |  | H | C:GLY  15 |  |  | |  | H | C:GLY  15 |  |  | |  | H | C:GLY  15 |  |  | |  | H | C:GLY  15 |  |  | |  |  | |
|  | |  | H | C:LYS  16 |  |  | |  | H | C:LYS  16 |  |  | |  | H | C:LYS  16 |  |  | |  | H | C:LYS  16 |  |  | |  |  | |
|  | |  | H | C:VAL  17 |  |  | |  | H | C:VAL  17 |  |  | |  | H | C:VAL  17 |  |  | |  | H | C:VAL  17 |  |  | |  |  | |
|  | |  | H | C:GLY  18 |  |  | |  | H | C:GLY  18 |  |  | |  | H | C:GLY  18 |  |  | |  | H | C:GLY  18 |  |  | |  |  | |
| H | B:GLY  16 | ||| |  | C:ALA  19 | ||| | H | B:TRP  15 | ||| |  | C:ALA  19 | ||| | H | B:TRP  15 | ||| |  | C:ALA  19 | ||| | H | B:TRP  15 | ||| |  | C:ALA  19 | ||| | H | B:TRP  15 | ||| | H | B:TRP  15 |
|  | B:LYS  17 | ||| | H | C:HIS  20 | ||| | H | B:GLY  16 | ||| | H | C:HIS  20 | ||| | H | B:GLY  16 | ||| | H | C:HIS  20 | ||| | H | B:GLY  16 | ||| | H | C:HIS  20 | ||| | H | B:GLY  16 | ||| | H | B:GLY  16 |
|  | B:VAL  18 | ||| | H | C:ALA  21 | ||| |  | B:LYS  17 | ||| | H | C:ALA  21 | ||| |  | B:LYS  17 | ||| | H | C:ALA  21 | ||| |  | B:LYS  17 | ||| | H | C:ALA  21 | ||| |  | B:LYS  17 | ||| |  | B:LYS  17 |
|  | B:ASN  19 |  |  | |  |  | |  |  | |  |  | |  |  | |  |  | |  |  | |  |  | |  |  | |
|  | B:VAL  20 | ||| | H | C:GLY  22 | ||| |  | B:VAL  18 | ||| | H | C:GLY  22 | ||| |  | B:VAL  18 | ||| | H | C:GLY  22 | ||| |  | B:VAL  18 | ||| | H | C:GLY  22 | ||| |  | B:VAL  18 | ||| |  | B:VAL  18 |
|  | B:ASP  21 | ||| | H | C:GLU  23 | ||| | H | B:ASN  19 | ||| | H | C:GLU  23 | ||| | H | B:ASN  19 | ||| | H | C:GLU  23 | ||| | H | B:ASN  19 | ||| | H | C:GLU  23 | ||| | H | B:ASN  19 | ||| | H | B:ASN  19 |
|  | |  |  | |  | H | B:VAL  20 |  |  | |  | H | B:VAL  20 |  |  | |  | H | B:VAL  20 |  |  | |  | H | B:VAL  20 |  | H | B:VAL  20 |
| H | B:GLU  22 | ||| | H | C:TYR  24 | ||| | H | B:ASP  21 | ||| | H | C:TYR  24 | ||| | H | B:ASP  21 | ||| | H | C:TYR  24 | ||| | H | B:ASP  21 | ||| | H | C:TYR  24 | ||| | H | B:ASP  21 | ||| | H | B:ASP  21 |
| H | B:VAL  23 | ||| | H | C:GLY  25 | ||| | H | B:GLU  22 | ||| | H | C:GLY  25 | ||| | H | B:GLU  22 | ||| | H | C:GLY  25 | ||| | H | B:GLU  22 | ||| | H | C:GLY  25 | ||| | H | B:GLU  22 | ||| | H | B:GLU  22 |
| H | B:GLY  24 | ||| | H | C:ALA  26 | ||| | H | B:VAL  23 | ||| | H | C:ALA  26 | ||| | H | B:VAL  23 | ||| | H | C:ALA  26 | ||| | H | B:VAL  23 | ||| | H | C:ALA  26 | ||| | H | B:VAL  23 | ||| | H | B:VAL  23 |
| H | B:GLY  25 | ||| | H | C:GLU  27 | ||| | H | B:GLY  24 | ||| | H | C:GLU  27 | ||| | H | B:GLY  24 | ||| | H | C:GLU  27 | ||| | H | B:GLY  24 | ||| | H | C:GLU  27 | ||| | H | B:GLY  24 | ||| | H | B:GLY  24 |
| H | B:GLU  26 | ||| | H | C:ALA  28 | ||| | H | B:GLY  25 | ||| | H | C:ALA  28 | ||| | H | B:GLY  25 | ||| | H | C:ALA  28 | ||| | H | B:GLY  25 | ||| | H | C:ALA  28 | ||| | H | B:GLY  25 | ||| | H | B:GLY  25 |
| H | B:ALA  27 | ||| | H | C:LEU  29 | ||| | H | B:GLU  26 | ||| | H | C:LEU  29 | ||| | H | B:GLU  26 | ||| | H | C:LEU  29 | ||| | H | B:GLU  26 | ||| | H | C:LEU  29 | ||| | H | B:GLU  26 | ||| | H | B:GLU  26 |
| H | B:LEU  28 | ||| | H | C:GLU  30 | ||| | H | B:ALA  27 | ||| | H | C:GLU  30 | ||| | H | B:ALA  27 | ||| | H | C:GLU  30 | ||| | H | B:ALA  27 | ||| | H | C:GLU  30 | ||| | H | B:ALA  27 | ||| | H | B:ALA  27 |
| H | B:GLY  29 | ||| | H | C:ARG  31 | ||| | H | B:LEU  28 | ||| | H | C:ARG  31 | ||| | H | B:LEU  28 | ||| | H | C:ARG  31 | ||| | H | B:LEU  28 | ||| | H | C:ARG  31 | ||| | H | B:LEU  28 | ||| | H | B:LEU  28 |
| H | B:ARG  30 | ||| | H | C:MET  32 | ||| | H | B:GLY  29 | ||| | H | C:MET  32 | ||| | H | B:GLY  29 | ||| | H | C:MET  32 | ||| | H | B:GLY  29 | ||| | H | C:MET  32 | ||| | H | B:GLY  29 | ||| | H | B:GLY  29 |
| H | B:LEU  31 | ||| | H | C:PHE  33 | ||| | H | B:ARG  30 | ||| | H | C:PHE  33 | ||| | H | B:ARG  30 | ||| | H | C:PHE  33 | ||| | H | B:ARG  30 | ||| | H | C:PHE  33 | ||| | H | B:ARG  30 | ||| | H | B:ARG  30 |
| H | B:LEU  32 | ||| | H | C:LEU  34 | ||| | H | B:LEU  31 | ||| | H | C:LEU  34 | ||| | H | B:LEU  31 | ||| | H | C:LEU  34 | ||| | H | B:LEU  31 | ||| | H | C:LEU  34 | ||| | H | B:LEU  31 | ||| | H | B:LEU  31 |
| H | B:VAL  33 | ||| | H | C:SER  35 | ||| | H | B:LEU  32 | ||| | H | C:SER  35 | ||| | H | B:LEU  32 | ||| | H | C:SER  35 | ||| | H | B:LEU  32 | ||| | H | C:SER  35 | ||| | H | B:LEU  32 | ||| | H | B:LEU  32 |
| H | B:VAL  34 | ||| | H | C:PHE  36 | ||| | H | B:VAL  33 | ||| | H | C:PHE  36 | ||| | H | B:VAL  33 | ||| | H | C:PHE  36 | ||| | H | B:VAL  33 | ||| | H | C:PHE  36 | ||| | H | B:VAL  33 | ||| | H | B:VAL  33 |
| H | B:TYR  35 | ||| | H | C:PRO  37 | ||| | H | B:VAL  34 | ||| | H | C:PRO  37 | ||| | H | B:VAL  34 | ||| | H | C:PRO  37 | ||| | H | B:VAL  34 | ||| | H | C:PRO  37 | ||| | H | B:VAL  34 | ||| | H | B:VAL  34 |
| H | B:PRO  36 | ||| | H | C:THR  38 | ||| | H | B:TYR  35 | ||| | H | C:THR  38 | ||| | H | B:TYR  35 | ||| | H | C:THR  38 | ||| | H | B:TYR  35 | ||| | H | C:THR  38 | ||| | H | B:TYR  35 | ||| | H | B:TYR  35 |
| H | B:TRP  37 | ||| | H | C:THR  39 | ||| | H | B:PRO  36 | ||| | H | C:THR  39 | ||| | H | B:PRO  36 | ||| | H | C:THR  39 | ||| | H | B:PRO  36 | ||| | H | C:THR  39 | ||| | H | B:PRO  36 | ||| | H | B:PRO  36 |
| H | B:THR  38 | ||| | H | C:LYS  40 | ||| | H | B:ALA  37 | ||| | H | C:LYS  40 | ||| | H | B:TYR  37 | ||| | H | C:LYS  40 | ||| | H | B:TRP  37 | ||| | H | C:LYS  40 | ||| | H | B:TRP  37 | ||| | H | B:TRP  37 |
| H | B:GLN  39 | ||| | H | C:THR  41 | ||| | H | B:THR  38 | ||| | H | C:THR  41 | ||| | H | B:THR  38 | ||| | H | C:THR  41 | ||| | H | B:THR  38 | ||| | H | C:THR  41 | ||| | H | B:THR  38 | ||| | H | B:THR  38 |
| H | B:ARG  40 | ||| | H | C:TYR  42 | ||| | H | B:GLN  39 | ||| | H | C:TYR  42 | ||| | H | B:GLN  39 | ||| | H | C:TYR  42 | ||| | H | B:GLN  39 | ||| | H | C:TYR  42 | ||| | H | B:GLN  39 | ||| | H | B:GLN  39 |
| H | B:PHE  41 | ||| | H | C:PHE  43 | ||| | H | B:ARG  40 | ||| | H | C:PHE  43 | ||| | H | B:ARG  40 | ||| | H | C:PHE  43 | ||| | H | B:ARG  40 | ||| | H | C:PHE  43 | ||| | H | B:ARG  40 | ||| | H | B:ARG  40 |
| H | B:PHE  42 | ||| |  | C:PRO  44 | ||| | H | B:PHE  41 | ||| |  | C:PRO  44 | ||| | H | B:PHE  41 | ||| |  | C:PRO  44 | ||| | H | B:PHE  41 | ||| |  | C:PRO  44 | ||| | H | B:PHE  41 | ||| | H | B:PHE  41 |
| H | B:GLU  43 | ||| |  | C:HIS  45 | ||| | H | B:PHE  42 | ||| |  | C:HIS  45 | ||| | H | B:PHE  42 | ||| |  | C:HIS  45 | ||| | H | B:PHE  42 | ||| |  | C:HIS  45 | ||| | H | B:PHE  42 | ||| | H | B:PHE  42 |
| H | B:SER  44 | ||| |  | C:PHE  46 | ||| | H | B:GLU  43 | ||| |  | C:PHE  46 | ||| | H | B:GLU  43 | ||| |  | C:PHE  46 | ||| | H | B:GLU  43 | ||| |  | C:PHE  46 | ||| | H | B:GLU  43 | ||| | H | B:GLU  43 |
| H | B:PHE  45 |  |  | |  |  | |  |  | |  |  | |  |  | |  |  | |  |  | |  |  | |  |  | |
| H | B:GLY  46 | ||| |  | C:ASP  47 | ||| | H | B:SER  44 | ||| |  | C:ASP  47 | ||| | H | B:SER  44 | ||| |  | C:ASP  47 | ||| | H | B:SER  44 | ||| |  | C:ASP  47 | ||| | H | B:SER  44 | ||| | H | B:SER  44 |
|  | B:ASP  47 | ||| |  | C:LEU  48 | ||| | H | B:PHE  45 | ||| |  | C:LEU  48 | ||| | H | B:PHE  45 | ||| |  | C:LEU  48 | ||| | H | B:PHE  45 | ||| |  | C:LEU  48 | ||| | H | B:PHE  45 | ||| | H | B:PHE  45 |
|  | B:LEU  48 | ||| |  | C:SER  49 | ||| | H | B:GLY  46 | ||| |  | C:SER  49 | ||| | H | B:GLY  46 | ||| |  | C:SER  49 | ||| | H | B:GLY  46 | ||| |  | C:SER  49 | ||| | H | B:GLY  46 | ||| | H | B:GLY  46 |
|  | B:SER  49 | ||| |  | C:HIS  50 | ||| |  | B:ASP  47 | ||| |  | C:HIS  50 | ||| |  | B:ASP  47 | ||| |  | C:HIS  50 | ||| |  | B:ASP  47 | ||| |  | C:HIS  50 | ||| |  | B:ASP  47 | ||| |  | B:ASP  47 |
| H | B:THR  50 | ||| |  | C:GLY  51 | ||| |  | B:LEU  48 | ||| |  | C:GLY  51 | ||| |  | B:LEU  48 | ||| |  | C:GLY  51 | ||| |  | B:LEU  48 | ||| |  | C:GLY  51 | ||| |  | B:LEU  48 | ||| |  | B:LEU  48 |
|  | |  |  | |  |  | B:SER  49 |  |  | |  |  | B:SER  49 |  |  | |  |  | B:SER  49 |  |  | |  |  | B:SER  49 |  |  | B:SER  49 |
| H | B:PRO  51 | ||| | H | C:SER  52 | ||| | H | B:THR  50 | ||| | H | C:SER  52 | ||| | H | B:THR  50 | ||| | H | C:SER  52 | ||| | H | B:THR  50 | ||| | H | C:SER  52 | ||| | H | B:THR  50 | ||| | H | B:THR  50 |
| H | B:ASP  52 | ||| | H | C:ALA  53 | ||| | H | B:PRO  51 | ||| | H | C:ALA  53 | ||| | H | B:PRO  51 | ||| | H | C:ALA  53 | ||| | H | B:PRO  51 | ||| | H | C:ALA  53 | ||| | H | B:PRO  51 | ||| | H | B:PRO  51 |
| H | B:ALA  53 | ||| | H | C:GLN  54 | ||| | H | B:ASP  52 | ||| | H | C:GLN  54 | ||| | H | B:ASP  52 | ||| | H | C:GLN  54 | ||| | H | B:ASP  52 | ||| | H | C:GLN  54 | ||| | H | B:ASP  52 | ||| | H | B:ASP  52 |
| H | B:VAL  54 | ||| | H | C:VAL  55 | ||| | H | B:ALA  53 | ||| | H | C:VAL  55 | ||| | H | B:ALA  53 | ||| | H | C:VAL  55 | ||| | H | B:ALA  53 | ||| | H | C:VAL  55 | ||| | H | B:ALA  53 | ||| | H | B:ALA  53 |
| H | B:MET  55 | ||| | H | C:LYS  56 | ||| | H | B:VAL  54 | ||| | H | C:LYS  56 | ||| | H | B:VAL  54 | ||| | H | C:LYS  56 | ||| | H | B:VAL  54 | ||| | H | C:LYS  56 | ||| | H | B:VAL  54 | ||| | H | B:VAL  54 |
| H | B:GLY  56 | ||| | H | C:GLY  57 | ||| | H | B:MET  55 | ||| | H | C:GLY  57 | ||| | H | B:MET  55 | ||| | H | C:GLY  57 | ||| | H | B:MET  55 | ||| | H | C:GLY  57 | ||| | H | B:MET  55 | ||| | H | B:MET  55 |
| H | B:ASN  57 | ||| | H | C:HIS  58 | ||| | H | B:GLY  56 | ||| | H | C:HIS  58 | ||| | H | B:GLY  56 | ||| | H | C:HIS  58 | ||| | H | B:GLY  56 | ||| | H | C:HIS  58 | ||| | H | B:GLY  56 | ||| | H | B:GLY  56 |
|  | |  |  | |  | H | B:ASN  57 |  |  | |  | H | B:ASN  57 |  |  | |  | H | B:ASN  57 |  |  | |  | H | B:ASN  57 |  | H | B:ASN  57 |
|  | |  |  | |  | H | B:PRO  58 |  |  | |  | H | B:PRO  58 |  |  | |  | H | B:PRO  58 |  |  | |  | H | B:PRO  58 |  | H | B:PRO  58 |
|  | |  |  | |  | H | B:LYS  59 |  |  | |  | H | B:LYS  59 |  |  | |  | H | B:LYS  59 |  |  | |  | H | B:LYS  59 |  | H | B:LYS  59 |
| H | B:PRO  58 | ||| | H | C:GLY  59 | ||| | H | B:VAL  60 | ||| | H | C:GLY  59 | ||| | H | B:VAL  60 | ||| | H | C:GLY  59 | ||| | H | B:VAL  60 | ||| | H | C:GLY  59 | ||| | H | B:VAL  60 | ||| | H | B:VAL  60 |
| H | B:LYS  59 | ||| | H | C:LYS  60 | ||| | H | B:LYS  61 | ||| | H | C:LYS  60 | ||| | H | B:LYS  61 | ||| | H | C:LYS  60 | ||| | H | B:LYS  61 | ||| | H | C:LYS  60 | ||| | H | B:LYS  61 | ||| | H | B:LYS  61 |
| H | B:VAL  60 | ||| | H | C:LYS  61 | ||| | H | B:ALA  62 | ||| | H | C:LYS  61 | ||| | H | B:ALA  62 | ||| | H | C:LYS  61 | ||| | H | B:ALA  62 | ||| | H | C:LYS  61 | ||| | H | B:ALA  62 | ||| | H | B:ALA  62 |
| H | B:LYS  61 | ||| | H | C:VAL  62 | ||| | H | B:HIS  63 | ||| | H | C:VAL  62 | ||| | H | B:HIS  63 | ||| | H | C:VAL  62 | ||| | H | B:HIS  63 | ||| | H | C:VAL  62 | ||| | H | B:HIS  63 | ||| | H | B:HIS  63 |
| H | B:ALA  62 | ||| | H | C:ALA  63 | ||| | H | B:GLY  64 | ||| | H | C:ALA  63 | ||| | H | B:GLY  64 | ||| | H | C:ALA  63 | ||| | H | B:GLY  64 | ||| | H | C:ALA  63 | ||| | H | B:GLY  64 | ||| | H | B:GLY  64 |
| H | B:HIS  63 | ||| | H | C:ASP  64 | ||| | H | B:LYS  65 | ||| | H | C:ASP  64 | ||| | H | B:LYS  65 | ||| | H | C:ASP  64 | ||| | H | B:LYS  65 | ||| | H | C:ASP  64 | ||| | H | B:LYS  65 | ||| | H | B:LYS  65 |
| H | B:GLY  64 | ||| | H | C:ALA  65 | ||| | H | B:LYS  66 | ||| | H | C:ALA  65 | ||| | H | B:LYS  66 | ||| | H | C:ALA  65 | ||| | H | B:LYS  66 | ||| | H | C:ALA  65 | ||| | H | B:LYS  66 | ||| | H | B:LYS  66 |
| H | B:LYS  65 | ||| | H | C:LEU  66 | ||| | H | B:VAL  67 | ||| | H | C:LEU  66 | ||| | H | B:VAL  67 | ||| | H | C:LEU  66 | ||| | H | B:VAL  67 | ||| | H | C:LEU  66 | ||| | H | B:VAL  67 | ||| | H | B:VAL  67 |
| H | B:LYS  66 | ||| | H | C:THR  67 | ||| | H | B:LEU  68 | ||| | H | C:THR  67 | ||| | H | B:LEU  68 | ||| | H | C:THR  67 | ||| | H | B:LEU  68 | ||| | H | C:THR  67 | ||| | H | B:LEU  68 | ||| | H | B:LEU  68 |
| H | B:VAL  67 | ||| | H | C:ASN  68 | ||| | H | B:GLY  69 | ||| | H | C:ASN  68 | ||| | H | B:GLY  69 | ||| | H | C:ASN  68 | ||| | H | B:GLY  69 | ||| | H | C:ASN  68 | ||| | H | B:GLY  69 | ||| | H | B:GLY  69 |
| H | B:LEU  68 | ||| | H | C:ALA  69 | ||| | H | B:ALA  70 | ||| | H | C:ALA  69 | ||| | H | B:ALA  70 | ||| | H | C:ALA  69 | ||| | H | B:ALA  70 | ||| | H | C:ALA  69 | ||| | H | B:ALA  70 | ||| | H | B:ALA  70 |
| H | B:GLY  69 | ||| | H | C:VAL  70 | ||| | H | B:PHE  71 | ||| | H | C:VAL  70 | ||| | H | B:PHE  71 | ||| | H | C:VAL  70 | ||| | H | B:PHE  71 | ||| | H | C:VAL  70 | ||| | H | B:PHE  71 | ||| | H | B:PHE  71 |
| H | B:ALA  70 | ||| | H | C:ALA  71 | ||| | H | B:SER  72 | ||| | H | C:ALA  71 | ||| | H | B:SER  72 | ||| | H | C:ALA  71 | ||| | H | B:SER  72 | ||| | H | C:ALA  71 | ||| | H | B:SER  72 | ||| | H | B:SER  72 |
| H | B:PHE  71 | ||| | H | C:HIS  72 | ||| | H | B:ASP  73 | ||| | H | C:HIS  72 | ||| | H | B:ASP  73 | ||| | H | C:HIS  72 | ||| | H | B:ASP  73 | ||| | H | C:HIS  72 | ||| | H | B:ASP  73 | ||| | H | B:ASP  73 |
| H | B:SER  72 | ||| |  | C:VAL  73 | ||| | H | B:GLY  74 | ||| |  | C:VAL  73 | ||| | H | B:GLY  74 | ||| |  | C:VAL  73 | ||| | H | B:GLY  74 | ||| |  | C:VAL  73 | ||| | H | B:GLY  74 | ||| | H | B:GLY  74 |
| H | B:ASP  73 | ||| |  | C:ASP  74 | ||| | H | B:LEU  75 | ||| |  | C:ASP  74 | ||| | H | B:LEU  75 | ||| |  | C:ASP  74 | ||| | H | B:LEU  75 | ||| |  | C:ASP  74 | ||| | H | B:LEU  75 | ||| | H | B:LEU  75 |
| H | B:GLY  74 |  |  | |  |  | |  |  | |  |  | |  |  | |  |  | |  |  | |  |  | |  |  | |
| H | B:LEU  75 |  |  | |  |  | |  |  | |  |  | |  |  | |  |  | |  |  | |  |  | |  |  | |
| H | B:ALA  76 |  |  | |  |  | |  |  | |  |  | |  |  | |  |  | |  |  | |  |  | |  |  | |
| H | B:HIS  77 |  |  | |  |  | |  |  | |  |  | |  |  | |  |  | |  |  | |  |  | |  |  | |
|  | B:LEU  78 |  |  | |  |  | |  |  | |  |  | |  |  | |  |  | |  |  | |  |  | |  |  | |
|  | B:ASP  79 |  |  | |  |  | |  |  | |  |  | |  |  | |  |  | |  |  | |  |  | |  |  | |
| H | B:ASN  80 |  |  | |  |  | |  |  | |  |  | |  |  | |  |  | |  |  | |  |  | |  |  | |
| H | B:LEU  81 | ||| | H | C:ASP  75 | ||| |  | B:ALA  76 | ||| | H | C:ASP  75 | ||| | H | B:ALA  76 | ||| | H | C:ASP  75 | ||| | H | B:ALA  76 | ||| | H | C:ASP  75 | ||| |  | B:ALA  76 | ||| | H | B:ALA  76 |
| H | B:LYS  82 | ||| | H | C:MET  76 | ||| |  | B:HIS  77 | ||| | H | C:MET  76 | ||| |  | B:HIS  77 | ||| | H | C:MET  76 | ||| |  | B:HIS  77 | ||| | H | C:MET  76 | ||| |  | B:HIS  77 | ||| |  | B:HIS  77 |
| H | B:GLY  83 | ||| | H | C:PRO  77 | ||| |  | B:LEU  78 | ||| | H | C:PRO  77 | ||| |  | B:LEU  78 | ||| | H | C:PRO  77 | ||| |  | B:LEU  78 | ||| | H | C:PRO  77 | ||| |  | B:LEU  78 | ||| |  | B:LEU  78 |
| H | B:THR  84 | ||| | H | C:ASN  78 | ||| |  | B:ASP  79 | ||| | H | C:ASN  78 | ||| |  | B:ASP  79 | ||| | H | C:ASN  78 | ||| |  | B:ASP  79 | ||| | H | C:ASN  78 | ||| |  | B:ASP  79 | ||| |  | B:ASP  79 |
| H | B:PHE  85 | ||| | H | C:ALA  79 | ||| | H | B:ASN  80 | ||| | H | C:ALA  79 | ||| | H | B:ASN  80 | ||| | H | C:ALA  79 | ||| | H | B:ASN  80 | ||| | H | C:ALA  79 | ||| | H | B:ASN  80 | ||| | H | B:ASN  80 |
|  | |  |  | |  | H | B:LEU  81 |  |  | |  | H | B:LEU  81 |  |  | |  | H | B:LEU  81 |  |  | |  | H | B:LEU  81 |  | H | B:LEU  81 |
| H | B:ALA  86 | ||| | H | C:LEU  80 | ||| | H | B:LYS  82 | ||| | H | C:LEU  80 | ||| | H | B:LYS  82 | ||| | H | C:LEU  80 | ||| | H | B:LYS  82 | ||| | H | C:LEU  80 | ||| | H | B:LYS  82 | ||| | H | B:LYS  82 |
| H | B:THR  87 | ||| | H | C:SER  81 | ||| | H | B:GLY  83 | ||| | H | C:SER  81 | ||| | H | B:GLY  83 | ||| | H | C:SER  81 | ||| | H | B:GLY  83 | ||| | H | C:SER  81 | ||| | H | B:GLY  83 | ||| | H | B:GLY  83 |
| H | B:LEU  88 | ||| | H | C:ALA  82 | ||| | H | B:THR  84 | ||| | H | C:ALA  82 | ||| | H | B:THR  84 | ||| | H | C:ALA  82 | ||| | H | B:THR  84 | ||| | H | C:ALA  82 | ||| | H | B:THR  84 | ||| | H | B:THR  84 |
| H | B:SER  89 | ||| | H | C:LEU  83 | ||| | H | B:PHE  85 | ||| | H | C:LEU  83 | ||| | H | B:PHE  85 | ||| | H | C:LEU  83 | ||| | H | B:PHE  85 | ||| | H | C:LEU  83 | ||| | H | B:PHE  85 | ||| | H | B:PHE  85 |
| H | B:GLU  90 | ||| | H | C:SER  84 | ||| | H | B:ALA  86 | ||| | H | C:SER  84 | ||| | H | B:ALA  86 | ||| | H | C:SER  84 | ||| | H | B:ALA  86 | ||| | H | C:SER  84 | ||| | H | B:ALA  86 | ||| | H | B:ALA  86 |
| H | B:LEU  91 | ||| | H | C:ASP  85 | ||| | H | B:THR  87 | ||| | H | C:ASP  85 | ||| | H | B:THR  87 | ||| | H | C:ASP  85 | ||| | H | B:THR  87 | ||| | H | C:ASP  85 | ||| | H | B:THR  87 | ||| | H | B:THR  87 |
| H | B:HIS  92 | ||| | H | C:LEU  86 | ||| | H | B:LEU  88 | ||| | H | C:LEU  86 | ||| | H | B:LEU  88 | ||| | H | C:LEU  86 | ||| | H | B:LEU  88 | ||| | H | C:LEU  86 | ||| | H | B:LEU  88 | ||| | H | B:LEU  88 |
| H | B:SNC  93 | ||| | H | C:HIS  87 | ||| | H | B:SER  89 | ||| | H | C:HIS  87 | ||| | H | B:SER  89 | ||| | H | C:HIS  87 | ||| | H | B:SER  89 | ||| | H | C:HIS  87 | ||| | H | B:SER  89 | ||| | H | B:SER  89 |
| H | B:ASP  94 | ||| | H | C:ALA  88 | ||| | H | B:GLU  90 | ||| | H | C:ALA  88 | ||| | H | B:GLU  90 | ||| | H | C:ALA  88 | ||| | H | B:GLU  90 | ||| | H | C:ALA  88 | ||| | H | B:GLU  90 | ||| | H | B:GLU  90 |
| H | B:LYS  95 | ||| | H | C:HIS  89 | ||| | H | B:LEU  91 | ||| | H | C:HIS  89 | ||| | H | B:LEU  91 | ||| | H | C:HIS  89 | ||| | H | B:LEU  91 | ||| | H | C:HIS  89 | ||| | H | B:LEU  91 | ||| | H | B:LEU  91 |
|  | B:LEU  96 | ||| | H | C:LYS  90 | ||| | H | B:HIS  92 | ||| | H | C:LYS  90 | ||| | H | B:HIS  92 | ||| | H | C:LYS  90 | ||| | H | B:HIS  92 | ||| | H | C:LYS  90 | ||| | H | B:HIS  92 | ||| | H | B:HIS  92 |
|  | B:HIS  97 | ||| |  | C:LEU  91 | ||| | H | B:CYS  93 | ||| |  | C:LEU  91 | ||| | H | B:CYS  93 | ||| |  | C:LEU  91 | ||| | H | B:CYS  93 | ||| |  | C:LEU  91 | ||| | H | B:CYS  93 | ||| | H | B:CYS  93 |
|  | |  |  | |  | H | B:ASP  94 |  |  | |  | H | B:ASP  94 |  |  | |  | H | B:ASP  94 |  |  | |  | H | B:ASP  94 |  | H | B:ASP  94 |
|  | |  |  | |  | H | B:LYS  95 |  |  | |  | H | B:LYS  95 |  |  | |  | H | B:LYS  95 |  |  | |  | H | B:LYS  95 |  | H | B:LYS  95 |
|  | |  |  | |  |  | B:LEU  96 |  |  | |  |  | B:LEU  96 |  |  | |  |  | B:LEU  96 |  |  | |  |  | B:LEU  96 |  |  | B:LEU  96 |
|  | B:VAL  98 | ||| |  | C:ARG  92 | ||| |  | B:HIS  97 | ||| |  | C:ARG  92 | ||| |  | B:HIS  97 | ||| |  | C:ARG  92 | ||| |  | B:HIS  97 | ||| |  | C:ARG  92 | ||| |  | B:HIS  97 | ||| |  | B:HIS  97 |
|  | B:ASP  99 | ||| |  | C:VAL  93 | ||| |  | B:VAL  98 | ||| |  | C:VAL  93 | ||| |  | B:VAL  98 | ||| |  | C:VAL  93 | ||| |  | B:VAL  98 | ||| |  | C:VAL  93 | ||| |  | B:VAL  98 | ||| |  | B:VAL  98 |
| H | B:PRO 100 | ||| |  | C:ASP  94 | ||| |  | B:ASP  99 | ||| |  | C:ASP  94 | ||| |  | B:ASP  99 | ||| |  | C:ASP  94 | ||| |  | B:ASP  99 | ||| |  | C:ASP  94 | ||| |  | B:ASP  99 | ||| |  | B:ASP  99 |
| H | B:GLU 101 | ||| | H | C:PRO  95 | ||| | H | B:PRO 100 | ||| | H | C:PRO  95 | ||| | H | B:PRO 100 | ||| |  | C:PRO  95 | ||| | H | B:PRO 100 | ||| | H | C:PRO  95 | ||| | H | B:PRO 100 | ||| | H | B:PRO 100 |
| H | B:ASN 102 | ||| | H | C:VAL  96 | ||| | H | B:GLU 101 | ||| | H | C:VAL  96 | ||| | H | B:GLU 101 | ||| | H | C:VAL  96 | ||| | H | B:GLU 101 | ||| | H | C:VAL  96 | ||| | H | B:GLU 101 | ||| | H | B:GLU 101 |
| H | B:PHE 103 | ||| | H | C:ASN  97 | ||| | H | B:ASN 102 | ||| | H | C:ASN  97 | ||| | H | B:ASN 102 | ||| | H | C:ASN  97 | ||| | H | B:ASN 102 | ||| | H | C:ASN  97 | ||| | H | B:ASN 102 | ||| | H | B:ASN 102 |
| H | B:ARG 104 | ||| | H | C:PHE  98 | ||| | H | B:PHE 103 | ||| | H | C:PHE  98 | ||| | H | B:PHE 103 | ||| | H | C:PHE  98 | ||| | H | B:PHE 103 | ||| | H | C:PHE  98 | ||| | H | B:PHE 103 | ||| | H | B:PHE 103 |
| H | B:LEU 105 | ||| | H | C:LYS  99 | ||| | H | B:ARG 104 | ||| | H | C:LYS  99 | ||| | H | B:ARG 104 | ||| | H | C:LYS  99 | ||| | H | B:ARG 104 | ||| | H | C:LYS  99 | ||| | H | B:ARG 104 | ||| | H | B:ARG 104 |
| H | B:LEU 106 | ||| | H | C:LEU 100 | ||| | H | B:LEU 105 | ||| | H | C:LEU 100 | ||| | H | B:LEU 105 | ||| | H | C:LEU 100 | ||| | H | B:LEU 105 | ||| | H | C:LEU 100 | ||| | H | B:LEU 105 | ||| | H | B:LEU 105 |
| H | B:GLY 107 | ||| | H | C:LEU 101 | ||| | H | B:LEU 106 | ||| | H | C:LEU 101 | ||| | H | B:LEU 106 | ||| | H | C:LEU 101 | ||| | H | B:LEU 106 | ||| | H | C:LEU 101 | ||| | H | B:LEU 106 | ||| | H | B:LEU 106 |
| H | B:ASN 108 | ||| | H | C:SER 102 | ||| | H | B:GLY 107 | ||| | H | C:SER 102 | ||| | H | B:GLY 107 | ||| | H | C:SER 102 | ||| | H | B:GLY 107 | ||| | H | C:SER 102 | ||| | H | B:GLY 107 | ||| | H | B:GLY 107 |
| H | B:VAL 109 | ||| | H | C:HIS 103 | ||| | H | B:ASN 108 | ||| | H | C:HIS 103 | ||| | H | B:ASN 108 | ||| | H | C:HIS 103 | ||| | H | B:ASN 108 | ||| | H | C:HIS 103 | ||| | H | B:ASN 108 | ||| | H | B:ASN 108 |
| H | B:LEU 110 | ||| | H | C:CYS 104 | ||| | H | B:VAL 109 | ||| | H | C:CYS 104 | ||| | H | B:VAL 109 | ||| | H | C:CYS 104 | ||| | H | B:VAL 109 | ||| | H | C:CYS 104 | ||| | H | B:VAL 109 | ||| | H | B:VAL 109 |
| H | B:VAL 111 | ||| | H | C:LEU 105 | ||| | H | B:LEU 110 | ||| | H | C:LEU 105 | ||| | H | B:LEU 110 | ||| | H | C:LEU 105 | ||| | H | B:LEU 110 | ||| | H | C:LEU 105 | ||| | H | B:LEU 110 | ||| | H | B:LEU 110 |
| H | B:CYS 112 | ||| | H | C:LEU 106 | ||| | H | B:VAL 111 | ||| | H | C:LEU 106 | ||| | H | B:VAL 111 | ||| | H | C:LEU 106 | ||| | H | B:VAL 111 | ||| | H | C:LEU 106 | ||| | H | B:VAL 111 | ||| | H | B:VAL 111 |
| H | B:VAL 113 | ||| | H | C:VAL 107 | ||| | H | B:CYS 112 | ||| | H | C:VAL 107 | ||| | H | B:CYS 112 | ||| | H | C:VAL 107 | ||| | H | B:CYS 112 | ||| | H | C:VAL 107 | ||| | H | B:CYS 112 | ||| | H | B:CYS 112 |
| H | B:LEU 114 | ||| | H | C:THR 108 | ||| | H | B:VAL 113 | ||| | H | C:THR 108 | ||| | H | B:VAL 113 | ||| | H | C:THR 108 | ||| | H | B:VAL 113 | ||| | H | C:THR 108 | ||| | H | B:VAL 113 | ||| | H | B:VAL 113 |
| H | B:ALA 115 | ||| | H | C:LEU 109 | ||| | H | B:LEU 114 | ||| | H | C:LEU 109 | ||| | H | B:LEU 114 | ||| | H | C:LEU 109 | ||| | H | B:LEU 114 | ||| | H | C:LEU 109 | ||| | H | B:LEU 114 | ||| | H | B:LEU 114 |
| H | B:HIS 116 | ||| | H | C:ALA 110 | ||| | H | B:ALA 115 | ||| | H | C:ALA 110 | ||| | H | B:ALA 115 | ||| | H | C:ALA 110 | ||| | H | B:ALA 115 | ||| | H | C:ALA 110 | ||| | H | B:ALA 115 | ||| | H | B:ALA 115 |
| H | B:HIS 117 | ||| | H | C:ALA 111 | ||| | H | B:HIS 116 | ||| | H | C:ALA 111 | ||| | H | B:HIS 116 | ||| | H | C:ALA 111 | ||| | H | B:HIS 116 | ||| | H | C:ALA 111 | ||| | H | B:HIS 116 | ||| | H | B:HIS 116 |
| H | B:PHE 118 | ||| | H | C:HIS 112 | ||| | H | B:HIS 117 | ||| | H | C:HIS 112 | ||| | H | B:HIS 117 | ||| | H | C:HIS 112 | ||| | H | B:HIS 117 | ||| | H | C:HIS 112 | ||| | H | B:HIS 117 | ||| | H | B:HIS 117 |
| H | B:GLY 119 | ||| | H | C:LEU 113 | ||| | H | B:PHE 118 | ||| | H | C:LEU 113 | ||| | H | B:PHE 118 | ||| | H | C:LEU 113 | ||| | H | B:PHE 118 | ||| | H | C:LEU 113 | ||| | H | B:PHE 118 | ||| | H | B:PHE 118 |
|  | B:LYS 120 | ||| |  | C:PRO 114 | ||| | H | B:GLY 119 | ||| |  | C:PRO 114 | ||| | H | B:GLY 119 | ||| |  | C:PRO 114 | ||| | H | B:GLY 119 | ||| |  | C:PRO 114 | ||| | H | B:GLY 119 | ||| | H | B:GLY 119 |
|  | B:GLU 121 | ||| |  | C:ALA 115 | ||| |  | B:LYS 120 | ||| |  | C:ALA 115 | ||| |  | B:LYS 120 | ||| |  | C:ALA 115 | ||| |  | B:LYS 120 | ||| |  | C:ALA 115 | ||| |  | B:LYS 120 | ||| |  | B:LYS 120 |
|  | B:PHE 122 | ||| |  | C:GLU 116 | ||| |  | B:GLU 121 | ||| |  | C:GLU 116 | ||| |  | B:GLU 121 | ||| |  | C:GLU 116 | ||| |  | B:GLU 121 | ||| |  | C:GLU 116 | ||| |  | B:GLU 121 | ||| |  | B:GLU 121 |
| H | B:THR 123 |  |  | |  |  | |  |  | |  |  | |  |  | |  |  | |  |  | |  |  | |  |  | |
| H | B:PRO 124 |  |  | |  |  | |  |  | |  |  | |  |  | |  |  | |  |  | |  |  | |  |  | |
| H | B:PRO 125 |  |  | |  |  | |  |  | |  |  | |  |  | |  |  | |  |  | |  |  | |  |  | |
| H | B:VAL 126 | ||| |  | C:PHE 117 | ||| |  | B:PHE 122 | ||| |  | C:PHE 117 | ||| |  | B:PHE 122 | ||| |  | C:PHE 117 | ||| |  | B:PHE 122 | ||| |  | C:PHE 117 | ||| |  | B:PHE 122 | ||| |  | B:PHE 122 |
| H | B:GLN 127 | ||| | H | C:THR 118 | ||| | H | B:THR 123 | ||| | H | C:THR 118 | ||| | H | B:THR 123 | ||| | H | C:THR 118 | ||| | H | B:THR 123 | ||| | H | C:THR 118 | ||| | H | B:THR 123 | ||| | H | B:THR 123 |
| H | B:ALA 128 | ||| | H | C:PRO 119 | ||| | H | B:PRO 124 | ||| | H | C:PRO 119 | ||| | H | B:PRO 124 | ||| | H | C:PRO 119 | ||| | H | B:PRO 124 | ||| | H | C:PRO 119 | ||| | H | B:PRO 124 | ||| | H | B:PRO 124 |
| H | B:ALA 129 | ||| | H | C:ALA 120 | ||| | H | B:PRO 125 | ||| | H | C:ALA 120 | ||| | H | B:PRO 125 | ||| | H | C:ALA 120 | ||| | H | B:PRO 125 | ||| | H | C:ALA 120 | ||| | H | B:PRO 125 | ||| | H | B:PRO 125 |
| H | B:TYR 130 | ||| | H | C:VAL 121 | ||| | H | B:VAL 126 | ||| | H | C:VAL 121 | ||| | H | B:VAL 126 | ||| | H | C:VAL 121 | ||| | H | B:VAL 126 | ||| | H | C:VAL 121 | ||| | H | B:VAL 126 | ||| | H | B:VAL 126 |
| H | B:GLN 131 | ||| | H | C:HIS 122 | ||| | H | B:GLN 127 | ||| | H | C:HIS 122 | ||| | H | B:GLN 127 | ||| | H | C:HIS 122 | ||| | H | B:GLN 127 | ||| | H | C:HIS 122 | ||| | H | B:GLN 127 | ||| | H | B:GLN 127 |
| H | B:LYS 132 | ||| | H | C:ALA 123 | ||| | H | B:ALA 128 | ||| | H | C:ALA 123 | ||| | H | B:ALA 128 | ||| | H | C:ALA 123 | ||| | H | B:ALA 128 | ||| | H | C:ALA 123 | ||| | H | B:ALA 128 | ||| | H | B:ALA 128 |
| H | B:VAL 133 | ||| | H | C:SER 124 | ||| | H | B:ALA 129 | ||| | H | C:SER 124 | ||| | H | B:ALA 129 | ||| | H | C:SER 124 | ||| | H | B:ALA 129 | ||| | H | C:SER 124 | ||| | H | B:ALA 129 | ||| | H | B:ALA 129 |
| H | B:VAL 134 | ||| | H | C:LEU 125 | ||| | H | B:TYR 130 | ||| | H | C:LEU 125 | ||| | H | B:TYR 130 | ||| | H | C:LEU 125 | ||| | H | B:TYR 130 | ||| | H | C:LEU 125 | ||| | H | B:TYR 130 | ||| | H | B:TYR 130 |
| H | B:ALA 135 | ||| | H | C:ASP 126 | ||| | H | B:GLN 131 | ||| | H | C:ASP 126 | ||| | H | B:GLN 131 | ||| | H | C:ASP 126 | ||| | H | B:GLN 131 | ||| | H | C:ASP 126 | ||| | H | B:GLN 131 | ||| | H | B:GLN 131 |
| H | B:GLY 136 | ||| | H | C:LYS 127 | ||| | H | B:LYS 132 | ||| | H | C:LYS 127 | ||| | H | B:LYS 132 | ||| | H | C:LYS 127 | ||| | H | B:LYS 132 | ||| | H | C:LYS 127 | ||| | H | B:LYS 132 | ||| | H | B:LYS 132 |
| H | B:VAL 137 | ||| | H | C:PHE 128 | ||| | H | B:VAL 133 | ||| | H | C:PHE 128 | ||| | H | B:VAL 133 | ||| | H | C:PHE 128 | ||| | H | B:VAL 133 | ||| | H | C:PHE 128 | ||| | H | B:VAL 133 | ||| | H | B:VAL 133 |
| H | B:ALA 138 | ||| | H | C:LEU 129 | ||| | H | B:VAL 134 | ||| | H | C:LEU 129 | ||| | H | B:VAL 134 | ||| | H | C:LEU 129 | ||| | H | B:VAL 134 | ||| | H | C:LEU 129 | ||| | H | B:VAL 134 | ||| | H | B:VAL 134 |
| H | B:ASN 139 | ||| | H | C:ALA 130 | ||| | H | B:ALA 135 | ||| | H | C:ALA 130 | ||| | H | B:ALA 135 | ||| | H | C:ALA 130 | ||| | H | B:ALA 135 | ||| | H | C:ALA 130 | ||| | H | B:ALA 135 | ||| | H | B:ALA 135 |
| H | B:ALA 140 | ||| | H | C:SER 131 | ||| | H | B:GLY 136 | ||| | H | C:SER 131 | ||| | H | B:GLY 136 | ||| | H | C:SER 131 | ||| | H | B:GLY 136 | ||| | H | C:SER 131 | ||| | H | B:GLY 136 | ||| | H | B:GLY 136 |
| H | B:LEU 141 | ||| | H | C:VAL 132 | ||| | H | B:VAL 137 | ||| | H | C:VAL 132 | ||| | H | B:VAL 137 | ||| | H | C:VAL 132 | ||| | H | B:VAL 137 | ||| | H | C:VAL 132 | ||| | H | B:VAL 137 | ||| | H | B:VAL 137 |
| H | B:ALA 142 | ||| | H | C:SER 133 | ||| | H | B:ALA 138 | ||| | H | C:SER 133 | ||| | H | B:ALA 138 | ||| | H | C:SER 133 | ||| | H | B:ALA 138 | ||| | H | C:SER 133 | ||| | H | B:ALA 138 | ||| | H | B:ALA 138 |
|  | B:HIS 143 | ||| | H | C:THR 134 | ||| | H | B:ASN 139 | ||| | H | C:THR 134 | ||| | H | B:ASN 139 | ||| | H | C:THR 134 | ||| | H | B:ASN 139 | ||| | H | C:THR 134 | ||| | H | B:ASN 139 | ||| | H | B:ASN 139 |
|  | B:LYS 144 | ||| | H | C:VAL 135 | ||| | H | B:ALA 140 | ||| | H | C:VAL 135 | ||| | H | B:ALA 140 | ||| | H | C:VAL 135 | ||| | H | B:ALA 140 | ||| | H | C:VAL 135 | ||| | H | B:ALA 140 | ||| | H | B:ALA 140 |
|  | C:VAL   1 |  |  | |  |  | |  |  | |  |  | |  |  | |  |  | |  |  | |  |  | |  |  | |
|  | C:LEU   2 | ||| | H | C:LEU 136 | ||| | H | B:LEU 141 | ||| | H | C:LEU 136 | ||| | H | B:LEU 141 | ||| | H | C:LEU 136 | ||| | H | B:LEU 141 | ||| | H | C:LEU 136 | ||| | H | B:LEU 141 | ||| | H | B:LEU 141 |
|  | |  |  | |  | H | B:ALA 142 |  |  | |  | H | B:ALA 142 |  |  | |  | H | B:ALA 142 |  |  | |  | H | B:ALA 142 |  | H | B:ALA 142 |
|  | |  |  | |  | H | B:HIS 143 |  |  | |  | H | B:HIS 143 |  |  | |  | H | B:HIS 143 |  |  | |  | H | B:HIS 143 |  | H | B:HIS 143 |
|  | |  |  | |  |  | B:LYS 144 |  |  | |  |  | B:LYS 144 |  |  | |  |  | B:LYS 144 |  |  | |  |  | B:LYS 144 |  |  | B:LYS 144 |
|  | |  |  | |  |  | B:TYR 145 |  |  | |  |  | B:TYR 145 |  |  | |  |  | B:TYR 145 |  |  | |  |  | B:TYR 145 |  |  | B:TYR 145 |
|  | |  |  | |  |  | B:HIS 146 |  |  | |  |  | B:HIS 146 |  |  | |  |  | B:HIS 146 |  |  | |  |  | B:HIS 146 |  |  | B:HIS 146 |
| H | C:SER   3 | ||| | H | C:THR 137 | ||| |  | C:VAL   1 | ||| | H | C:THR 137 | ||| |  | C:VAL   1 | ||| | H | C:THR 137 | ||| |  | C:VAL   1 | ||| | H | C:THR 137 | ||| |  | C:VAL   1 | ||| |  | C:VAL   1 |
| H | C:PRO   4 |  |  | |  |  | |  |  | |  |  | |  |  | |  |  | |  |  | |  |  | |  |  | |
| H | C:ALA   5 |  |  | |  |  | |  |  | |  |  | |  |  | |  |  | |  |  | |  |  | |  |  | |
| H | C:ASP   6 | ||| | H | C:SER 138 | ||| |  | C:LEU   2 | ||| | H | C:SER 138 | ||| |  | C:LEU   2 | ||| | H | C:SER 138 | ||| |  | C:LEU   2 | ||| | H | C:SER 138 | ||| |  | C:LEU   2 | ||| |  | C:LEU   2 |
| H | C:LYS   7 | ||| |  | C:LYS 139 | ||| | H | C:SER   3 | ||| |  | C:LYS 139 | ||| | H | C:SER   3 | ||| |  | C:LYS 139 | ||| | H | C:SER   3 | ||| |  | C:LYS 139 | ||| | H | C:SER   3 | ||| | H | C:SER   3 |
| H | C:THR   8 | ||| |  | C:TYR 140 | ||| | H | C:PRO   4 | ||| |  | C:TYR 140 | ||| | H | C:PRO   4 | ||| |  | C:TYR 140 | ||| | H | C:PRO   4 | ||| |  | C:TYR 140 | ||| | H | C:PRO   4 | ||| | H | C:PRO   4 |
| H | C:ASN   9 | ||| |  | C:ARG 141 | ||| | H | C:ALA   5 | ||| |  | C:ARG 141 | ||| | H | C:ALA   5 | ||| |  | C:ARG 141 | ||| | H | C:ALA   5 | ||| |  | C:ARG 141 | ||| | H | C:ALA   5 | ||| | H | C:ALA   5 |
| H | C:VAL  10 | ||| |  | B:MET   1 | ||| | H | C:ASP   6 | ||| |  | B:MET   1 | ||| | H | C:ASP   6 | ||| |  | B:MET   1 | ||| | H | C:ASP   6 | ||| |  | B:MET   1 | ||| | H | C:ASP   6 | ||| | H | C:ASP   6 |
| H | C:LYS  11 | ||| |  | B:HIS   2 | ||| | H | C:LYS   7 | ||| |  | B:HIS   2 | ||| | H | C:LYS   7 | ||| |  | B:HIS   2 | ||| | H | C:LYS   7 | ||| |  | B:HIS   2 | ||| | H | C:LYS   7 | ||| | H | C:LYS   7 |
| H | C:ALA  12 | ||| |  | B:LEU   3 | ||| | H | C:THR   8 | ||| |  | B:LEU   3 | ||| | H | C:THR   8 | ||| |  | B:LEU   3 | ||| | H | C:THR   8 | ||| |  | B:LEU   3 | ||| | H | C:THR   8 | ||| | H | C:THR   8 |
| H | C:ALA  13 | ||| | H | B:THR   4 | ||| | H | C:ASN   9 | ||| | H | B:THR   4 | ||| | H | C:ASN   9 | ||| | H | B:THR   4 | ||| | H | C:ASN   9 | ||| | H | B:THR   4 | ||| | H | C:ASN   9 | ||| | H | C:ASN   9 |
| H | C:TRP  14 | ||| | H | B:PRO   5 | ||| | H | C:VAL  10 | ||| | H | B:PRO   5 | ||| | H | C:VAL  10 | ||| | H | B:PRO   5 | ||| | H | C:VAL  10 | ||| | H | B:PRO   5 | ||| | H | C:VAL  10 | ||| | H | C:VAL  10 |
| H | C:GLY  15 | ||| | H | B:GLU   6 | ||| | H | C:LYS  11 | ||| | H | B:GLU   6 | ||| | H | C:LYS  11 | ||| | H | B:GLU   6 | ||| | H | C:LYS  11 | ||| | H | B:GLU   6 | ||| | H | C:LYS  11 | ||| | H | C:LYS  11 |
| H | C:LYS  16 | ||| | H | B:GLU   7 | ||| | H | C:ALA  12 | ||| | H | B:GLU   7 | ||| | H | C:ALA  12 | ||| | H | B:GLU   7 | ||| | H | C:ALA  12 | ||| | H | B:GLU   7 | ||| | H | C:ALA  12 | ||| | H | C:ALA  12 |
| H | C:VAL  17 | ||| | H | B:LYS   8 | ||| | H | C:ALA  13 | ||| | H | B:LYS   8 | ||| | H | C:ALA  13 | ||| | H | B:LYS   8 | ||| | H | C:ALA  13 | ||| | H | B:LYS   8 | ||| | H | C:ALA  13 | ||| | H | C:ALA  13 |
| H | C:GLY  18 | ||| | H | B:SER   9 | ||| | H | C:TRP  14 | ||| | H | B:SER   9 | ||| | H | C:TRP  14 | ||| | H | B:SER   9 | ||| | H | C:TRP  14 | ||| | H | B:SER   9 | ||| | H | C:TRP  14 | ||| | H | C:TRP  14 |
|  | |  |  | |  | H | C:GLY  15 |  |  | |  | H | C:GLY  15 |  |  | |  | H | C:GLY  15 |  |  | |  | H | C:GLY  15 |  | H | C:GLY  15 |
|  | |  |  | |  | H | C:LYS  16 |  |  | |  | H | C:LYS  16 |  |  | |  | H | C:LYS  16 |  |  | |  | H | C:LYS  16 |  | H | C:LYS  16 |
|  | |  |  | |  | H | C:VAL  17 |  |  | |  | H | C:VAL  17 |  |  | |  | H | C:VAL  17 |  |  | |  | H | C:VAL  17 |  | H | C:VAL  17 |
|  | |  |  | |  | H | C:GLY  18 |  |  | |  | H | C:GLY  18 |  |  | |  | H | C:GLY  18 |  |  | |  | H | C:GLY  18 |  | H | C:GLY  18 |
|  | C:ALA  19 | ||| | H | B:ALA  10 | ||| |  | C:ALA  19 | ||| | H | B:ALA  10 | ||| |  | C:ALA  19 | ||| | H | B:ALA  10 | ||| |  | C:ALA  19 | ||| | H | B:ALA  10 | ||| |  | C:ALA  19 | ||| |  | C:ALA  19 |
| H | C:HIS  20 | ||| | H | B:VAL  11 | ||| | H | C:HIS  20 | ||| | H | B:VAL  11 | ||| | H | C:HIS  20 | ||| | H | B:VAL  11 | ||| | H | C:HIS  20 | ||| | H | B:VAL  11 | ||| | H | C:HIS  20 | ||| | H | C:HIS  20 |
| H | C:ALA  21 | ||| | H | B:THR  12 | ||| | H | C:ALA  21 | ||| | H | B:THR  12 | ||| | H | C:ALA  21 | ||| | H | B:THR  12 | ||| | H | C:ALA  21 | ||| | H | B:THR  12 | ||| | H | C:ALA  21 | ||| | H | C:ALA  21 |
| H | C:GLY  22 | ||| | H | B:ALA  13 | ||| | H | C:GLY  22 | ||| | H | B:ALA  13 | ||| | H | C:GLY  22 | ||| | H | B:ALA  13 | ||| | H | C:GLY  22 | ||| | H | B:ALA  13 | ||| | H | C:GLY  22 | ||| | H | C:GLY  22 |
| H | C:GLU  23 | ||| | H | B:LEU  14 | ||| | H | C:GLU  23 | ||| | H | B:LEU  14 | ||| | H | C:GLU  23 | ||| | H | B:LEU  14 | ||| | H | C:GLU  23 | ||| | H | B:LEU  14 | ||| | H | C:GLU  23 | ||| | H | C:GLU  23 |
| H | C:TYR  24 | ||| | H | B:TRP  15 | ||| | H | C:TYR  24 | ||| | H | B:TRP  15 | ||| | H | C:TYR  24 | ||| | H | B:TRP  15 | ||| | H | C:TYR  24 | ||| | H | B:TRP  15 | ||| | H | C:TYR  24 | ||| | H | C:TYR  24 |
| H | C:GLY  25 | ||| | H | B:GLY  16 | ||| | H | C:GLY  25 | ||| | H | B:GLY  16 | ||| | H | C:GLY  25 | ||| | H | B:GLY  16 | ||| | H | C:GLY  25 | ||| | H | B:GLY  16 | ||| | H | C:GLY  25 | ||| | H | C:GLY  25 |
| H | C:ALA  26 | ||| |  | B:LYS  17 | ||| | H | C:ALA  26 | ||| |  | B:LYS  17 | ||| | H | C:ALA  26 | ||| |  | B:LYS  17 | ||| | H | C:ALA  26 | ||| |  | B:LYS  17 | ||| | H | C:ALA  26 | ||| | H | C:ALA  26 |
| H | C:GLU  27 | ||| |  | B:VAL  18 | ||| | H | C:GLU  27 | ||| |  | B:VAL  18 | ||| | H | C:GLU  27 | ||| |  | B:VAL  18 | ||| | H | C:GLU  27 | ||| |  | B:VAL  18 | ||| | H | C:GLU  27 | ||| | H | C:GLU  27 |
| H | C:ALA  28 | ||| | H | B:ASN  19 | ||| | H | C:ALA  28 | ||| | H | B:ASN  19 | ||| | H | C:ALA  28 | ||| | H | B:ASN  19 | ||| | H | C:ALA  28 | ||| | H | B:ASN  19 | ||| | H | C:ALA  28 | ||| | H | C:ALA  28 |
|  | |  | H | B:VAL  20 |  |  | |  | H | B:VAL  20 |  |  | |  | H | B:VAL  20 |  |  | |  | H | B:VAL  20 |  |  | |  |  | |
| H | C:LEU  29 | ||| | H | B:ASP  21 | ||| | H | C:LEU  29 | ||| | H | B:ASP  21 | ||| | H | C:LEU  29 | ||| | H | B:ASP  21 | ||| | H | C:LEU  29 | ||| | H | B:ASP  21 | ||| | H | C:LEU  29 | ||| | H | C:LEU  29 |
| H | C:GLU  30 | ||| | H | B:GLU  22 | ||| | H | C:GLU  30 | ||| | H | B:GLU  22 | ||| | H | C:GLU  30 | ||| | H | B:GLU  22 | ||| | H | C:GLU  30 | ||| | H | B:GLU  22 | ||| | H | C:GLU  30 | ||| | H | C:GLU  30 |
| H | C:ARG  31 | ||| | H | B:VAL  23 | ||| | H | C:ARG  31 | ||| | H | B:VAL  23 | ||| | H | C:ARG  31 | ||| | H | B:VAL  23 | ||| | H | C:ARG  31 | ||| | H | B:VAL  23 | ||| | H | C:ARG  31 | ||| | H | C:ARG  31 |
| H | C:MET  32 | ||| | H | B:GLY  24 | ||| | H | C:MET  32 | ||| | H | B:GLY  24 | ||| | H | C:MET  32 | ||| | H | B:GLY  24 | ||| | H | C:MET  32 | ||| | H | B:GLY  24 | ||| | H | C:MET  32 | ||| | H | C:MET  32 |
| H | C:PHE  33 | ||| | H | B:GLY  25 | ||| | H | C:PHE  33 | ||| | H | B:GLY  25 | ||| | H | C:PHE  33 | ||| | H | B:GLY  25 | ||| | H | C:PHE  33 | ||| | H | B:GLY  25 | ||| | H | C:PHE  33 | ||| | H | C:PHE  33 |
| H | C:LEU  34 | ||| | H | B:GLU  26 | ||| | H | C:LEU  34 | ||| | H | B:GLU  26 | ||| | H | C:LEU  34 | ||| | H | B:GLU  26 | ||| | H | C:LEU  34 | ||| | H | B:GLU  26 | ||| | H | C:LEU  34 | ||| | H | C:LEU  34 |
| H | C:SER  35 | ||| | H | B:ALA  27 | ||| | H | C:SER  35 | ||| | H | B:ALA  27 | ||| | H | C:SER  35 | ||| | H | B:ALA  27 | ||| | H | C:SER  35 | ||| | H | B:ALA  27 | ||| | H | C:SER  35 | ||| | H | C:SER  35 |
| H | C:PHE  36 | ||| | H | B:LEU  28 | ||| | H | C:PHE  36 | ||| | H | B:LEU  28 | ||| | H | C:PHE  36 | ||| | H | B:LEU  28 | ||| | H | C:PHE  36 | ||| | H | B:LEU  28 | ||| | H | C:PHE  36 | ||| | H | C:PHE  36 |
| H | C:PRO  37 | ||| | H | B:GLY  29 | ||| | H | C:PRO  37 | ||| | H | B:GLY  29 | ||| | H | C:PRO  37 | ||| | H | B:GLY  29 | ||| | H | C:PRO  37 | ||| | H | B:GLY  29 | ||| | H | C:PRO  37 | ||| | H | C:PRO  37 |
| H | C:THR  38 | ||| | H | B:ARG  30 | ||| | H | C:THR  38 | ||| | H | B:ARG  30 | ||| | H | C:THR  38 | ||| | H | B:ARG  30 | ||| | H | C:THR  38 | ||| | H | B:ARG  30 | ||| | H | C:THR  38 | ||| | H | C:THR  38 |
| H | C:THR  39 | ||| | H | B:LEU  31 | ||| | H | C:THR  39 | ||| | H | B:LEU  31 | ||| | H | C:THR  39 | ||| | H | B:LEU  31 | ||| | H | C:THR  39 | ||| | H | B:LEU  31 | ||| | H | C:THR  39 | ||| | H | C:THR  39 |
| H | C:LYS  40 | ||| | H | B:LEU  32 | ||| | H | C:LYS  40 | ||| | H | B:LEU  32 | ||| | H | C:LYS  40 | ||| | H | B:LEU  32 | ||| | H | C:LYS  40 | ||| | H | B:LEU  32 | ||| | H | C:LYS  40 | ||| | H | C:LYS  40 |
| H | C:THR  41 | ||| | H | B:VAL  33 | ||| | H | C:THR  41 | ||| | H | B:VAL  33 | ||| | H | C:THR  41 | ||| | H | B:VAL  33 | ||| | H | C:THR  41 | ||| | H | B:VAL  33 | ||| | H | C:THR  41 | ||| | H | C:THR  41 |
| H | C:TYR  42 | ||| | H | B:VAL  34 | ||| | H | C:TYR  42 | ||| | H | B:VAL  34 | ||| | H | C:TYR  42 | ||| | H | B:VAL  34 | ||| | H | C:TYR  42 | ||| | H | B:VAL  34 | ||| | H | C:TYR  42 | ||| | H | C:TYR  42 |
| H | C:PHE  43 | ||| | H | B:TYR  35 | ||| | H | C:PHE  43 | ||| | H | B:TYR  35 | ||| | H | C:PHE  43 | ||| | H | B:TYR  35 | ||| | H | C:PHE  43 | ||| | H | B:TYR  35 | ||| | H | C:PHE  43 | ||| | H | C:PHE  43 |
|  | C:PRO  44 | ||| | H | B:PRO  36 | ||| |  | C:PRO  44 | ||| | H | B:PRO  36 | ||| |  | C:PRO  44 | ||| | H | B:PRO  36 | ||| |  | C:PRO  44 | ||| | H | B:PRO  36 | ||| |  | C:PRO  44 | ||| |  | C:PRO  44 |
|  | C:HIS  45 | ||| | H | B:ALA  37 | ||| |  | C:HIS  45 | ||| | H | B:GLU  37 | ||| |  | C:HIS  45 | ||| | H | B:TYR  37 | ||| |  | C:HIS  45 | ||| | H | B:GLY  37 | ||| |  | C:HIS  45 | ||| |  | C:HIS  45 |
|  | C:PHE  46 | ||| | H | B:THR  38 | ||| |  | C:PHE  46 | ||| | H | B:THR  38 | ||| |  | C:PHE  46 | ||| | H | B:THR  38 | ||| |  | C:PHE  46 | ||| | H | B:THR  38 | ||| |  | C:PHE  46 | ||| |  | C:PHE  46 |
|  | C:ASP  47 | ||| | H | B:GLN  39 | ||| |  | C:ASP  47 | ||| | H | B:GLN  39 | ||| |  | C:ASP  47 | ||| | H | B:GLN  39 | ||| |  | C:ASP  47 | ||| | H | B:GLN  39 | ||| |  | C:ASP  47 | ||| |  | C:ASP  47 |
|  | C:LEU  48 | ||| | H | B:ARG  40 | ||| |  | C:LEU  48 | ||| | H | B:ARG  40 | ||| |  | C:LEU  48 | ||| | H | B:ARG  40 | ||| |  | C:LEU  48 | ||| | H | B:ARG  40 | ||| |  | C:LEU  48 | ||| |  | C:LEU  48 |
|  | C:SER  49 | ||| | H | B:PHE  41 | ||| |  | C:SER  49 | ||| | H | B:PHE  41 | ||| |  | C:SER  49 | ||| | H | B:PHE  41 | ||| |  | C:SER  49 | ||| | H | B:PHE  41 | ||| |  | C:SER  49 | ||| |  | C:SER  49 |
|  | C:HIS  50 | ||| | H | B:PHE  42 | ||| |  | C:HIS  50 | ||| | H | B:PHE  42 | ||| |  | C:HIS  50 | ||| | H | B:PHE  42 | ||| |  | C:HIS  50 | ||| | H | B:PHE  42 | ||| |  | C:HIS  50 | ||| |  | C:HIS  50 |
|  | C:GLY  51 | ||| | H | B:GLU  43 | ||| |  | C:GLY  51 | ||| | H | B:GLU  43 | ||| |  | C:GLY  51 | ||| | H | B:GLU  43 | ||| |  | C:GLY  51 | ||| | H | B:GLU  43 | ||| |  | C:GLY  51 | ||| |  | C:GLY  51 |
| H | C:SER  52 | ||| | H | B:SER  44 | ||| | H | C:SER  52 | ||| | H | B:SER  44 | ||| | H | C:SER  52 | ||| | H | B:SER  44 | ||| | H | C:SER  52 | ||| | H | B:SER  44 | ||| | H | C:SER  52 | ||| | H | C:SER  52 |
| H | C:ALA  53 | ||| | H | B:PHE  45 | ||| | H | C:ALA  53 | ||| | H | B:PHE  45 | ||| | H | C:ALA  53 | ||| | H | B:PHE  45 | ||| | H | C:ALA  53 | ||| | H | B:PHE  45 | ||| | H | C:ALA  53 | ||| | H | C:ALA  53 |
| H | C:GLN  54 | ||| | H | B:GLY  46 | ||| | H | C:GLN  54 | ||| | H | B:GLY  46 | ||| | H | C:GLN  54 | ||| | H | B:GLY  46 | ||| | H | C:GLN  54 | ||| | H | B:GLY  46 | ||| | H | C:GLN  54 | ||| | H | C:GLN  54 |
| H | C:VAL  55 | ||| |  | B:ASP  47 | ||| | H | C:VAL  55 | ||| |  | B:ASP  47 | ||| | H | C:VAL  55 | ||| |  | B:ASP  47 | ||| | H | C:VAL  55 | ||| |  | B:ASP  47 | ||| | H | C:VAL  55 | ||| | H | C:VAL  55 |
| H | C:LYS  56 | ||| |  | B:LEU  48 | ||| | H | C:LYS  56 | ||| |  | B:LEU  48 | ||| | H | C:LYS  56 | ||| |  | B:LEU  48 | ||| | H | C:LYS  56 | ||| |  | B:LEU  48 | ||| | H | C:LYS  56 | ||| | H | C:LYS  56 |
|  | |  |  | B:SER  49 |  |  | |  |  | B:SER  49 |  |  | |  |  | B:SER  49 |  |  | |  |  | B:SER  49 |  |  | |  |  | |
| H | C:GLY  57 | ||| | H | B:THR  50 | ||| | H | C:GLY  57 | ||| | H | B:THR  50 | ||| | H | C:GLY  57 | ||| | H | B:THR  50 | ||| | H | C:GLY  57 | ||| | H | B:THR  50 | ||| | H | C:GLY  57 | ||| | H | C:GLY  57 |
| H | C:HIS  58 | ||| | H | B:PRO  51 | ||| | H | C:HIS  58 | ||| | H | B:PRO  51 | ||| | H | C:HIS  58 | ||| | H | B:PRO  51 | ||| | H | C:HIS  58 | ||| | H | B:PRO  51 | ||| | H | C:HIS  58 | ||| | H | C:HIS  58 |
| H | C:GLY  59 | ||| | H | B:ASP  52 | ||| | H | C:GLY  59 | ||| | H | B:ASP  52 | ||| | H | C:GLY  59 | ||| | H | B:ASP  52 | ||| | H | C:GLY  59 | ||| | H | B:ASP  52 | ||| | H | C:GLY  59 | ||| | H | C:GLY  59 |
| H | C:LYS  60 | ||| | H | B:ALA  53 | ||| | H | C:LYS  60 | ||| | H | B:ALA  53 | ||| | H | C:LYS  60 | ||| | H | B:ALA  53 | ||| | H | C:LYS  60 | ||| | H | B:ALA  53 | ||| | H | C:LYS  60 | ||| | H | C:LYS  60 |
| H | C:LYS  61 | ||| | H | B:VAL  54 | ||| | H | C:LYS  61 | ||| | H | B:VAL  54 | ||| | H | C:LYS  61 | ||| | H | B:VAL  54 | ||| | H | C:LYS  61 | ||| | H | B:VAL  54 | ||| | H | C:LYS  61 | ||| | H | C:LYS  61 |
| H | C:VAL  62 | ||| | H | B:MET  55 | ||| | H | C:VAL  62 | ||| | H | B:MET  55 | ||| | H | C:VAL  62 | ||| | H | B:MET  55 | ||| | H | C:VAL  62 | ||| | H | B:MET  55 | ||| | H | C:VAL  62 | ||| | H | C:VAL  62 |
| H | C:ALA  63 | ||| | H | B:GLY  56 | ||| | H | C:ALA  63 | ||| | H | B:GLY  56 | ||| | H | C:ALA  63 | ||| | H | B:GLY  56 | ||| | H | C:ALA  63 | ||| | H | B:GLY  56 | ||| | H | C:ALA  63 | ||| | H | C:ALA  63 |
|  | |  | H | B:ASN  57 |  |  | |  | H | B:ASN  57 |  |  | |  | H | B:ASN  57 |  |  | |  | H | B:ASN  57 |  |  | |  |  | |
|  | |  | H | B:PRO  58 |  |  | |  | H | B:PRO  58 |  |  | |  | H | B:PRO  58 |  |  | |  | H | B:PRO  58 |  |  | |  |  | |
|  | |  | H | B:LYS  59 |  |  | |  | H | B:LYS  59 |  |  | |  | H | B:LYS  59 |  |  | |  | H | B:LYS  59 |  |  | |  |  | |
| H | C:ASP  64 | ||| | H | B:VAL  60 | ||| | H | C:ASP  64 | ||| | H | B:VAL  60 | ||| | H | C:ASP  64 | ||| | H | B:VAL  60 | ||| | H | C:ASP  64 | ||| | H | B:VAL  60 | ||| | H | C:ASP  64 | ||| | H | C:ASP  64 |
| H | C:ALA  65 | ||| | H | B:LYS  61 | ||| | H | C:ALA  65 | ||| | H | B:LYS  61 | ||| | H | C:ALA  65 | ||| | H | B:LYS  61 | ||| | H | C:ALA  65 | ||| | H | B:LYS  61 | ||| | H | C:ALA  65 | ||| | H | C:ALA  65 |
| H | C:LEU  66 | ||| | H | B:ALA  62 | ||| | H | C:LEU  66 | ||| | H | B:ALA  62 | ||| | H | C:LEU  66 | ||| | H | B:ALA  62 | ||| | H | C:LEU  66 | ||| | H | B:ALA  62 | ||| | H | C:LEU  66 | ||| | H | C:LEU  66 |
| H | C:THR  67 | ||| | H | B:HIS  63 | ||| | H | C:THR  67 | ||| | H | B:HIS  63 | ||| | H | C:THR  67 | ||| | H | B:HIS  63 | ||| | H | C:THR  67 | ||| | H | B:HIS  63 | ||| | H | C:THR  67 | ||| | H | C:THR  67 |
| H | C:ASN  68 | ||| | H | B:GLY  64 | ||| | H | C:ASN  68 | ||| | H | B:GLY  64 | ||| | H | C:ASN  68 | ||| | H | B:GLY  64 | ||| | H | C:ASN  68 | ||| | H | B:GLY  64 | ||| | H | C:ASN  68 | ||| | H | C:ASN  68 |
| H | C:ALA  69 | ||| | H | B:LYS  65 | ||| | H | C:ALA  69 | ||| | H | B:LYS  65 | ||| | H | C:ALA  69 | ||| | H | B:LYS  65 | ||| | H | C:ALA  69 | ||| | H | B:LYS  65 | ||| | H | C:ALA  69 | ||| | H | C:ALA  69 |
| H | C:VAL  70 | ||| | H | B:LYS  66 | ||| | H | C:VAL  70 | ||| | H | B:LYS  66 | ||| | H | C:VAL  70 | ||| | H | B:LYS  66 | ||| | H | C:VAL  70 | ||| | H | B:LYS  66 | ||| | H | C:VAL  70 | ||| | H | C:VAL  70 |
| H | C:ALA  71 | ||| | H | B:VAL  67 | ||| | H | C:ALA  71 | ||| | H | B:VAL  67 | ||| | H | C:ALA  71 | ||| | H | B:VAL  67 | ||| | H | C:ALA  71 | ||| | H | B:VAL  67 | ||| | H | C:ALA  71 | ||| | H | C:ALA  71 |
|  | C:HIS  72 | ||| | H | B:LEU  68 | ||| | H | C:HIS  72 | ||| | H | B:LEU  68 | ||| | H | C:HIS  72 | ||| | H | B:LEU  68 | ||| | H | C:HIS  72 | ||| | H | B:LEU  68 | ||| | H | C:HIS  72 | ||| | H | C:HIS  72 |
|  | C:VAL  73 | ||| | H | B:GLY  69 | ||| |  | C:VAL  73 | ||| | H | B:GLY  69 | ||| |  | C:VAL  73 | ||| | H | B:GLY  69 | ||| |  | C:VAL  73 | ||| | H | B:GLY  69 | ||| |  | C:VAL  73 | ||| |  | C:VAL  73 |
|  | C:ASP  74 | ||| | H | B:ALA  70 | ||| |  | C:ASP  74 | ||| | H | B:ALA  70 | ||| |  | C:ASP  74 | ||| | H | B:ALA  70 | ||| |  | C:ASP  74 | ||| | H | B:ALA  70 | ||| |  | C:ASP  74 | ||| |  | C:ASP  74 |
| H | C:ASP  75 | ||| | H | B:PHE  71 | ||| | H | C:ASP  75 | ||| | H | B:PHE  71 | ||| | H | C:ASP  75 | ||| | H | B:PHE  71 | ||| | H | C:ASP  75 | ||| | H | B:PHE  71 | ||| | H | C:ASP  75 | ||| | H | C:ASP  75 |
| H | C:MET  76 | ||| | H | B:SER  72 | ||| | H | C:MET  76 | ||| | H | B:SER  72 | ||| | H | C:MET  76 | ||| | H | B:SER  72 | ||| | H | C:MET  76 | ||| | H | B:SER  72 | ||| | H | C:MET  76 | ||| | H | C:MET  76 |
| H | C:PRO  77 | ||| | H | B:ASP  73 | ||| | H | C:PRO  77 | ||| | H | B:ASP  73 | ||| | H | C:PRO  77 | ||| | H | B:ASP  73 | ||| | H | C:PRO  77 | ||| | H | B:ASP  73 | ||| | H | C:PRO  77 | ||| | H | C:PRO  77 |
| H | C:ASN  78 | ||| | H | B:GLY  74 | ||| | H | C:ASN  78 | ||| | H | B:GLY  74 | ||| | H | C:ASN  78 | ||| | H | B:GLY  74 | ||| | H | C:ASN  78 | ||| | H | B:GLY  74 | ||| | H | C:ASN  78 | ||| | H | C:ASN  78 |
| H | C:ALA  79 | ||| | H | B:LEU  75 | ||| | H | C:ALA  79 | ||| | H | B:LEU  75 | ||| | H | C:ALA  79 | ||| | H | B:LEU  75 | ||| | H | C:ALA  79 | ||| | H | B:LEU  75 | ||| | H | C:ALA  79 | ||| | H | C:ALA  79 |
| H | C:LEU  80 | ||| | H | B:ALA  76 | ||| | H | C:LEU  80 | ||| | H | B:ALA  76 | ||| | H | C:LEU  80 | ||| |  | B:ALA  76 | ||| | H | C:LEU  80 | ||| | H | B:ALA  76 | ||| | H | C:LEU  80 | ||| | H | C:LEU  80 |
| H | C:SER  81 | ||| |  | B:HIS  77 | ||| | H | C:SER  81 | ||| |  | B:HIS  77 | ||| | H | C:SER  81 | ||| |  | B:HIS  77 | ||| | H | C:SER  81 | ||| |  | B:HIS  77 | ||| | H | C:SER  81 | ||| | H | C:SER  81 |
| H | C:ALA  82 | ||| |  | B:LEU  78 | ||| | H | C:ALA  82 | ||| |  | B:LEU  78 | ||| | H | C:ALA  82 | ||| |  | B:LEU  78 | ||| | H | C:ALA  82 | ||| |  | B:LEU  78 | ||| | H | C:ALA  82 | ||| | H | C:ALA  82 |
| H | C:LEU  83 | ||| |  | B:ASP  79 | ||| | H | C:LEU  83 | ||| |  | B:ASP  79 | ||| | H | C:LEU  83 | ||| |  | B:ASP  79 | ||| | H | C:LEU  83 | ||| |  | B:ASP  79 | ||| | H | C:LEU  83 | ||| | H | C:LEU  83 |
| H | C:SER  84 | ||| | H | B:ASN  80 | ||| | H | C:SER  84 | ||| | H | B:ASN  80 | ||| | H | C:SER  84 | ||| | H | B:ASN  80 | ||| | H | C:SER  84 | ||| | H | B:ASN  80 | ||| | H | C:SER  84 | ||| | H | C:SER  84 |
|  | |  | H | B:LEU  81 |  |  | |  | H | B:LEU  81 |  |  | |  | H | B:LEU  81 |  |  | |  | H | B:LEU  81 |  |  | |  |  | |
| H | C:ASP  85 | ||| | H | B:LYS  82 | ||| | H | C:ASP  85 | ||| | H | B:LYS  82 | ||| | H | C:ASP  85 | ||| | H | B:LYS  82 | ||| | H | C:ASP  85 | ||| | H | B:LYS  82 | ||| | H | C:ASP  85 | ||| | H | C:ASP  85 |
| H | C:LEU  86 | ||| | H | B:GLY  83 | ||| | H | C:LEU  86 | ||| | H | B:GLY  83 | ||| | H | C:LEU  86 | ||| | H | B:GLY  83 | ||| | H | C:LEU  86 | ||| | H | B:GLY  83 | ||| | H | C:LEU  86 | ||| | H | C:LEU  86 |
| H | C:HIS  87 | ||| | H | B:THR  84 | ||| | H | C:HIS  87 | ||| | H | B:THR  84 | ||| | H | C:HIS  87 | ||| | H | B:THR  84 | ||| | H | C:HIS  87 | ||| | H | B:THR  84 | ||| | H | C:HIS  87 | ||| | H | C:HIS  87 |
| H | C:ALA  88 | ||| | H | B:PHE  85 | ||| | H | C:ALA  88 | ||| | H | B:PHE  85 | ||| | H | C:ALA  88 | ||| | H | B:PHE  85 | ||| | H | C:ALA  88 | ||| | H | B:PHE  85 | ||| | H | C:ALA  88 | ||| | H | C:ALA  88 |
| H | C:HIS  89 | ||| | H | B:ALA  86 | ||| | H | C:HIS  89 | ||| | H | B:ALA  86 | ||| | H | C:HIS  89 | ||| | H | B:ALA  86 | ||| | H | C:HIS  89 | ||| | H | B:ALA  86 | ||| | H | C:HIS  89 | ||| | H | C:HIS  89 |
|  | C:LYS  90 | ||| | H | B:THR  87 | ||| | H | C:LYS  90 | ||| | H | B:THR  87 | ||| | H | C:LYS  90 | ||| | H | B:THR  87 | ||| | H | C:LYS  90 | ||| | H | B:THR  87 | ||| | H | C:LYS  90 | ||| | H | C:LYS  90 |
|  | C:LEU  91 | ||| | H | B:LEU  88 | ||| |  | C:LEU  91 | ||| | H | B:LEU  88 | ||| |  | C:LEU  91 | ||| | H | B:LEU  88 | ||| |  | C:LEU  91 | ||| | H | B:LEU  88 | ||| |  | C:LEU  91 | ||| |  | C:LEU  91 |
|  | C:ARG  92 | ||| | H | B:SER  89 | ||| |  | C:ARG  92 | ||| | H | B:SER  89 | ||| |  | C:ARG  92 | ||| | H | B:SER  89 | ||| |  | C:ARG  92 | ||| | H | B:SER  89 | ||| |  | C:ARG  92 | ||| |  | C:ARG  92 |
|  | C:VAL  93 | ||| | H | B:GLU  90 | ||| |  | C:VAL  93 | ||| | H | B:GLU  90 | ||| |  | C:VAL  93 | ||| | H | B:GLU  90 | ||| |  | C:VAL  93 | ||| | H | B:GLU  90 | ||| |  | C:VAL  93 | ||| |  | C:VAL  93 |
|  | C:ASP  94 | ||| | H | B:LEU  91 | ||| |  | C:ASP  94 | ||| | H | B:LEU  91 | ||| |  | C:ASP  94 | ||| | H | B:LEU  91 | ||| |  | C:ASP  94 | ||| | H | B:LEU  91 | ||| |  | C:ASP  94 | ||| |  | C:ASP  94 |
| H | C:PRO  95 | ||| | H | B:HIS  92 | ||| | H | C:PRO  95 | ||| | H | B:HIS  92 | ||| | H | C:PRO  95 | ||| | H | B:HIS  92 | ||| | H | C:PRO  95 | ||| | H | B:HIS  92 | ||| | H | C:PRO  95 | ||| | H | C:PRO  95 |
| H | C:VAL  96 | ||| | H | B:CYS  93 | ||| | H | C:VAL  96 | ||| | H | B:CYS  93 | ||| | H | C:VAL  96 | ||| | H | B:CYS  93 | ||| | H | C:VAL  96 | ||| | H | B:CYS  93 | ||| | H | C:VAL  96 | ||| | H | C:VAL  96 |
|  | |  | H | B:ASP  94 |  |  | |  | H | B:ASP  94 |  |  | |  | H | B:ASP  94 |  |  | |  | H | B:ASP  94 |  |  | |  |  | |
|  | |  | H | B:LYS  95 |  |  | |  | H | B:LYS  95 |  |  | |  | H | B:LYS  95 |  |  | |  | H | B:LYS  95 |  |  | |  |  | |
|  | |  |  | B:LEU  96 |  |  | |  |  | B:LEU  96 |  |  | |  |  | B:LEU  96 |  |  | |  |  | B:LEU  96 |  |  | |  |  | |
| H | C:ASN  97 | ||| |  | B:HIS  97 | ||| | H | C:ASN  97 | ||| |  | B:HIS  97 | ||| | H | C:ASN  97 | ||| |  | B:HIS  97 | ||| | H | C:ASN  97 | ||| |  | B:HIS  97 | ||| | H | C:ASN  97 | ||| | H | C:ASN  97 |
| H | C:PHE  98 | ||| |  | B:VAL  98 | ||| | H | C:PHE  98 | ||| |  | B:VAL  98 | ||| | H | C:PHE  98 | ||| |  | B:VAL  98 | ||| | H | C:PHE  98 | ||| |  | B:VAL  98 | ||| | H | C:PHE  98 | ||| | H | C:PHE  98 |
| H | C:LYS  99 | ||| |  | B:ASP  99 | ||| | H | C:LYS  99 | ||| |  | B:ASP  99 | ||| | H | C:LYS  99 | ||| |  | B:ASP  99 | ||| | H | C:LYS  99 | ||| |  | B:ASP  99 | ||| | H | C:LYS  99 | ||| | H | C:LYS  99 |
| H | C:LEU 100 | ||| | H | B:PRO 100 | ||| | H | C:LEU 100 | ||| | H | B:PRO 100 | ||| | H | C:LEU 100 | ||| | H | B:PRO 100 | ||| | H | C:LEU 100 | ||| | H | B:PRO 100 | ||| | H | C:LEU 100 | ||| | H | C:LEU 100 |
| H | C:LEU 101 | ||| | H | B:GLU 101 | ||| | H | C:LEU 101 | ||| | H | B:GLU 101 | ||| | H | C:LEU 101 | ||| | H | B:GLU 101 | ||| | H | C:LEU 101 | ||| | H | B:GLU 101 | ||| | H | C:LEU 101 | ||| | H | C:LEU 101 |
| H | C:SER 102 | ||| | H | B:ASN 102 | ||| | H | C:SER 102 | ||| | H | B:ASN 102 | ||| | H | C:SER 102 | ||| | H | B:ASN 102 | ||| | H | C:SER 102 | ||| | H | B:ASN 102 | ||| | H | C:SER 102 | ||| | H | C:SER 102 |
| H | C:HIS 103 | ||| | H | B:PHE 103 | ||| | H | C:HIS 103 | ||| | H | B:PHE 103 | ||| | H | C:HIS 103 | ||| | H | B:PHE 103 | ||| | H | C:HIS 103 | ||| | H | B:PHE 103 | ||| | H | C:HIS 103 | ||| | H | C:HIS 103 |
| H | C:CYS 104 | ||| | H | B:ARG 104 | ||| | H | C:CYS 104 | ||| | H | B:ARG 104 | ||| | H | C:CYS 104 | ||| | H | B:ARG 104 | ||| | H | C:CYS 104 | ||| | H | B:ARG 104 | ||| | H | C:CYS 104 | ||| | H | C:CYS 104 |
| H | C:LEU 105 | ||| | H | B:LEU 105 | ||| | H | C:LEU 105 | ||| | H | B:LEU 105 | ||| | H | C:LEU 105 | ||| | H | B:LEU 105 | ||| | H | C:LEU 105 | ||| | H | B:LEU 105 | ||| | H | C:LEU 105 | ||| | H | C:LEU 105 |
| H | C:LEU 106 | ||| | H | B:LEU 106 | ||| | H | C:LEU 106 | ||| | H | B:LEU 106 | ||| | H | C:LEU 106 | ||| | H | B:LEU 106 | ||| | H | C:LEU 106 | ||| | H | B:LEU 106 | ||| | H | C:LEU 106 | ||| | H | C:LEU 106 |
| H | C:VAL 107 | ||| | H | B:GLY 107 | ||| | H | C:VAL 107 | ||| | H | B:GLY 107 | ||| | H | C:VAL 107 | ||| | H | B:GLY 107 | ||| | H | C:VAL 107 | ||| | H | B:GLY 107 | ||| | H | C:VAL 107 | ||| | H | C:VAL 107 |
| H | C:THR 108 | ||| | H | B:ASN 108 | ||| | H | C:THR 108 | ||| | H | B:ASN 108 | ||| | H | C:THR 108 | ||| | H | B:ASN 108 | ||| | H | C:THR 108 | ||| | H | B:ASN 108 | ||| | H | C:THR 108 | ||| | H | C:THR 108 |
| H | C:LEU 109 | ||| | H | B:VAL 109 | ||| | H | C:LEU 109 | ||| | H | B:VAL 109 | ||| | H | C:LEU 109 | ||| | H | B:VAL 109 | ||| | H | C:LEU 109 | ||| | H | B:VAL 109 | ||| | H | C:LEU 109 | ||| | H | C:LEU 109 |
| H | C:ALA 110 | ||| | H | B:LEU 110 | ||| | H | C:ALA 110 | ||| | H | B:LEU 110 | ||| | H | C:ALA 110 | ||| | H | B:LEU 110 | ||| | H | C:ALA 110 | ||| | H | B:LEU 110 | ||| | H | C:ALA 110 | ||| | H | C:ALA 110 |
| H | C:ALA 111 | ||| | H | B:VAL 111 | ||| | H | C:ALA 111 | ||| | H | B:VAL 111 | ||| | H | C:ALA 111 | ||| | H | B:VAL 111 | ||| | H | C:ALA 111 | ||| | H | B:VAL 111 | ||| | H | C:ALA 111 | ||| | H | C:ALA 111 |
| H | C:HIS 112 | ||| | H | B:CYS 112 | ||| | H | C:HIS 112 | ||| | H | B:CYS 112 | ||| | H | C:HIS 112 | ||| | H | B:CYS 112 | ||| | H | C:HIS 112 | ||| | H | B:CYS 112 | ||| | H | C:HIS 112 | ||| | H | C:HIS 112 |
| H | C:LEU 113 | ||| | H | B:VAL 113 | ||| | H | C:LEU 113 | ||| | H | B:VAL 113 | ||| | H | C:LEU 113 | ||| | H | B:VAL 113 | ||| | H | C:LEU 113 | ||| | H | B:VAL 113 | ||| | H | C:LEU 113 | ||| | H | C:LEU 113 |
|  | C:PRO 114 | ||| | H | B:LEU 114 | ||| |  | C:PRO 114 | ||| | H | B:LEU 114 | ||| |  | C:PRO 114 | ||| | H | B:LEU 114 | ||| |  | C:PRO 114 | ||| | H | B:LEU 114 | ||| |  | C:PRO 114 | ||| |  | C:PRO 114 |
|  | C:ALA 115 | ||| | H | B:ALA 115 | ||| |  | C:ALA 115 | ||| | H | B:ALA 115 | ||| |  | C:ALA 115 | ||| | H | B:ALA 115 | ||| |  | C:ALA 115 | ||| | H | B:ALA 115 | ||| |  | C:ALA 115 | ||| |  | C:ALA 115 |
|  | C:GLU 116 | ||| | H | B:HIS 116 | ||| |  | C:GLU 116 | ||| | H | B:HIS 116 | ||| |  | C:GLU 116 | ||| | H | B:HIS 116 | ||| |  | C:GLU 116 | ||| | H | B:HIS 116 | ||| |  | C:GLU 116 | ||| |  | C:GLU 116 |
|  | C:PHE 117 | ||| | H | B:HIS 117 | ||| |  | C:PHE 117 | ||| | H | B:HIS 117 | ||| |  | C:PHE 117 | ||| | H | B:HIS 117 | ||| |  | C:PHE 117 | ||| | H | B:HIS 117 | ||| |  | C:PHE 117 | ||| |  | C:PHE 117 |
| H | C:THR 118 | ||| | H | B:PHE 118 | ||| | H | C:THR 118 | ||| | H | B:PHE 118 | ||| | H | C:THR 118 | ||| | H | B:PHE 118 | ||| | H | C:THR 118 | ||| | H | B:PHE 118 | ||| | H | C:THR 118 | ||| | H | C:THR 118 |
| H | C:PRO 119 | ||| | H | B:GLY 119 | ||| | H | C:PRO 119 | ||| | H | B:GLY 119 | ||| | H | C:PRO 119 | ||| | H | B:GLY 119 | ||| | H | C:PRO 119 | ||| | H | B:GLY 119 | ||| | H | C:PRO 119 | ||| | H | C:PRO 119 |
| H | C:ALA 120 | ||| |  | B:LYS 120 | ||| | H | C:ALA 120 | ||| |  | B:LYS 120 | ||| | H | C:ALA 120 | ||| |  | B:LYS 120 | ||| | H | C:ALA 120 | ||| |  | B:LYS 120 | ||| | H | C:ALA 120 | ||| | H | C:ALA 120 |
| H | C:VAL 121 | ||| |  | B:GLU 121 | ||| | H | C:VAL 121 | ||| |  | B:GLU 121 | ||| | H | C:VAL 121 | ||| |  | B:GLU 121 | ||| | H | C:VAL 121 | ||| |  | B:GLU 121 | ||| | H | C:VAL 121 | ||| | H | C:VAL 121 |
| H | C:HIS 122 | ||| |  | B:PHE 122 | ||| | H | C:HIS 122 | ||| |  | B:PHE 122 | ||| | H | C:HIS 122 | ||| |  | B:PHE 122 | ||| | H | C:HIS 122 | ||| |  | B:PHE 122 | ||| | H | C:HIS 122 | ||| | H | C:HIS 122 |
| H | C:ALA 123 | ||| | H | B:THR 123 | ||| | H | C:ALA 123 | ||| | H | B:THR 123 | ||| | H | C:ALA 123 | ||| | H | B:THR 123 | ||| | H | C:ALA 123 | ||| | H | B:THR 123 | ||| | H | C:ALA 123 | ||| | H | C:ALA 123 |
| H | C:SER 124 | ||| | H | B:PRO 124 | ||| | H | C:SER 124 | ||| | H | B:PRO 124 | ||| | H | C:SER 124 | ||| | H | B:PRO 124 | ||| | H | C:SER 124 | ||| | H | B:PRO 124 | ||| | H | C:SER 124 | ||| | H | C:SER 124 |
| H | C:LEU 125 | ||| | H | B:PRO 125 | ||| | H | C:LEU 125 | ||| | H | B:PRO 125 | ||| | H | C:LEU 125 | ||| | H | B:PRO 125 | ||| | H | C:LEU 125 | ||| | H | B:PRO 125 | ||| | H | C:LEU 125 | ||| | H | C:LEU 125 |
| H | C:ASP 126 | ||| | H | B:VAL 126 | ||| | H | C:ASP 126 | ||| | H | B:VAL 126 | ||| | H | C:ASP 126 | ||| | H | B:VAL 126 | ||| | H | C:ASP 126 | ||| | H | B:VAL 126 | ||| | H | C:ASP 126 | ||| | H | C:ASP 126 |
| H | C:LYS 127 | ||| | H | B:GLN 127 | ||| | H | C:LYS 127 | ||| | H | B:GLN 127 | ||| | H | C:LYS 127 | ||| | H | B:GLN 127 | ||| | H | C:LYS 127 | ||| | H | B:GLN 127 | ||| | H | C:LYS 127 | ||| | H | C:LYS 127 |
| H | C:PHE 128 | ||| | H | B:ALA 128 | ||| | H | C:PHE 128 | ||| | H | B:ALA 128 | ||| | H | C:PHE 128 | ||| | H | B:ALA 128 | ||| | H | C:PHE 128 | ||| | H | B:ALA 128 | ||| | H | C:PHE 128 | ||| | H | C:PHE 128 |
| H | C:LEU 129 | ||| | H | B:ALA 129 | ||| | H | C:LEU 129 | ||| | H | B:ALA 129 | ||| | H | C:LEU 129 | ||| | H | B:ALA 129 | ||| | H | C:LEU 129 | ||| | H | B:ALA 129 | ||| | H | C:LEU 129 | ||| | H | C:LEU 129 |
| H | C:ALA 130 | ||| | H | B:TYR 130 | ||| | H | C:ALA 130 | ||| | H | B:TYR 130 | ||| | H | C:ALA 130 | ||| | H | B:TYR 130 | ||| | H | C:ALA 130 | ||| | H | B:TYR 130 | ||| | H | C:ALA 130 | ||| | H | C:ALA 130 |
| H | C:SER 131 | ||| | H | B:GLN 131 | ||| | H | C:SER 131 | ||| | H | B:GLN 131 | ||| | H | C:SER 131 | ||| | H | B:GLN 131 | ||| | H | C:SER 131 | ||| | H | B:GLN 131 | ||| | H | C:SER 131 | ||| | H | C:SER 131 |
| H | C:VAL 132 | ||| | H | B:LYS 132 | ||| | H | C:VAL 132 | ||| | H | B:LYS 132 | ||| | H | C:VAL 132 | ||| | H | B:LYS 132 | ||| | H | C:VAL 132 | ||| | H | B:LYS 132 | ||| | H | C:VAL 132 | ||| | H | C:VAL 132 |
| H | C:SER 133 | ||| | H | B:VAL 133 | ||| | H | C:SER 133 | ||| | H | B:VAL 133 | ||| | H | C:SER 133 | ||| | H | B:VAL 133 | ||| | H | C:SER 133 | ||| | H | B:VAL 133 | ||| | H | C:SER 133 | ||| | H | C:SER 133 |
| H | C:THR 134 | ||| | H | B:VAL 134 | ||| | H | C:THR 134 | ||| | H | B:VAL 134 | ||| | H | C:THR 134 | ||| | H | B:VAL 134 | ||| | H | C:THR 134 | ||| | H | B:VAL 134 | ||| | H | C:THR 134 | ||| | H | C:THR 134 |
| H | C:VAL 135 | ||| | H | B:ALA 135 | ||| | H | C:VAL 135 | ||| | H | B:ALA 135 | ||| | H | C:VAL 135 | ||| | H | B:ALA 135 | ||| | H | C:VAL 135 | ||| | H | B:ALA 135 | ||| | H | C:VAL 135 | ||| | H | C:VAL 135 |
| H | C:LEU 136 | ||| | H | B:GLY 136 | ||| | H | C:LEU 136 | ||| | H | B:GLY 136 | ||| | H | C:LEU 136 | ||| | H | B:GLY 136 | ||| | H | C:LEU 136 | ||| | H | B:GLY 136 | ||| | H | C:LEU 136 | ||| | H | C:LEU 136 |
| AH | C:THR 137 | ||| | H | B:VAL 137 | ||| | H | C:THR 137 | ||| | H | B:VAL 137 | ||| | H | C:THR 137 | ||| | H | B:VAL 137 | ||| | H | C:THR 137 | ||| | H | B:VAL 137 | ||| | H | C:THR 137 | ||| | H | C:THR 137 |
|  | C:SER 138 | ||| | H | B:ALA 138 | ||| | H | C:SER 138 | ||| | H | B:ALA 138 | ||| |  | C:SER 138 | ||| | H | B:ALA 138 | ||| | H | C:SER 138 | ||| | H | B:ALA 138 | ||| |  | C:SER 138 | ||| |  | C:SER 138 |
|  | C:LYS 139 | ||| | H | B:ASN 139 | ||| |  | C:LYS 139 | ||| | H | B:ASN 139 | ||| |  | C:LYS 139 | ||| | H | B:ASN 139 | ||| |  | C:LYS 139 | ||| | H | B:ASN 139 | ||| |  | C:LYS 139 | ||| |  | C:LYS 139 |
|  | C:TYR 140 | ||| | H | B:ALA 140 | ||| |  | C:TYR 140 | ||| | H | B:ALA 140 | ||| |  | C:TYR 140 | ||| | H | B:ALA 140 | ||| |  | C:TYR 140 | ||| | H | B:ALA 140 | ||| |  | C:TYR 140 | ||| |  | C:TYR 140 |
|  | C:ARG 141 | ||| | H | B:LEU 141 | ||| |  | C:ARG 141 | ||| | H | B:LEU 141 | ||| |  | C:ARG 141 | ||| | H | B:LEU 141 | ||| |  | C:ARG 141 | ||| | H | B:LEU 141 | ||| |  | C:ARG 141 | ||| |  | C:ARG 141 |
|  | |  | H | B:ALA 142 |  |  | D:MET   1 |  | H | B:ALA 142 |  |  | D:MET   1 |  | H | B:ALA 142 |  |  | D:VAL   1 |  | H | B:ALA 142 |  |  | D:MET   1 |  |  | D:MET   1 |
|  | |  | H | B:HIS 143 |  |  | |  |  | B:HIS 143 |  |  | |  | H | B:HIS 143 |  |  | |  | H | B:HIS 143 |  |  | |  |  | |
|  | |  |  | B:LYS 144 |  |  | |  |  | B:LYS 144 |  |  | |  |  | B:LYS 144 |  |  | |  |  | B:LYS 144 |  |  | |  |  | |
|  | |  |  | B:TYR 145 |  |  | |  |  | B:TYR 145 |  |  | |  |  | B:TYR 145 |  |  | |  |  | B:TYR 145 |  |  | |  |  | |
|  | |  |  | B:HIS 146 |  |  | |  |  | B:HIS 146 |  |  | |  |  | B:HIS 146 |  |  | |  |  | B:HIS 146 |  |  | |  |  | |
|  | |  |  | D:MET   1 |  |  | |  |  | D:MET   1 |  |  | |  |  | D:MET   1 |  |  | |  |  | D:MET   1 |  |  | |  |  | |
|  | D:VAL   1 | ||| |  | D:HIS   2 | ||| |  | D:HIS   2 | ||| |  | D:HIS   2 | ||| |  | D:HIS   2 | ||| |  | D:HIS   2 | ||| |  | D:HIS   2 | ||| |  | D:HIS   2 | ||| |  | D:HIS   2 | ||| |  | D:HIS   2 |
|  | D:HIS   2 | ||| |  | D:LEU   3 | ||| |  | D:LEU   3 | ||| |  | D:LEU   3 | ||| |  | D:LEU   3 | ||| |  | D:LEU   3 | ||| |  | D:LEU   3 | ||| |  | D:LEU   3 | ||| |  | D:LEU   3 | ||| |  | D:LEU   3 |
|  | D:LEU   3 |  |  | |  |  | |  |  | |  |  | |  |  | |  |  | |  |  | |  |  | |  |  | |
| H | D:THR   4 | ||| | H | D:THR   4 | ||| | H | D:THR   4 | ||| | H | D:THR   4 | ||| | H | D:THR   4 | ||| | H | D:THR   4 | ||| | H | D:THR   4 | ||| | H | D:THR   4 | ||| | H | D:THR   4 | ||| | H | D:THR   4 |
| H | D:PRO   5 | ||| | H | D:PRO   5 | ||| | H | D:PRO   5 | ||| | H | D:PRO   5 | ||| | H | D:PRO   5 | ||| | H | D:PRO   5 | ||| | H | D:PRO   5 | ||| | H | D:PRO   5 | ||| | H | D:PRO   5 | ||| | H | D:PRO   5 |
| H | D:GLU   6 | ||| | H | D:GLU   6 | ||| | H | D:GLU   6 | ||| | H | D:GLU   6 | ||| | H | D:GLU   6 | ||| | H | D:GLU   6 | ||| | H | D:GLU   6 | ||| | H | D:GLU   6 | ||| | H | D:GLU   6 | ||| | H | D:GLU   6 |
| H | D:GLU   7 | ||| | H | D:GLU   7 | ||| | H | D:GLU   7 | ||| | H | D:GLU   7 | ||| | H | D:GLU   7 | ||| | H | D:GLU   7 | ||| | H | D:GLU   7 | ||| | H | D:GLU   7 | ||| | H | D:GLU   7 | ||| | H | D:GLU   7 |
| H | D:LYS   8 | ||| | H | D:LYS   8 | ||| | H | D:LYS   8 | ||| | H | D:LYS   8 | ||| | H | D:LYS   8 | ||| | H | D:LYS   8 | ||| | H | D:LYS   8 | ||| | H | D:LYS   8 | ||| | H | D:LYS   8 | ||| | H | D:LYS   8 |
| H | D:SER   9 | ||| | H | D:SER   9 | ||| | H | D:SER   9 | ||| | H | D:SER   9 | ||| | H | D:SER   9 | ||| | H | D:SER   9 | ||| | H | D:SER   9 | ||| | H | D:SER   9 | ||| | H | D:SER   9 | ||| | H | D:SER   9 |
| H | D:ALA  10 | ||| | H | D:ALA  10 | ||| | H | D:ALA  10 | ||| | H | D:ALA  10 | ||| | H | D:ALA  10 | ||| | H | D:ALA  10 | ||| | H | D:ALA  10 | ||| | H | D:ALA  10 | ||| | H | D:ALA  10 | ||| | H | D:ALA  10 |
| H | D:VAL  11 | ||| | H | D:VAL  11 | ||| | H | D:VAL  11 | ||| | H | D:VAL  11 | ||| | H | D:VAL  11 | ||| | H | D:VAL  11 | ||| | H | D:VAL  11 | ||| | H | D:VAL  11 | ||| | H | D:VAL  11 | ||| | H | D:VAL  11 |
| H | D:THR  12 | ||| | H | D:THR  12 | ||| | H | D:THR  12 | ||| | H | D:THR  12 | ||| | H | D:THR  12 | ||| | H | D:THR  12 | ||| | H | D:THR  12 | ||| | H | D:THR  12 | ||| | H | D:THR  12 | ||| | H | D:THR  12 |
| H | D:ALA  13 | ||| | H | D:ALA  13 | ||| | H | D:ALA  13 | ||| | H | D:ALA  13 | ||| | H | D:ALA  13 | ||| | H | D:ALA  13 | ||| | H | D:ALA  13 | ||| | H | D:ALA  13 | ||| | H | D:ALA  13 | ||| | H | D:ALA  13 |
| H | D:LEU  14 | ||| | H | D:LEU  14 | ||| | H | D:LEU  14 | ||| | H | D:LEU  14 | ||| | H | D:LEU  14 | ||| | H | D:LEU  14 | ||| | H | D:LEU  14 | ||| | H | D:LEU  14 | ||| | H | D:LEU  14 | ||| | H | D:LEU  14 |
| H | D:TRP  15 | ||| | H | D:TRP  15 | ||| | H | D:TRP  15 | ||| | H | D:TRP  15 | ||| | H | D:TRP  15 | ||| | H | D:TRP  15 | ||| | H | D:TRP  15 | ||| | H | D:TRP  15 | ||| | H | D:TRP  15 | ||| | H | D:TRP  15 |
| H | D:GLY  16 | ||| | H | D:GLY  16 | ||| | H | D:GLY  16 | ||| | H | D:GLY  16 | ||| | H | D:GLY  16 | ||| | H | D:GLY  16 | ||| | H | D:GLY  16 | ||| | H | D:GLY  16 | ||| | H | D:GLY  16 | ||| | H | D:GLY  16 |
| H | D:LYS  17 | ||| |  | D:LYS  17 | ||| |  | D:LYS  17 | ||| |  | D:LYS  17 | ||| |  | D:LYS  17 | ||| |  | D:LYS  17 | ||| |  | D:LYS  17 | ||| |  | D:LYS  17 | ||| |  | D:LYS  17 | ||| |  | D:LYS  17 |
|  | D:VAL  18 | ||| |  | D:VAL  18 | ||| |  | D:VAL  18 | ||| |  | D:VAL  18 | ||| |  | D:VAL  18 | ||| |  | D:VAL  18 | ||| |  | D:VAL  18 | ||| |  | D:VAL  18 | ||| |  | D:VAL  18 | ||| |  | D:VAL  18 |
|  | D:ASN  19 |  |  | |  |  | |  |  | |  |  | |  |  | |  |  | |  |  | |  |  | |  |  | |
|  | D:VAL  20 | ||| | H | D:ASN  19 | ||| | H | D:ASN  19 | ||| | H | D:ASN  19 | ||| | H | D:ASN  19 | ||| | H | D:ASN  19 | ||| | H | D:ASN  19 | ||| | H | D:ASN  19 | ||| | H | D:ASN  19 | ||| | H | D:ASN  19 |
|  | |  | H | D:VAL  20 |  | H | D:VAL  20 |  | H | D:VAL  20 |  | H | D:VAL  20 |  | H | D:VAL  20 |  | H | D:VAL  20 |  | H | D:VAL  20 |  | H | D:VAL  20 |  | H | D:VAL  20 |
|  | D:ASP  21 | ||| | H | D:ASP  21 | ||| | H | D:ASP  21 | ||| | H | D:ASP  21 | ||| | H | D:ASP  21 | ||| | H | D:ASP  21 | ||| | H | D:ASP  21 | ||| | H | D:ASP  21 | ||| | H | D:ASP  21 | ||| | H | D:ASP  21 |
| H | D:GLU  22 | ||| | H | D:GLU  22 | ||| | H | D:GLU  22 | ||| | H | D:GLU  22 | ||| | H | D:GLU  22 | ||| | H | D:GLU  22 | ||| | H | D:GLU  22 | ||| | H | D:GLU  22 | ||| | H | D:GLU  22 | ||| | H | D:GLU  22 |
| H | D:VAL  23 | ||| | H | D:VAL  23 | ||| | H | D:VAL  23 | ||| | H | D:VAL  23 | ||| | H | D:VAL  23 | ||| | H | D:VAL  23 | ||| | H | D:VAL  23 | ||| | H | D:VAL  23 | ||| | H | D:VAL  23 | ||| | H | D:VAL  23 |
| H | D:GLY  24 | ||| | H | D:GLY  24 | ||| | H | D:GLY  24 | ||| | H | D:GLY  24 | ||| | H | D:GLY  24 | ||| | H | D:GLY  24 | ||| | H | D:GLY  24 | ||| | H | D:GLY  24 | ||| | H | D:GLY  24 | ||| | H | D:GLY  24 |
| H | D:GLY  25 | ||| | H | D:GLY  25 | ||| | H | D:GLY  25 | ||| | H | D:GLY  25 | ||| | H | D:GLY  25 | ||| | H | D:GLY  25 | ||| | H | D:GLY  25 | ||| | H | D:GLY  25 | ||| | H | D:GLY  25 | ||| | H | D:GLY  25 |
| H | D:GLU  26 | ||| | H | D:GLU  26 | ||| | H | D:GLU  26 | ||| | H | D:GLU  26 | ||| | H | D:GLU  26 | ||| | H | D:GLU  26 | ||| | H | D:GLU  26 | ||| | H | D:GLU  26 | ||| | H | D:GLU  26 | ||| | H | D:GLU  26 |
| H | D:ALA  27 | ||| | H | D:ALA  27 | ||| | H | D:ALA  27 | ||| | H | D:ALA  27 | ||| | H | D:ALA  27 | ||| | H | D:ALA  27 | ||| | H | D:ALA  27 | ||| | H | D:ALA  27 | ||| | H | D:ALA  27 | ||| | H | D:ALA  27 |
| H | D:LEU  28 | ||| | H | D:LEU  28 | ||| | H | D:LEU  28 | ||| | H | D:LEU  28 | ||| | H | D:LEU  28 | ||| | H | D:LEU  28 | ||| | H | D:LEU  28 | ||| | H | D:LEU  28 | ||| | H | D:LEU  28 | ||| | H | D:LEU  28 |
| H | D:GLY  29 | ||| | H | D:GLY  29 | ||| | H | D:GLY  29 | ||| | H | D:GLY  29 | ||| | H | D:GLY  29 | ||| | H | D:GLY  29 | ||| | H | D:GLY  29 | ||| | H | D:GLY  29 | ||| | H | D:GLY  29 | ||| | H | D:GLY  29 |
| H | D:ARG  30 | ||| | H | D:ARG  30 | ||| | H | D:ARG  30 | ||| | H | D:ARG  30 | ||| | H | D:ARG  30 | ||| | H | D:ARG  30 | ||| | H | D:ARG  30 | ||| | H | D:ARG  30 | ||| | H | D:ARG  30 | ||| | H | D:ARG  30 |
| H | D:LEU  31 | ||| | H | D:LEU  31 | ||| | H | D:LEU  31 | ||| | H | D:LEU  31 | ||| | H | D:LEU  31 | ||| | H | D:LEU  31 | ||| | H | D:LEU  31 | ||| | H | D:LEU  31 | ||| | H | D:LEU  31 | ||| | H | D:LEU  31 |
| H | D:LEU  32 | ||| | H | D:LEU  32 | ||| | H | D:LEU  32 | ||| | H | D:LEU  32 | ||| | H | D:LEU  32 | ||| | H | D:LEU  32 | ||| | H | D:LEU  32 | ||| | H | D:LEU  32 | ||| | H | D:LEU  32 | ||| | H | D:LEU  32 |
| H | D:VAL  33 | ||| | H | D:VAL  33 | ||| | H | D:VAL  33 | ||| | H | D:VAL  33 | ||| | H | D:VAL  33 | ||| | H | D:VAL  33 | ||| | H | D:VAL  33 | ||| | H | D:VAL  33 | ||| | H | D:VAL  33 | ||| | H | D:VAL  33 |
| H | D:VAL  34 | ||| | H | D:VAL  34 | ||| | H | D:VAL  34 | ||| | H | D:VAL  34 | ||| | H | D:VAL  34 | ||| | H | D:VAL  34 | ||| | H | D:VAL  34 | ||| | H | D:VAL  34 | ||| | H | D:VAL  34 | ||| | H | D:VAL  34 |
| H | D:TYR  35 | ||| | H | D:TYR  35 | ||| | H | D:TYR  35 | ||| | H | D:TYR  35 | ||| | H | D:TYR  35 | ||| | H | D:TYR  35 | ||| | H | D:TYR  35 | ||| | H | D:TYR  35 | ||| | H | D:TYR  35 | ||| | H | D:TYR  35 |
| H | D:PRO  36 | ||| | H | D:PRO  36 | ||| | H | D:PRO  36 | ||| | H | D:PRO  36 | ||| | H | D:PRO  36 | ||| | H | D:PRO  36 | ||| | H | D:PRO  36 | ||| | H | D:PRO  36 | ||| | H | D:PRO  36 | ||| | H | D:PRO  36 |
| H | D:TRP  37 | ||| | H | D:ALA  37 | ||| | H | D:ALA  37 | ||| | H | D:GLU  37 | ||| | H | D:TYR  37 | ||| | H | D:TYR  37 | ||| | H | D:TRP  37 | ||| | H | D:GLY  37 | ||| | H | D:TRP  37 | ||| | H | D:TRP  37 |
| H | D:THR  38 | ||| | H | D:THR  38 | ||| | H | D:THR  38 | ||| | H | D:THR  38 | ||| | H | D:THR  38 | ||| | H | D:THR  38 | ||| | H | D:THR  38 | ||| | H | D:THR  38 | ||| | H | D:THR  38 | ||| | H | D:THR  38 |
| H | D:GLN  39 | ||| | H | D:GLN  39 | ||| | H | D:GLN  39 | ||| | H | D:GLN  39 | ||| | H | D:GLN  39 | ||| | H | D:GLN  39 | ||| | H | D:GLN  39 | ||| | H | D:GLN  39 | ||| | H | D:GLN  39 | ||| | H | D:GLN  39 |
| H | D:ARG  40 | ||| | H | D:ARG  40 | ||| | H | D:ARG  40 | ||| | H | D:ARG  40 | ||| | H | D:ARG  40 | ||| | H | D:ARG  40 | ||| | H | D:ARG  40 | ||| | H | D:ARG  40 | ||| | H | D:ARG  40 | ||| | H | D:ARG  40 |
| H | D:PHE  41 | ||| | H | D:PHE  41 | ||| | H | D:PHE  41 | ||| | H | D:PHE  41 | ||| | H | D:PHE  41 | ||| | H | D:PHE  41 | ||| | H | D:PHE  41 | ||| | H | D:PHE  41 | ||| | H | D:PHE  41 | ||| | H | D:PHE  41 |
| H | D:PHE  42 | ||| | H | D:PHE  42 | ||| | H | D:PHE  42 | ||| | H | D:PHE  42 | ||| | H | D:PHE  42 | ||| | H | D:PHE  42 | ||| | H | D:PHE  42 | ||| | H | D:PHE  42 | ||| | H | D:PHE  42 | ||| | H | D:PHE  42 |
| H | D:GLU  43 | ||| | H | D:GLU  43 | ||| | H | D:GLU  43 | ||| | H | D:GLU  43 | ||| | H | D:GLU  43 | ||| | H | D:GLU  43 | ||| | H | D:GLU  43 | ||| | H | D:GLU  43 | ||| | H | D:GLU  43 | ||| | H | D:GLU  43 |
| H | D:SER  44 |  |  | |  |  | |  |  | |  |  | |  |  | |  |  | |  |  | |  |  | |  |  | |
| H | D:PHE  45 | ||| | H | D:SER  44 | ||| | H | D:SER  44 | ||| | H | D:SER  44 | ||| | H | D:SER  44 | ||| | H | D:SER  44 | ||| | H | D:SER  44 | ||| | H | D:SER  44 | ||| | H | D:SER  44 | ||| | H | D:SER  44 |
| H | D:GLY  46 | ||| | H | D:PHE  45 | ||| | H | D:PHE  45 | ||| | H | D:PHE  45 | ||| | H | D:PHE  45 | ||| | H | D:PHE  45 | ||| | H | D:PHE  45 | ||| | H | D:PHE  45 | ||| | H | D:PHE  45 | ||| | H | D:PHE  45 |
|  | D:ASP  47 | ||| | H | D:GLY  46 | ||| | H | D:GLY  46 | ||| | H | D:GLY  46 | ||| | H | D:GLY  46 | ||| | H | D:GLY  46 | ||| | H | D:GLY  46 | ||| | H | D:GLY  46 | ||| | H | D:GLY  46 | ||| | H | D:GLY  46 |
|  | D:LEU  48 | ||| |  | D:ASP  47 | ||| |  | D:ASP  47 | ||| |  | D:ASP  47 | ||| |  | D:ASP  47 | ||| |  | D:ASP  47 | ||| |  | D:ASP  47 | ||| |  | D:ASP  47 | ||| |  | D:ASP  47 | ||| |  | D:ASP  47 |
|  | D:SER  49 | ||| |  | D:LEU  48 | ||| |  | D:LEU  48 | ||| |  | D:LEU  48 | ||| |  | D:LEU  48 | ||| |  | D:LEU  48 | ||| |  | D:LEU  48 | ||| |  | D:LEU  48 | ||| |  | D:LEU  48 | ||| |  | D:LEU  48 |
|  | |  |  | D:SER  49 |  |  | D:SER  49 |  |  | D:SER  49 |  |  | D:SER  49 |  |  | D:SER  49 |  |  | D:SER  49 |  |  | D:SER  49 |  |  | D:SER  49 |  |  | D:SER  49 |
| H | D:THR  50 | ||| | H | D:THR  50 | ||| | H | D:THR  50 | ||| | H | D:THR  50 | ||| | H | D:THR  50 | ||| | H | D:THR  50 | ||| | H | D:THR  50 | ||| | H | D:THR  50 | ||| | H | D:THR  50 | ||| | H | D:THR  50 |
| H | D:PRO  51 | ||| | H | D:PRO  51 | ||| | H | D:PRO  51 | ||| | H | D:PRO  51 | ||| | H | D:PRO  51 | ||| | H | D:PRO  51 | ||| | H | D:PRO  51 | ||| | H | D:PRO  51 | ||| | H | D:PRO  51 | ||| | H | D:PRO  51 |
| H | D:ASP  52 | ||| | H | D:ASP  52 | ||| | H | D:ASP  52 | ||| | H | D:ASP  52 | ||| | H | D:ASP  52 | ||| | H | D:ASP  52 | ||| | H | D:ASP  52 | ||| | H | D:ASP  52 | ||| | H | D:ASP  52 | ||| | H | D:ASP  52 |
| H | D:ALA  53 | ||| | H | D:ALA  53 | ||| | H | D:ALA  53 | ||| | H | D:ALA  53 | ||| | H | D:ALA  53 | ||| | H | D:ALA  53 | ||| | H | D:ALA  53 | ||| | H | D:ALA  53 | ||| | H | D:ALA  53 | ||| | H | D:ALA  53 |
| H | D:VAL  54 | ||| | H | D:VAL  54 | ||| | H | D:VAL  54 | ||| | H | D:VAL  54 | ||| | H | D:VAL  54 | ||| | H | D:VAL  54 | ||| | H | D:VAL  54 | ||| | H | D:VAL  54 | ||| | H | D:VAL  54 | ||| | H | D:VAL  54 |
| H | D:MET  55 | ||| | H | D:MET  55 | ||| | H | D:MET  55 | ||| | H | D:MET  55 | ||| | H | D:MET  55 | ||| | H | D:MET  55 | ||| | H | D:MET  55 | ||| | H | D:MET  55 | ||| | H | D:MET  55 | ||| | H | D:MET  55 |
| H | D:GLY  56 | ||| | H | D:GLY  56 | ||| | H | D:GLY  56 | ||| | H | D:GLY  56 | ||| | H | D:GLY  56 | ||| | H | D:GLY  56 | ||| | H | D:GLY  56 | ||| | H | D:GLY  56 | ||| | H | D:GLY  56 | ||| | H | D:GLY  56 |
| H | D:ASN  57 | ||| | H | D:ASN  57 | ||| | H | D:ASN  57 | ||| | H | D:ASN  57 | ||| | H | D:ASN  57 | ||| | H | D:ASN  57 | ||| | H | D:ASN  57 | ||| | H | D:ASN  57 | ||| | H | D:ASN  57 | ||| | H | D:ASN  57 |
| H | D:PRO  58 | ||| | H | D:PRO  58 | ||| | H | D:PRO  58 | ||| | H | D:PRO  58 | ||| | H | D:PRO  58 | ||| | H | D:PRO  58 | ||| | H | D:PRO  58 | ||| | H | D:PRO  58 | ||| | H | D:PRO  58 | ||| | H | D:PRO  58 |
| H | D:LYS  59 | ||| | H | D:LYS  59 | ||| | H | D:LYS  59 | ||| | H | D:LYS  59 | ||| | H | D:LYS  59 | ||| | H | D:LYS  59 | ||| | H | D:LYS  59 | ||| | H | D:LYS  59 | ||| | H | D:LYS  59 | ||| | H | D:LYS  59 |
| H | D:VAL  60 | ||| | H | D:VAL  60 | ||| | H | D:VAL  60 | ||| | H | D:VAL  60 | ||| | H | D:VAL  60 | ||| | H | D:VAL  60 | ||| | H | D:VAL  60 | ||| | H | D:VAL  60 | ||| | H | D:VAL  60 | ||| | H | D:VAL  60 |
| H | D:LYS  61 | ||| | H | D:LYS  61 | ||| | H | D:LYS  61 | ||| | H | D:LYS  61 | ||| | H | D:LYS  61 | ||| | H | D:LYS  61 | ||| | H | D:LYS  61 | ||| | H | D:LYS  61 | ||| | H | D:LYS  61 | ||| | H | D:LYS  61 |
| H | D:ALA  62 | ||| | H | D:ALA  62 | ||| | H | D:ALA  62 | ||| | H | D:ALA  62 | ||| | H | D:ALA  62 | ||| | H | D:ALA  62 | ||| | H | D:ALA  62 | ||| | H | D:ALA  62 | ||| | H | D:ALA  62 | ||| | H | D:ALA  62 |
| H | D:HIS  63 | ||| | H | D:HIS  63 | ||| | H | D:HIS  63 | ||| | H | D:HIS  63 | ||| | H | D:HIS  63 | ||| | H | D:HIS  63 | ||| | H | D:HIS  63 | ||| | H | D:HIS  63 | ||| | H | D:HIS  63 | ||| | H | D:HIS  63 |
| H | D:GLY  64 | ||| | H | D:GLY  64 | ||| | H | D:GLY  64 | ||| | H | D:GLY  64 | ||| | H | D:GLY  64 | ||| | H | D:GLY  64 | ||| | H | D:GLY  64 | ||| | H | D:GLY  64 | ||| | H | D:GLY  64 | ||| | H | D:GLY  64 |
| H | D:LYS  65 | ||| | H | D:LYS  65 | ||| | H | D:LYS  65 | ||| | H | D:LYS  65 | ||| | H | D:LYS  65 | ||| | H | D:LYS  65 | ||| | H | D:LYS  65 | ||| | H | D:LYS  65 | ||| | H | D:LYS  65 | ||| | H | D:LYS  65 |
| H | D:LYS  66 | ||| | H | D:LYS  66 | ||| | H | D:LYS  66 | ||| | H | D:LYS  66 | ||| | H | D:LYS  66 | ||| | H | D:LYS  66 | ||| | H | D:LYS  66 | ||| | H | D:LYS  66 | ||| | H | D:LYS  66 | ||| | H | D:LYS  66 |
| H | D:VAL  67 | ||| | H | D:VAL  67 | ||| | H | D:VAL  67 | ||| | H | D:VAL  67 | ||| | H | D:VAL  67 | ||| | H | D:VAL  67 | ||| | H | D:VAL  67 | ||| | H | D:VAL  67 | ||| | H | D:VAL  67 | ||| | H | D:VAL  67 |
| H | D:LEU  68 | ||| | H | D:LEU  68 | ||| | H | D:LEU  68 | ||| | H | D:LEU  68 | ||| | H | D:LEU  68 | ||| | H | D:LEU  68 | ||| | H | D:LEU  68 | ||| | H | D:LEU  68 | ||| | H | D:LEU  68 | ||| | H | D:LEU  68 |
| H | D:GLY  69 | ||| | H | D:GLY  69 | ||| | H | D:GLY  69 | ||| | H | D:GLY  69 | ||| | H | D:GLY  69 | ||| | H | D:GLY  69 | ||| | H | D:GLY  69 | ||| | H | D:GLY  69 | ||| | H | D:GLY  69 | ||| | H | D:GLY  69 |
| H | D:ALA  70 | ||| | H | D:ALA  70 | ||| | H | D:ALA  70 | ||| | H | D:ALA  70 | ||| | H | D:ALA  70 | ||| | H | D:ALA  70 | ||| | H | D:ALA  70 | ||| | H | D:ALA  70 | ||| | H | D:ALA  70 | ||| | H | D:ALA  70 |
| H | D:PHE  71 | ||| | H | D:PHE  71 | ||| | H | D:PHE  71 | ||| | H | D:PHE  71 | ||| | H | D:PHE  71 | ||| | H | D:PHE  71 | ||| | H | D:PHE  71 | ||| | H | D:PHE  71 | ||| | H | D:PHE  71 | ||| | H | D:PHE  71 |
| H | D:SER  72 | ||| | H | D:SER  72 | ||| | H | D:SER  72 | ||| | H | D:SER  72 | ||| | H | D:SER  72 | ||| | H | D:SER  72 | ||| | H | D:SER  72 | ||| | H | D:SER  72 | ||| | H | D:SER  72 | ||| | H | D:SER  72 |
| H | D:ASP  73 | ||| | H | D:ASP  73 | ||| | H | D:ASP  73 | ||| | H | D:ASP  73 | ||| | H | D:ASP  73 | ||| | H | D:ASP  73 | ||| | H | D:ASP  73 | ||| | H | D:ASP  73 | ||| | H | D:ASP  73 | ||| | H | D:ASP  73 |
| H | D:GLY  74 | ||| | H | D:GLY  74 | ||| | H | D:GLY  74 | ||| | H | D:GLY  74 | ||| | H | D:GLY  74 | ||| | H | D:GLY  74 | ||| | H | D:GLY  74 | ||| | H | D:GLY  74 | ||| | H | D:GLY  74 | ||| | H | D:GLY  74 |
| H | D:LEU  75 | ||| | H | D:LEU  75 | ||| | H | D:LEU  75 | ||| | H | D:LEU  75 | ||| | H | D:LEU  75 | ||| | H | D:LEU  75 | ||| | H | D:LEU  75 | ||| | H | D:LEU  75 | ||| | H | D:LEU  75 | ||| | H | D:LEU  75 |
| H | D:ALA  76 | ||| |  | D:ALA  76 | ||| | H | D:ALA  76 | ||| |  | D:ALA  76 | ||| |  | D:ALA  76 | ||| |  | D:ALA  76 | ||| | H | D:ALA  76 | ||| | H | D:ALA  76 | ||| |  | D:ALA  76 | ||| |  | D:ALA  76 |
| H | D:HIS  77 | ||| |  | D:HIS  77 | ||| |  | D:HIS  77 | ||| |  | D:HIS  77 | ||| |  | D:HIS  77 | ||| |  | D:HIS  77 | ||| |  | D:HIS  77 | ||| |  | D:HIS  77 | ||| |  | D:HIS  77 | ||| |  | D:HIS  77 |
|  | D:LEU  78 |  |  | |  |  | |  |  | |  |  | |  |  | |  |  | |  |  | |  |  | |  |  | |
|  | D:ASP  79 |  |  | |  |  | |  |  | |  |  | |  |  | |  |  | |  |  | |  |  | |  |  | |
| H | D:ASN  80 |  |  | |  |  | |  |  | |  |  | |  |  | |  |  | |  |  | |  |  | |  |  | |
| H | D:LEU  81 | ||| |  | D:LEU  78 | ||| |  | D:LEU  78 | ||| |  | D:LEU  78 | ||| |  | D:LEU  78 | ||| |  | D:LEU  78 | ||| |  | D:LEU  78 | ||| |  | D:LEU  78 | ||| |  | D:LEU  78 | ||| |  | D:LEU  78 |
| H | D:LYS  82 | ||| |  | D:ASP  79 | ||| |  | D:ASP  79 | ||| |  | D:ASP  79 | ||| |  | D:ASP  79 | ||| |  | D:ASP  79 | ||| |  | D:ASP  79 | ||| |  | D:ASP  79 | ||| |  | D:ASP  79 | ||| |  | D:ASP  79 |
| H | D:GLY  83 | ||| | H | D:ASN  80 | ||| | H | D:ASN  80 | ||| | H | D:ASN  80 | ||| | H | D:ASN  80 | ||| | H | D:ASN  80 | ||| | H | D:ASN  80 | ||| | H | D:ASN  80 | ||| | H | D:ASN  80 | ||| | H | D:ASN  80 |
| H | D:THR  84 | ||| | H | D:LEU  81 | ||| | H | D:LEU  81 | ||| | H | D:LEU  81 | ||| | H | D:LEU  81 | ||| | H | D:LEU  81 | ||| | H | D:LEU  81 | ||| | H | D:LEU  81 | ||| | H | D:LEU  81 | ||| | H | D:LEU  81 |
| H | D:PHE  85 | ||| | H | D:LYS  82 | ||| | H | D:LYS  82 | ||| | H | D:LYS  82 | ||| | H | D:LYS  82 | ||| | H | D:LYS  82 | ||| | H | D:LYS  82 | ||| | H | D:LYS  82 | ||| | H | D:LYS  82 | ||| | H | D:LYS  82 |
| H | D:ALA  86 | ||| | H | D:GLY  83 | ||| | H | D:GLY  83 | ||| | H | D:GLY  83 | ||| | H | D:GLY  83 | ||| | H | D:GLY  83 | ||| | H | D:GLY  83 | ||| | H | D:GLY  83 | ||| | H | D:GLY  83 | ||| | H | D:GLY  83 |
| H | D:THR  87 | ||| | H | D:THR  84 | ||| | H | D:THR  84 | ||| | H | D:THR  84 | ||| | H | D:THR  84 | ||| | H | D:THR  84 | ||| | H | D:THR  84 | ||| | H | D:THR  84 | ||| | H | D:THR  84 | ||| | H | D:THR  84 |
| H | D:LEU  88 | ||| | H | D:PHE  85 | ||| | H | D:PHE  85 | ||| | H | D:PHE  85 | ||| | H | D:PHE  85 | ||| | H | D:PHE  85 | ||| | H | D:PHE  85 | ||| | H | D:PHE  85 | ||| | H | D:PHE  85 | ||| | H | D:PHE  85 |
| H | D:SER  89 | ||| | H | D:ALA  86 | ||| | H | D:ALA  86 | ||| | H | D:ALA  86 | ||| | H | D:ALA  86 | ||| | H | D:ALA  86 | ||| | H | D:ALA  86 | ||| | H | D:ALA  86 | ||| | H | D:ALA  86 | ||| | H | D:ALA  86 |
| H | D:GLU  90 | ||| | H | D:THR  87 | ||| | H | D:THR  87 | ||| | H | D:THR  87 | ||| | H | D:THR  87 | ||| | H | D:THR  87 | ||| | H | D:THR  87 | ||| | H | D:THR  87 | ||| | H | D:THR  87 | ||| | H | D:THR  87 |
| H | D:LEU  91 | ||| | H | D:LEU  88 | ||| | H | D:LEU  88 | ||| | H | D:LEU  88 | ||| | H | D:LEU  88 | ||| | H | D:LEU  88 | ||| | H | D:LEU  88 | ||| | H | D:LEU  88 | ||| | H | D:LEU  88 | ||| | H | D:LEU  88 |
| H | D:HIS  92 | ||| | H | D:SER  89 | ||| | H | D:SER  89 | ||| | H | D:SER  89 | ||| | H | D:SER  89 | ||| | H | D:SER  89 | ||| | H | D:SER  89 | ||| | H | D:SER  89 | ||| | H | D:SER  89 | ||| | H | D:SER  89 |
| H | D:SNC  93 | ||| | H | D:GLU  90 | ||| | H | D:GLU  90 | ||| | H | D:GLU  90 | ||| | H | D:GLU  90 | ||| | H | D:GLU  90 | ||| | H | D:GLU  90 | ||| | H | D:GLU  90 | ||| | H | D:GLU  90 | ||| | H | D:GLU  90 |
| H | D:ASP  94 | ||| | H | D:LEU  91 | ||| | H | D:LEU  91 | ||| | H | D:LEU  91 | ||| | H | D:LEU  91 | ||| | H | D:LEU  91 | ||| | H | D:LEU  91 | ||| | H | D:LEU  91 | ||| | H | D:LEU  91 | ||| | H | D:LEU  91 |
| H | D:LYS  95 | ||| | H | D:HIS  92 | ||| | H | D:HIS  92 | ||| | H | D:HIS  92 | ||| | H | D:HIS  92 | ||| | H | D:HIS  92 | ||| | H | D:HIS  92 | ||| | H | D:HIS  92 | ||| | H | D:HIS  92 | ||| | H | D:HIS  92 |
|  | D:LEU  96 | ||| | H | D:CYS  93 | ||| | H | D:CYS  93 | ||| | H | D:CYS  93 | ||| | H | D:CYS  93 | ||| | H | D:CYS  93 | ||| | H | D:CYS  93 | ||| | H | D:CYS  93 | ||| | H | D:CYS  93 | ||| | H | D:CYS  93 |
|  | |  | H | D:ASP  94 |  | H | D:ASP  94 |  | H | D:ASP  94 |  | H | D:ASP  94 |  | H | D:ASP  94 |  | H | D:ASP  94 |  | H | D:ASP  94 |  | H | D:ASP  94 |  | H | D:ASP  94 |
|  | |  | H | D:LYS  95 |  | H | D:LYS  95 |  | H | D:LYS  95 |  | H | D:LYS  95 |  | H | D:LYS  95 |  | H | D:LYS  95 |  | H | D:LYS  95 |  | H | D:LYS  95 |  | H | D:LYS  95 |
|  | |  |  | D:LEU  96 |  |  | D:LEU  96 |  |  | D:LEU  96 |  |  | D:LEU  96 |  |  | D:LEU  96 |  |  | D:LEU  96 |  |  | D:LEU  96 |  |  | D:LEU  96 |  |  | D:LEU  96 |
|  | |  |  | D:HIS  97 |  |  | D:HIS  97 |  |  | D:HIS  97 |  |  | D:HIS  97 |  |  | D:HIS  97 |  |  | D:HIS  97 |  |  | D:HIS  97 |  |  | D:HIS  97 |  |  | D:HIS  97 |
|  | D:HIS  97 | ||| |  | D:VAL  98 | ||| |  | D:VAL  98 | ||| |  | D:VAL  98 | ||| |  | D:VAL  98 | ||| |  | D:VAL  98 | ||| |  | D:VAL  98 | ||| |  | D:VAL  98 | ||| |  | D:VAL  98 | ||| |  | D:VAL  98 |
|  | D:VAL  98 | ||| |  | D:ASP  99 | ||| |  | D:ASP  99 | ||| |  | D:ASP  99 | ||| |  | D:ASP  99 | ||| |  | D:ASP  99 | ||| |  | D:ASP  99 | ||| |  | D:ASP  99 | ||| |  | D:ASP  99 | ||| |  | D:ASP  99 |
|  | D:ASP  99 |  |  | |  |  | |  |  | |  |  | |  |  | |  |  | |  |  | |  |  | |  |  | |
| H | D:PRO 100 | ||| | H | D:PRO 100 | ||| | H | D:PRO 100 | ||| | H | D:PRO 100 | ||| | H | D:PRO 100 | ||| | H | D:PRO 100 | ||| | H | D:PRO 100 | ||| | H | D:PRO 100 | ||| | H | D:PRO 100 | ||| | H | D:PRO 100 |
| H | D:GLU 101 | ||| | H | D:GLU 101 | ||| | H | D:GLU 101 | ||| | H | D:GLU 101 | ||| | H | D:GLU 101 | ||| | H | D:GLU 101 | ||| | H | D:GLU 101 | ||| | H | D:GLU 101 | ||| | H | D:GLU 101 | ||| | H | D:GLU 101 |
| H | D:ASN 102 | ||| | H | D:ASN 102 | ||| | H | D:ASN 102 | ||| | H | D:ASN 102 | ||| | H | D:ASN 102 | ||| | H | D:ASN 102 | ||| | H | D:ASN 102 | ||| | H | D:ASN 102 | ||| | H | D:ASN 102 | ||| | H | D:ASN 102 |
| H | D:PHE 103 | ||| | H | D:PHE 103 | ||| | H | D:PHE 103 | ||| | H | D:PHE 103 | ||| | H | D:PHE 103 | ||| | H | D:PHE 103 | ||| | H | D:PHE 103 | ||| | H | D:PHE 103 | ||| | H | D:PHE 103 | ||| | H | D:PHE 103 |
| H | D:ARG 104 | ||| | H | D:ARG 104 | ||| | H | D:ARG 104 | ||| | H | D:ARG 104 | ||| | H | D:ARG 104 | ||| | H | D:ARG 104 | ||| | H | D:ARG 104 | ||| | H | D:ARG 104 | ||| | H | D:ARG 104 | ||| | H | D:ARG 104 |
| H | D:LEU 105 | ||| | H | D:LEU 105 | ||| | H | D:LEU 105 | ||| | H | D:LEU 105 | ||| | H | D:LEU 105 | ||| | H | D:LEU 105 | ||| | H | D:LEU 105 | ||| | H | D:LEU 105 | ||| | H | D:LEU 105 | ||| | H | D:LEU 105 |
| H | D:LEU 106 | ||| | H | D:LEU 106 | ||| | H | D:LEU 106 | ||| | H | D:LEU 106 | ||| | H | D:LEU 106 | ||| | H | D:LEU 106 | ||| | H | D:LEU 106 | ||| | H | D:LEU 106 | ||| | H | D:LEU 106 | ||| | H | D:LEU 106 |
| H | D:GLY 107 | ||| | H | D:GLY 107 | ||| | H | D:GLY 107 | ||| | H | D:GLY 107 | ||| | H | D:GLY 107 | ||| | H | D:GLY 107 | ||| | H | D:GLY 107 | ||| | H | D:GLY 107 | ||| | H | D:GLY 107 | ||| | H | D:GLY 107 |
| H | D:ASN 108 | ||| | H | D:ASN 108 | ||| | H | D:ASN 108 | ||| | H | D:ASN 108 | ||| | H | D:ASN 108 | ||| | H | D:ASN 108 | ||| | H | D:ASN 108 | ||| | H | D:ASN 108 | ||| | H | D:ASN 108 | ||| | H | D:ASN 108 |
| H | D:VAL 109 | ||| | H | D:VAL 109 | ||| | H | D:VAL 109 | ||| | H | D:VAL 109 | ||| | H | D:VAL 109 | ||| | H | D:VAL 109 | ||| | H | D:VAL 109 | ||| | H | D:VAL 109 | ||| | H | D:VAL 109 | ||| | H | D:VAL 109 |
| H | D:LEU 110 | ||| | H | D:LEU 110 | ||| | H | D:LEU 110 | ||| | H | D:LEU 110 | ||| | H | D:LEU 110 | ||| | H | D:LEU 110 | ||| | H | D:LEU 110 | ||| | H | D:LEU 110 | ||| | H | D:LEU 110 | ||| | H | D:LEU 110 |
| H | D:VAL 111 | ||| | H | D:VAL 111 | ||| | H | D:VAL 111 | ||| | H | D:VAL 111 | ||| | H | D:VAL 111 | ||| | H | D:VAL 111 | ||| | H | D:VAL 111 | ||| | H | D:VAL 111 | ||| | H | D:VAL 111 | ||| | H | D:VAL 111 |
| H | D:CYS 112 | ||| | H | D:CYS 112 | ||| | H | D:CYS 112 | ||| | H | D:CYS 112 | ||| | H | D:CYS 112 | ||| | H | D:CYS 112 | ||| | H | D:CYS 112 | ||| | H | D:CYS 112 | ||| | H | D:CYS 112 | ||| | H | D:CYS 112 |
| H | D:VAL 113 | ||| | H | D:VAL 113 | ||| | H | D:VAL 113 | ||| | H | D:VAL 113 | ||| | H | D:VAL 113 | ||| | H | D:VAL 113 | ||| | H | D:VAL 113 | ||| | H | D:VAL 113 | ||| | H | D:VAL 113 | ||| | H | D:VAL 113 |
| H | D:LEU 114 | ||| | H | D:LEU 114 | ||| | H | D:LEU 114 | ||| | H | D:LEU 114 | ||| | H | D:LEU 114 | ||| | H | D:LEU 114 | ||| | H | D:LEU 114 | ||| | H | D:LEU 114 | ||| | H | D:LEU 114 | ||| | H | D:LEU 114 |
| H | D:ALA 115 | ||| | H | D:ALA 115 | ||| | H | D:ALA 115 | ||| | H | D:ALA 115 | ||| | H | D:ALA 115 | ||| | H | D:ALA 115 | ||| | H | D:ALA 115 | ||| | H | D:ALA 115 | ||| | H | D:ALA 115 | ||| | H | D:ALA 115 |
| H | D:HIS 116 | ||| | H | D:HIS 116 | ||| | H | D:HIS 116 | ||| | H | D:HIS 116 | ||| | H | D:HIS 116 | ||| | H | D:HIS 116 | ||| | H | D:HIS 116 | ||| | H | D:HIS 116 | ||| | H | D:HIS 116 | ||| | H | D:HIS 116 |
| H | D:HIS 117 | ||| | H | D:HIS 117 | ||| | H | D:HIS 117 | ||| | H | D:HIS 117 | ||| | H | D:HIS 117 | ||| | H | D:HIS 117 | ||| | H | D:HIS 117 | ||| | H | D:HIS 117 | ||| | H | D:HIS 117 | ||| | H | D:HIS 117 |
| H | D:PHE 118 | ||| | H | D:PHE 118 | ||| | H | D:PHE 118 | ||| | H | D:PHE 118 | ||| | H | D:PHE 118 | ||| | H | D:PHE 118 | ||| | H | D:PHE 118 | ||| | H | D:PHE 118 | ||| | H | D:PHE 118 | ||| | H | D:PHE 118 |
| H | D:GLY 119 | ||| | H | D:GLY 119 | ||| | H | D:GLY 119 | ||| | H | D:GLY 119 | ||| | H | D:GLY 119 | ||| | H | D:GLY 119 | ||| | H | D:GLY 119 | ||| | H | D:GLY 119 | ||| | H | D:GLY 119 | ||| | H | D:GLY 119 |
|  | D:LYS 120 | ||| |  | D:LYS 120 | ||| |  | D:LYS 120 | ||| |  | D:LYS 120 | ||| |  | D:LYS 120 | ||| |  | D:LYS 120 | ||| |  | D:LYS 120 | ||| |  | D:LYS 120 | ||| |  | D:LYS 120 | ||| |  | D:LYS 120 |
|  | D:GLU 121 | ||| |  | D:GLU 121 | ||| |  | D:GLU 121 | ||| |  | D:GLU 121 | ||| |  | D:GLU 121 | ||| |  | D:GLU 121 | ||| |  | D:GLU 121 | ||| |  | D:GLU 121 | ||| |  | D:GLU 121 | ||| |  | D:GLU 121 |
|  | D:PHE 122 | ||| |  | D:PHE 122 | ||| |  | D:PHE 122 | ||| |  | D:PHE 122 | ||| |  | D:PHE 122 | ||| |  | D:PHE 122 | ||| |  | D:PHE 122 | ||| |  | D:PHE 122 | ||| |  | D:PHE 122 | ||| |  | D:PHE 122 |
| H | D:THR 123 |  |  | |  |  | |  |  | |  |  | |  |  | |  |  | |  |  | |  |  | |  |  | |
| H | D:PRO 124 |  |  | |  |  | |  |  | |  |  | |  |  | |  |  | |  |  | |  |  | |  |  | |
| H | D:PRO 125 |  |  | |  |  | |  |  | |  |  | |  |  | |  |  | |  |  | |  |  | |  |  | |
| H | D:VAL 126 | ||| | H | D:THR 123 | ||| | H | D:THR 123 | ||| | H | D:THR 123 | ||| | H | D:THR 123 | ||| | H | D:THR 123 | ||| | H | D:THR 123 | ||| | H | D:THR 123 | ||| | H | D:THR 123 | ||| | H | D:THR 123 |
| H | D:GLN 127 | ||| | H | D:PRO 124 | ||| | H | D:PRO 124 | ||| | H | D:PRO 124 | ||| | H | D:PRO 124 | ||| | H | D:PRO 124 | ||| | H | D:PRO 124 | ||| | H | D:PRO 124 | ||| | H | D:PRO 124 | ||| | H | D:PRO 124 |
| H | D:ALA 128 | ||| | H | D:PRO 125 | ||| | H | D:PRO 125 | ||| | H | D:PRO 125 | ||| | H | D:PRO 125 | ||| | H | D:PRO 125 | ||| | H | D:PRO 125 | ||| | H | D:PRO 125 | ||| | H | D:PRO 125 | ||| | H | D:PRO 125 |
| H | D:ALA 129 | ||| | H | D:VAL 126 | ||| | H | D:VAL 126 | ||| | H | D:VAL 126 | ||| | H | D:VAL 126 | ||| | H | D:VAL 126 | ||| | H | D:VAL 126 | ||| | H | D:VAL 126 | ||| | H | D:VAL 126 | ||| | H | D:VAL 126 |
| H | D:TYR 130 | ||| | H | D:GLN 127 | ||| | H | D:GLN 127 | ||| | H | D:GLN 127 | ||| | H | D:GLN 127 | ||| | H | D:GLN 127 | ||| | H | D:GLN 127 | ||| | H | D:GLN 127 | ||| | H | D:GLN 127 | ||| | H | D:GLN 127 |
| H | D:GLN 131 | ||| | H | D:ALA 128 | ||| | H | D:ALA 128 | ||| | H | D:ALA 128 | ||| | H | D:ALA 128 | ||| | H | D:ALA 128 | ||| | H | D:ALA 128 | ||| | H | D:ALA 128 | ||| | H | D:ALA 128 | ||| | H | D:ALA 128 |
| H | D:LYS 132 | ||| | H | D:ALA 129 | ||| | H | D:ALA 129 | ||| | H | D:ALA 129 | ||| | H | D:ALA 129 | ||| | H | D:ALA 129 | ||| | H | D:ALA 129 | ||| | H | D:ALA 129 | ||| | H | D:ALA 129 | ||| | H | D:ALA 129 |
| H | D:VAL 133 | ||| | H | D:TYR 130 | ||| | H | D:TYR 130 | ||| | H | D:TYR 130 | ||| | H | D:TYR 130 | ||| | H | D:TYR 130 | ||| | H | D:TYR 130 | ||| | H | D:TYR 130 | ||| | H | D:TYR 130 | ||| | H | D:TYR 130 |
| H | D:VAL 134 | ||| | H | D:GLN 131 | ||| | H | D:GLN 131 | ||| | H | D:GLN 131 | ||| | H | D:GLN 131 | ||| | H | D:GLN 131 | ||| | H | D:GLN 131 | ||| | H | D:GLN 131 | ||| | H | D:GLN 131 | ||| | H | D:GLN 131 |
| H | D:ALA 135 | ||| | H | D:LYS 132 | ||| | H | D:LYS 132 | ||| | H | D:LYS 132 | ||| | H | D:LYS 132 | ||| | H | D:LYS 132 | ||| | H | D:LYS 132 | ||| | H | D:LYS 132 | ||| | H | D:LYS 132 | ||| | H | D:LYS 132 |
| H | D:GLY 136 | ||| | H | D:VAL 133 | ||| | H | D:VAL 133 | ||| | H | D:VAL 133 | ||| | H | D:VAL 133 | ||| | H | D:VAL 133 | ||| | H | D:VAL 133 | ||| | H | D:VAL 133 | ||| | H | D:VAL 133 | ||| | H | D:VAL 133 |
| H | D:VAL 137 | ||| | H | D:VAL 134 | ||| | H | D:VAL 134 | ||| | H | D:VAL 134 | ||| | H | D:VAL 134 | ||| | H | D:VAL 134 | ||| | H | D:VAL 134 | ||| | H | D:VAL 134 | ||| | H | D:VAL 134 | ||| | H | D:VAL 134 |
| H | D:ALA 138 | ||| | H | D:ALA 135 | ||| | H | D:ALA 135 | ||| | H | D:ALA 135 | ||| | H | D:ALA 135 | ||| | H | D:ALA 135 | ||| | H | D:ALA 135 | ||| | H | D:ALA 135 | ||| | H | D:ALA 135 | ||| | H | D:ALA 135 |
| H | D:ASN 139 | ||| | H | D:GLY 136 | ||| | H | D:GLY 136 | ||| | H | D:GLY 136 | ||| | H | D:GLY 136 | ||| | H | D:GLY 136 | ||| | H | D:GLY 136 | ||| | H | D:GLY 136 | ||| | H | D:GLY 136 | ||| | H | D:GLY 136 |
| H | D:ALA 140 | ||| | H | D:VAL 137 | ||| | H | D:VAL 137 | ||| | H | D:VAL 137 | ||| | H | D:VAL 137 | ||| | H | D:VAL 137 | ||| | H | D:VAL 137 | ||| | H | D:VAL 137 | ||| | H | D:VAL 137 | ||| | H | D:VAL 137 |
| H | D:LEU 141 | ||| | H | D:ALA 138 | ||| | H | D:ALA 138 | ||| | H | D:ALA 138 | ||| | H | D:ALA 138 | ||| | H | D:ALA 138 | ||| | H | D:ALA 138 | ||| | H | D:ALA 138 | ||| | H | D:ALA 138 | ||| | H | D:ALA 138 |
| H | D:ALA 142 | ||| | H | D:ASN 139 | ||| | H | D:ASN 139 | ||| | H | D:ASN 139 | ||| | H | D:ASN 139 | ||| | H | D:ASN 139 | ||| | H | D:ASN 139 | ||| | H | D:ASN 139 | ||| | H | D:ASN 139 | ||| | H | D:ASN 139 |
| H | D:HIS 143 | ||| | H | D:ALA 140 | ||| | H | D:ALA 140 | ||| | H | D:ALA 140 | ||| | H | D:ALA 140 | ||| | H | D:ALA 140 | ||| | H | D:ALA 140 | ||| | H | D:ALA 140 | ||| | H | D:ALA 140 | ||| | H | D:ALA 140 |
|  | D:LYS 144 | ||| | H | D:LEU 141 | ||| | H | D:LEU 141 | ||| | H | D:LEU 141 | ||| | H | D:LEU 141 | ||| | H | D:LEU 141 | ||| | H | D:LEU 141 | ||| | H | D:LEU 141 | ||| | H | D:LEU 141 | ||| | H | D:LEU 141 |
|  | |  | H | D:ALA 142 |  | H | D:ALA 142 |  | H | D:ALA 142 |  | H | D:ALA 142 |  | H | D:ALA 142 |  | H | D:ALA 142 |  | H | D:ALA 142 |  | H | D:ALA 142 |  | H | D:ALA 142 |
|  | |  | H | D:HIS 143 |  | H | D:HIS 143 |  | H | D:HIS 143 |  | H | D:HIS 143 |  | H | D:HIS 143 |  | H | D:HIS 143 |  | H | D:HIS 143 |  | H | D:HIS 143 |  | H | D:HIS 143 |
|  | |  |  | D:LYS 144 |  |  | D:LYS 144 |  |  | D:LYS 144 |  |  | D:LYS 144 |  |  | D:LYS 144 |  |  | D:LYS 144 |  |  | D:LYS 144 |  |  | D:LYS 144 |  |  | D:LYS 144 |
|  | |  |  | D:TYR 145 |  |  | D:TYR 145 |  |  | D:TYR 145 |  |  | D:TYR 145 |  |  | D:TYR 145 |  |  | D:TYR 145 |  |  | D:TYR 145 |  |  | D:TYR 145 |  |  | D:TYR 145 |
|  | |  |  | D:HIS 146 |  |  | D:HIS 146 |  |  | D:HIS 146 |  |  | D:HIS 146 |  |  | D:HIS 146 |  |  | D:HIS 146 |  |  | D:HIS 146 |  |  | D:HIS 146 |  |  | D:HIS 146 |
