# Supplementary material for: Protein Molecular Surface Mapped at Different Geometrical Resolutions
Source: PLoS One. 2013 Mar 14;8(3):e58896. doi: 10.1371/journal.pone.0058896 (PMC3597524; doi:10.1371/journal.pone.0058896)
Supplement: File S4 — (DOC) [file pone.0058896.s004.doc]

# **Protein molecular surface mapped at different geometrical resolutions**

**Dan V Nicolau,^1,2^ Ewa Paszek,^1^ Florin Fulga,^1^ Dan V. Nicolau Jr.^3^**

*^1^Department of Electrical Engineering & Electronics, University of Liverpool, Liverpool, UK*

*^2^Department of Bioengineering, McGill University, Montreal, Canada*

*^3^Department of Integrative Biology, University of California at Berkeley, Berkeley, USA*

**Supplementary Information S4**

**Method of calculation of the charges = f(pH) in amino acids**

For each amino acid *AA* that have an ionized form, i.e., cysteine, aspartic acid, glutamic acid, histidine, arginine and tyrosine, the variation of atomic change *vs* pH was calculated as follows:

For instance, to obtain the ratio [Cys-]/[Cys]:

$$\begin{matrix} pH=pKa+\log\left(\left[Cys-\right]/\left[Cys\right]\right) \\ ratio=\left[Cys-\right]/\left[Cys\right]=10E\left(pH-pKa\right) \end{matrix}$$

For each atom in the amino acid *AA*, the atomic charge has been calculated using HyperChem in the ‘extreme’ cases, i.e., when the amino acid *AA* is Neutral (Cys) and when it is Anionic (Cys-).

The atomic charge of a given atom *X* in the amino acid *AA* at a given pH is calculated as follows:

*q(X)* - atomic charge of atom when in the Neutral form (Cys)

*q(X-)* - atomic charge of the atom when the anionic form (Cys)

*q(X)* *total* - atomic charge of the atom for a given pH value

Thus for a given pH:

$$q\left(X\right)total=\left[q\left(X\right)+q\left(X-\right)*ratio\right]/\left(ratio+1\right)$$

Note: The charges for a generic atom X are different in the Anionic (or Cationic) and Neutral form even if this atom is not directly involved in the de-protonation process.
